# Inhibition of CXorf56 promotes PARP inhibitor-induced cytotoxicity in triple-negative breast cancer

**DOI:** 10.1038/s41523-023-00540-3

**Published:** 2023-05-08

**Authors:** Ying Zhu, Zhixian Liu, Liang Gui, Wen Yun, Changfei Mao, Rong Deng, Yufeng Yao, Qiao Yu, Jifeng Feng, Hongxia Ma, Wei Bao

**Affiliations:** 1 Department of General Surgery, Jiangsu Cancer Hospital & Jiangsu Institute of Cancer Research & The Affiliated Cancer Hospital of Nanjing Medical University, Nanjing, China; 2 Department of Pharmacy, Jiangsu Cancer Hospital & Jiangsu Institute of Cancer Research & The Affiliated Cancer Hospital of Nanjing Medical University, Nanjing, China; 3 Department of Epidemiology, Center for Global Health, School of Public Health, Jiangsu Key Lab of Cancer Biomarkers, Prevention and Treatment, Collaborative Innovation Center for Cancer Personalized Medicine, Nanjing Medical University, Nanjing, China; 4 Department of Pathology, Jinling Hospital, Affiliated Hospital of Medical School, Nanjing University, Nanjing, China

**Keywords:** Breast cancer, Cancer therapeutic resistance

## Abstract

Poly(ADP-ribose) polymerase inhibitors (PARPis) induce DNA lesions that preferentially kill homologous recombination (HR)-deficient breast cancers induced by *BRCA* mutations, which exhibit a low incidence in breast cancer, thereby limiting the benefits of PARPis. Additionally, breast cancer cells, particularly triple-negative breast cancer (TNBC) cells, exhibit HR and PARPi resistance. Therefore, targets must be identified for inducing HR deficiency and sensitizing cancer cells to PARPis. Here, we reveal that *CXorf56* protein increased HR repair in TNBC cells by interacting with the Ku70 DNA-binding domain, reducing Ku70 recruitment and promoting RPA32, BRCA2, and RAD51 recruitment to sites of DNA damage. Knockdown of *CXorf56* protein suppressed HR in TNBC cells, specifically during the S and G2 phases, and increased cell sensitivity to olaparib in vitro and in vivo. Clinically, *CXorf56* protein was upregulated in TNBC tissues and associated with aggressive clinicopathological characteristics and poor survival. All these findings indicate that treatment designed to inhibit *CXorf56* protein in TNBC combined with PARPis may overcome drug resistance and expand the application of PARPis to patients with non-*BRCA* mutantion.

## Introduction

The incidence of breast cancer has markedly increased, and it is the most common malignancy worldwide^1^. Over the past few decades, the mortality rate of breast cancer has declined by 40%, owing to health screening, individualized management, and precision medicine^2^. Targeted therapies have altered and optimized treatment strategies for certain breast cancer, such as HER2-positive breast cancer. Triple-negative breast cancer (TNBC) is defined by the lack of estrogen receptor (ER), progesterone receptor (PR), and HER2 expression, which cannot be treated using targeted or hormonal therapies. TNBC has a poorer prognosis with shorter disease-free survival and overall survival (OS), and a higher risk of recurrence than HER2-positive and hormone receptor-positive breast cancer, even under optimal tri-modality treatment^3,4^. Therefore, new therapeutic methods for TNBC are urgently needed.

Recently, poly(ADP-ribose) polymerase (PARP) inhibitors (PARPis) have been approved by the US Food and Drug Administration for homologous recombination (HR)-deficient breast cancer induced by *BRCA1/2* mutations^5,6^. PARPs are a class of enzymes activated by single- and double-strand DNA breaks (SSBs and DSBs); they catalyze the transfer of ADP-ribose from nicotinamide adenine dinucleotide (NAD+) to protein^7^. PARPis induce SSBs accumulation, leading to replication fork collapse during the S phase and subsequent DSBs^8^. During the S phase, DSBs are normally repaired by HR; however, in HR-defective (HRD) cells such as *BRCA1/2*-mutated cells, DSBs tend to be repaired by error-prone, non-homologous end joining (NHEJ), which causes deleterious DNA damage, resulting in cell death^9^. Additionally, PARPis also trap PARP1 and PARP2 in damaged DNA, forming PARP-DNA complexes and induce cytotoxicity^10,11^. Therefore, PARPis instigate synthetic lethality in HR-deficient cells, which have been used for treating patients with HR-deficient breast cancer^12^. High chromosomal instability is a hallmark of TNBC, and serves as a therapeutic target to enhance PARPi sensitivity^13^. However, TNBC develops PARPi resistance mechanisms, such as HR alteration, which limits the clinical efficacy of PARPi for the treatment of patients with TNBC. In addition, low incidence of *BRCA1/2* mutations in TNBC limits the benefits of PARPis, and most patients experience early recurrence and distant metastasis due to PARPi resistance^14^. Therefore, novel targets are required to induce strong and stable HRD status with “*BRCA* mutation-like effects” to expand the application of PARPis for the treatment of patients with non-*BRCA* mutants and reverse PARPis resistance in those with *BRCA*-mutants.

The chromosome X open reading frame 56 gene (*CXorf56*) is located on human chromosome Xq24 in a region where genomic alterations have been reported in patients with syndromic intellectual disability^15^. Neuron-expressed *CXorf56* protein is mainly localized in the cell nucleus and cytoplasm and is reportedly involved in X-inactivation^16^. Further, endoplasmic reticulum (ER)-expressed *CXorf56* protein interacts with STING protein, which is crucial for STING signalling activation in immune cells^17^. And *CXorf56* protein is therefore also known as the STING ER exit protein 1 (STEEP1)”. The role of *CXorf56* protein in cancers remains unknown, indicating the need for further research. Therefore, we explored the functional role and molecular mechanism of *CXorf56* protein as a potential therapeutic target in breast cancer.

## Results

### *CXorf56* protein is overexpressed in TNBC and is essential for HR repair in tumors

**Fig. 1:**
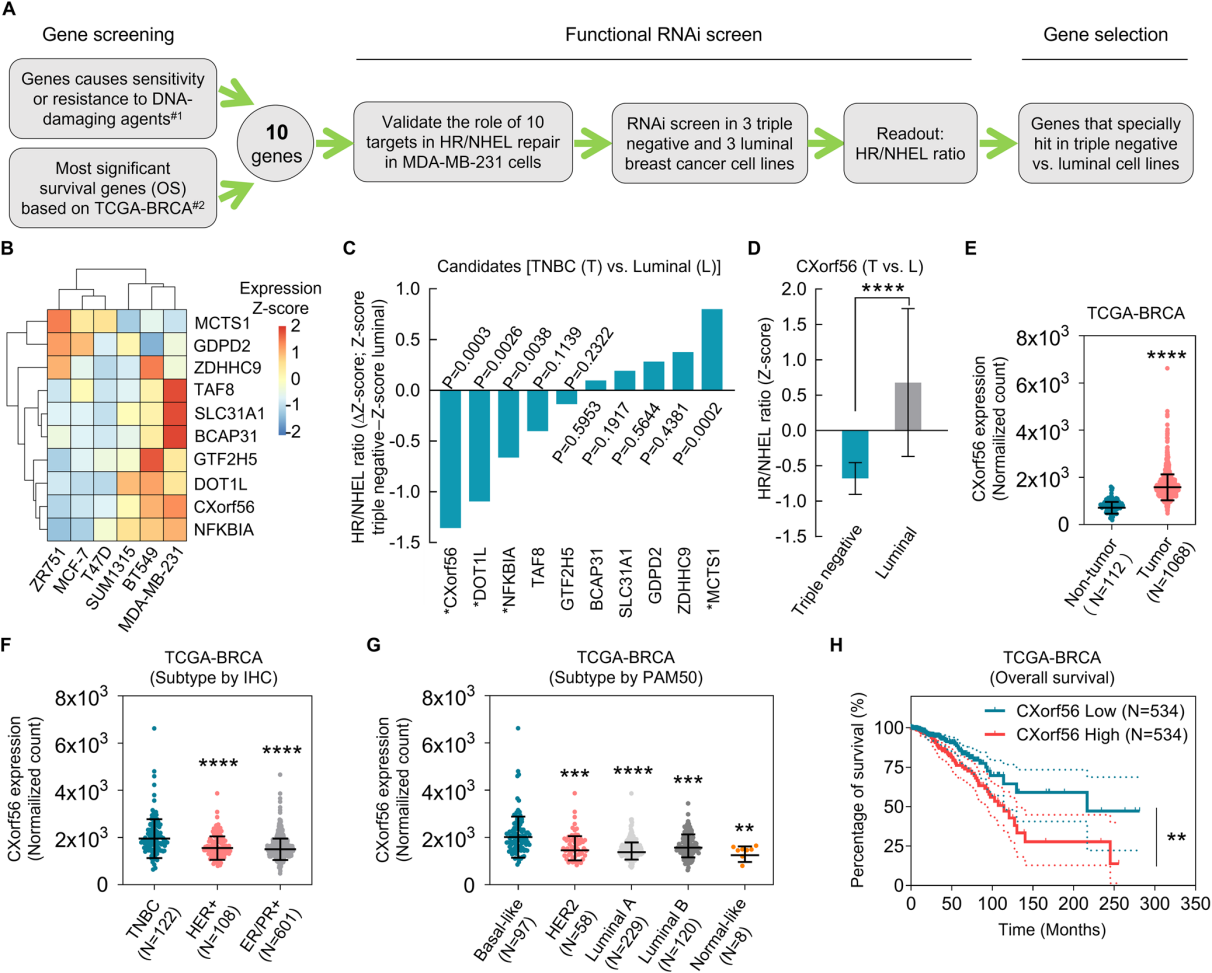
RNAi screening and Kaplan-Meier plot strategies to identify DNA damage repair regulators of TNBC. **A** The schematic diagram highlights the criteria for gene selection and the experimental setup for the RNAi screen. ^#1^890 DDR-related genes were identified based on the CRISPR-Cas9 screens against 27 genotoxic agents^18^. ^#2^ The top 500 overall-survival-related genes were directly downloaded through GEPIA portal based on the TCGA-BRCA database^45^. **B** The heat map that represents the expression of selected genes in different BC cells was determined by qPCR analysis. **C** The bar chart shows the average Z-score of TNBC cell lines minus the average Z-score of luminal cell lines (ΔZ-score) for all the genes included in the RNAi screen. A negative value indicates that the siRNA decreased the HR/NHEJ ratio more in the TNBC cell lines (*P < 0.05 comparing TNBC and luminal Z-scores). Data were analyzed by a two-tailed *t* test. **D** The average TNBC (T) and luminal (L) Z-scores for all cell lines for *CXorf56*. The *P*-value (****P < 0.0001) indicates a statistically significant difference between TNBC and luminal lines. Data were analyzed by a two-tailed *t* test. **E** The difference in *CXorf56* transcript levels between normal breast and cancer tissues is presented as a scatter dot plot. Data were analyzed by a two-tailed *t* test. Scatter dot plots show the transcript levels of *CXorf56* across the breast cancer subtypes based on the IHC (**F**) or PAM50 (**G**) classification of the TCGA-BRCA cohort. Data were analyzed by a two-tailed *t* test. **H** Kaplan-Meier survival curve shows a poorer OS rate in BC patients with high *CXorf56* transcript levels than those with low *CXorf56* transcript levels. Data were analyzed by a log-rank test. Remarks: *p < 0.05, **p < 0.01, ***p < 0.001, ****P < 0.0001; Data are presented as mean ± SD.

HR plays an essential role in error-free DNA DSB repair, thereby maintaining genomic stability. Cancer cells develop multiple mechanisms to enhance HR repair and induce drug resistance. Therefore, inducing HRD is a viable strategy to sensitize cancer cells to DNA-damaging therapies and overcome therapeutic resistance. CRISPR/Cas9 screens have identified 890 genes whose loss causes either sensitivity or resistance to DNA-damaging agents^18^, likely involved in DNA damage response (DDR) pathways. To identify potential DNA repair driver genes in breast cancer progression, an intersection between DDR-related genes and breast cancer survival-related genes (the top 500 significantly OS-related genes were downloaded from the GEPIA portal based on breast cancer datasets from The Cancer Genome Atlas database) was determined using a Venn diagram (Supplementary Fig. 1a). In this gene set, 10 DDR-related candidates were associated with the prognosis of patients with breast cancer (Fig. 1A and Supplementary Fig. 1a). To identify the genes that regulate HR repair, we knocked down the candidate genes, in MDA-MB-231 cells, and performed the first set of functional screening using a tailored siRNA library and the SeeSaw 2.0 Reporter (SSR2.0) system^19^. TAF8, GTF2H5, DOT1L, *CXorf56* protein, and NFKBIA knockdown reduced the HR/NHEJ ratio in MDA-MB-231 cells (Supplementary Fig. 1b, c). MDA-MB-231 is a TNBC cell line, therefore, to determine whether the role of the genes in DDR are cell-line-specific, we performed functional siRNA screening in two additional TNBC cell lines and three non-TNBC cell lines. The TNBC and luminal cell lines harboring SSR 2.0 reporters were subjected to a siRNA library targeting these candidates; HR and NHEJ efficiencies were subsequently measured to identify genes selectively required for HR repair in TNBC (Fig. 1A). The expression of the selected genes in different cells was determined by qPCR analysis (Fig. 1B). The average Z-score was calculated for genes with a differential HR/NHEJ ratio between that of TNBC and luminal breast cancer cell lines (Supplementary Dataset 1). *CXorf56* met the most significant criteria of scoring at a ΔZ-score level (TNBC cell line Z-score – luminal cell line Z-score) (Fig. 1C, d). After selection, the clinical value of *CXorf56* was first evaluated using the TCGA-BRCA dataset. Analysis of the TCGA-BRCA cohort demonstrated that *CXorf56* was significantly upregulated in breast cancer tissues (Fig. 1E). Notably, higher *CXorf56* transcript levels were observed in the TNBC tissues (immunohistochemistry subtype) or basal-like breast cancer tissues (PAM50 subtype) than in other breast cancer tissues (Fig. 1F, G). Further, patients with *CXorf56* amplification tended to have shorter OS (Fig. 1H). Collectively, our results suggest that the potential HR regulator *CXorf56* is overexpressed in TNBC. Therefore, it was selected for further evaluation.

### *CXorf56* protein expression is positively associated with poor clinical outcomes in patients with breast cancer

**Fig. 2:**
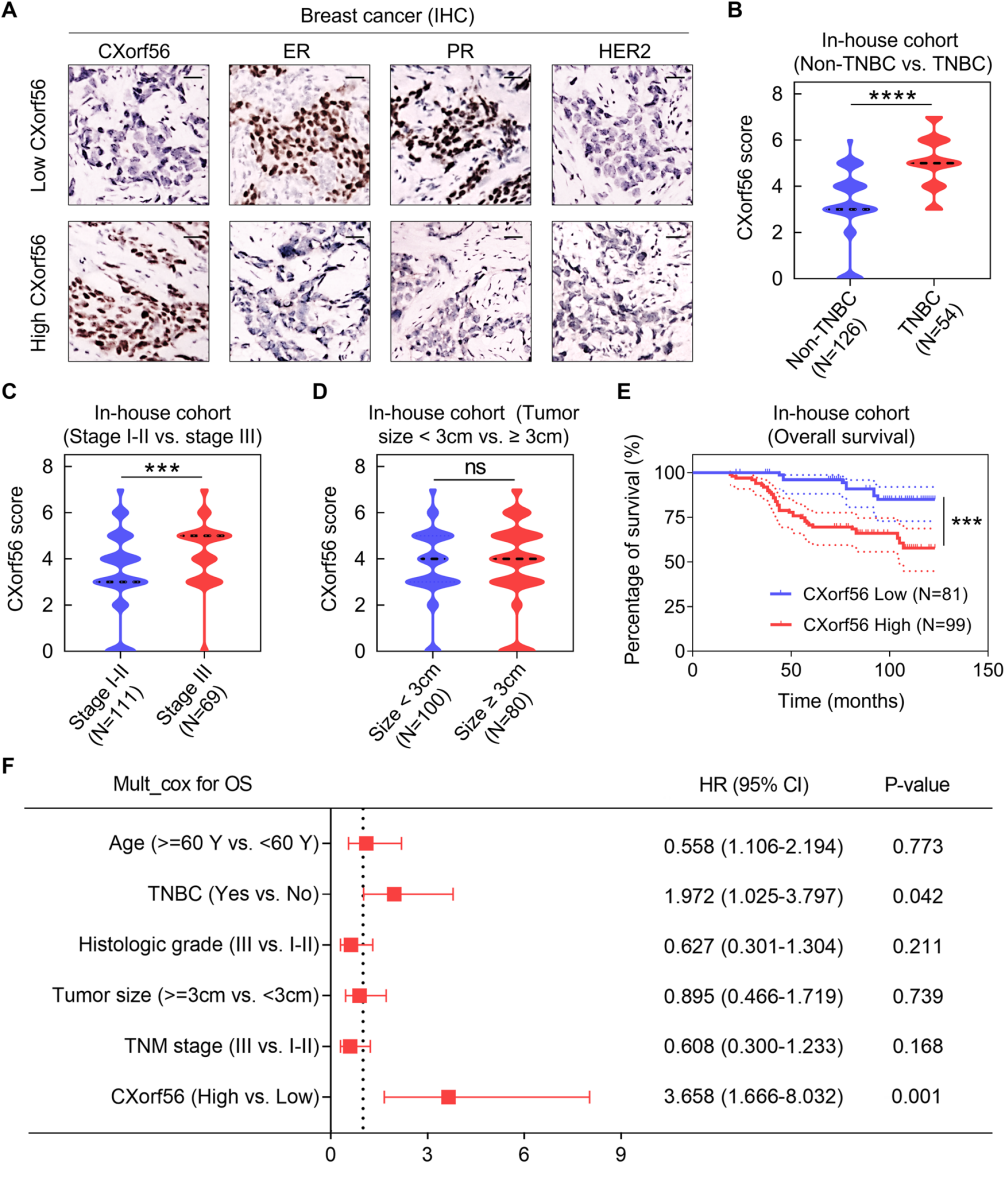
Upregulation of *CXorf56* protein correlates with a poor prognosis for human BC. **A** Representative IHC images of *CXorf56* protein expression (nuclear staining pattern) in non-TNBC tissues and TNBC tissues; Scale bar = 50 μm. The scores indicate *CXorf56* protein levels in tumor tissues in different subgroups (**B**, non-TNBC vs. TNBC; **C**, stage I-II vs. stage III; **D**, tumor size <3 cm vs. tumor size >3 cm). The scores were calculated by the intensity and percentage of stained cells as described in the methods. Data were analyzed by a two-tailed *t* test. **E** BC patients with high *CXorf56* protein expression (score of 4–7) have poorer OS than patients with low *CXorf56* protein expression (score of 0–3). Data were analyzed by a log-rank test. **F** The expression of *CXorf56* protein is an independent prognostic factor for OS of BC patients, based on the multivariate Cox proportional hazard model, which is shown in the forest plot. Data were analyzed by a Wald test. Remarks: ^ns^*p* ≥ 0.05, ***p < 0.001, ****P < 0.0001.

To further investigate the association between breast cancer development and *CXorf56* protein expression, a cohort study of *CXorf56* protein expression was performed in 180 breast cancer tissues with complete follow-up data. *CXorf56* protein expression was considerably upregulated in TNBC tissues than in non-TNBC tissues (Fig. 2A, B), consistent with the transcriptome analysis results (Fig. 1F, G). Further, the *CXorf56* protein levels were relatively higher in stage III breast cancer tumors than in stage I/II tissues, indicating that *CXorf56* protein expression is associated with the malignant progression of breast cancer (Fig. 2C). However, there was no significant difference in *CXorf56* protein levels between breast cancer tissues with different tumor sizes (<3 cm *vs*. ≥3 cm) (Fig. 2D). Kaplan–Meier analysis showed that patients with breast cancer having high *CXorf56* protein levels tended to have a shorter OS (Fig. 2E). The univariate analyses showed that TNBC, high histologic grade, advanced TNM stage, and high *CXorf56* protein expression were the risk factors positively associated with BC prognosis (Supplementary Fig. 2a). Cox proportional hazards regression analyses further showed that *CXorf56* protein overexpression was an independent prognostic predictor of OS (hazard ratio = 3.658, *p* = 0.001) (Fig. 2F). In addition, our cox regression model revealed that TNBC was the independent risk factor positively associated with BC prognosis (Fig. 2F). In BC cancer patients, TNBC was positively associated with histologic grade and TNM stage (Supplementary Fig. 2b), indicating that TNBC reduces breast cancer progression time and shortens patient survival time. Collectively, these results indicate that *CXorf56* protein overexpression is significantly associated with poor prognosis in breast cancer.

### *CXorf56* protein inhibits NHEJ and promotes HR, increasing resistance to DNA-damaging agents

**Fig. 3:**
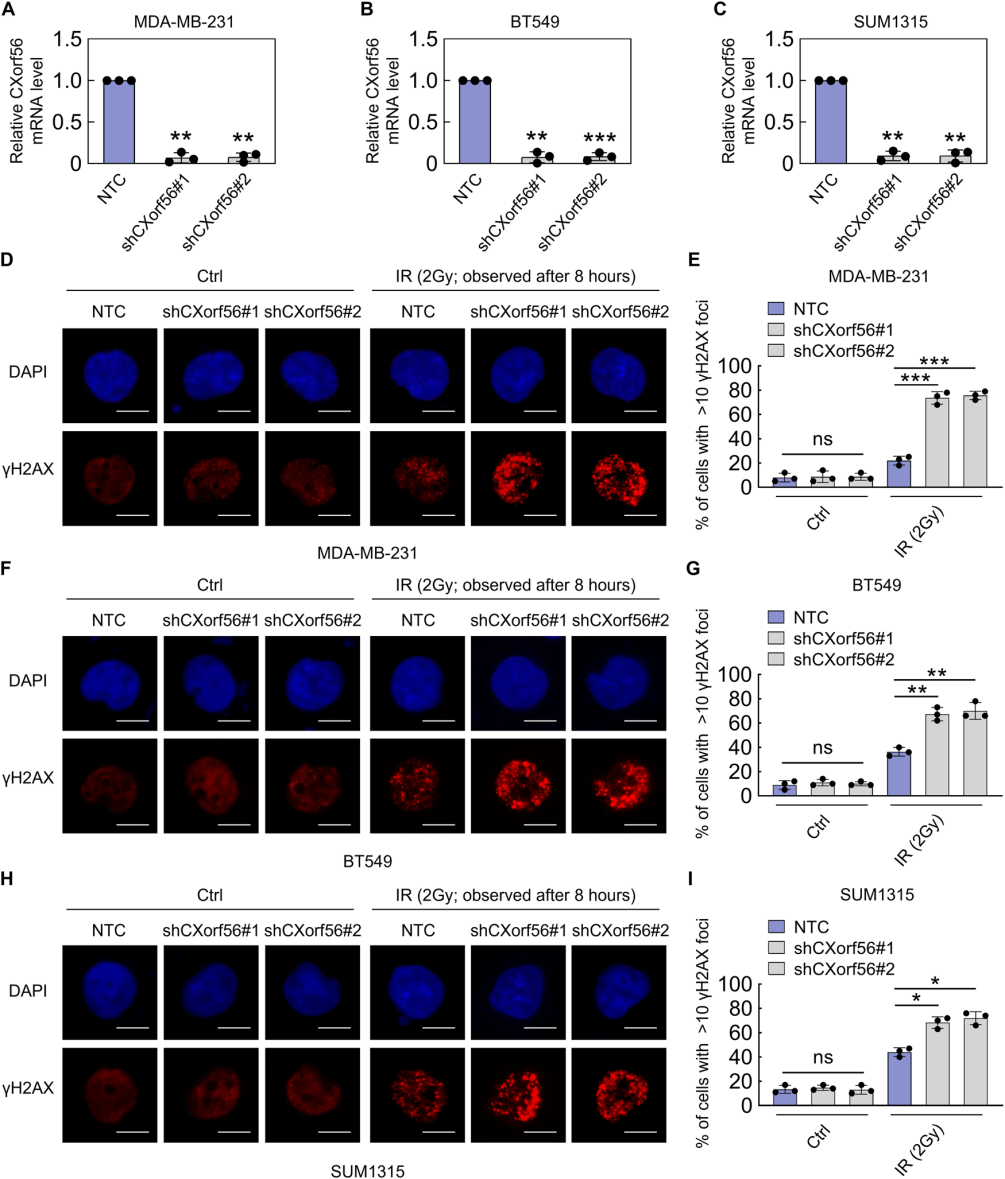
*CXorf56* knockdown promotes the accumulation of DNA damage in TNBC cells upon IR treatment. *CXorf56* stable knockdown efficiency was detected by qPCR in TNBC cells including MDA-MB-231 (**A**), BT549 (**B**), and SUM1315 (**C**) cells, respectively. Data were analyzed by a two-tailed *t* test. Negative control (NTC) and *CXorf56*-knockdown TNBC cells (**D**, **E**, MDA-MB-231; **F**, **G**, BT549; **H**, **I**, SUM1315) were treated without (Ctrl) or with IR (2 Gy), γ-H2AX foci before or 8 hours after IR was detected by immunofluorescence. Nuclei were visualized with DAPI (blue). Representative images of γ-H2AX foci are shown in **D**, **F**, **H**. Quantification of focus signals is shown in **E**, **G**, **I**. Data were analyzed by ANOVA and two-tailed *t* test. Remarks: *p < 0.05, **p < 0.01, ***p < 0.001; Data are presented as mean ± SD. Scale bars = 10 μm.

Given the potential role of *CXorf56* protein in DDR in TNBC cells, we first examined γ-H2AX focus formation, a pan-DNA damage marker, in parental and *CXorf56*-depleted TNBC cells (Fig. 3A–C) exposed to ionizing radiation (IR). As shown in Fig. 3D–I, depletion of *CXorf56* protein resulted in increased γH2AX focus accumulation (8 h) in TNBC cells. Interestingly, γH2AX foci accumulation was more significant in TNBC cells (MDA-MB-231) with relatively high levels of *CXorf56* protein than in TNBC cells (SUM1315) with relatively low levels of *CXorf56* protein (Figs. 1B and 3D, E, H, I), suggesting that high expression of *CXorf56* protein is essential for the enhanced effect of *CXorf56*-knockdown on IR cytotoxicity in TNBC cells. Notably, all three TNBC cell lines, with *CXorf56*-knockdown, showed increased IR, Cisplatin, and Olaparib sensitivity (Supplementary Fig. 3a–i), indicating that reducing *CXorf56* protein expression and inducing DNA damage may serve as a promising TNBC therapeutic strategy. Using pulsed-field gel electrophoresis (PFGE), we showed that DNA damage in olaparib-treated *CXorf56*-knockdown cells was significantly higher than that observed in the NTC group (Supplementary Fig. 3j). These results strongly suggest that *CXorf56*-knockdown induced significant genome instability upon TNBC olaparib treatment. The DNA-dependent protein kinase (DNA-PK) complex plays a pivotal role in NHEJ repair^20^. Therefore, we investigated the effect of *CXorf56*-knockdown with a DNA-PK inhibitor (NU7441). We found that, upon NU7441 treatment, there was no significant difference in NU7441 responsiveness and γH2AX focus formation between NTC and *CXorf56*-knockdown cells (Supplementary Fig. 3k–m). This indicates that suppression of NHEJ repair, using DNA-PK inhibitors, combined with *CXorf56*-knockdown does not serve as a good TNBC therapeutic strategy.

**Fig. 4:**
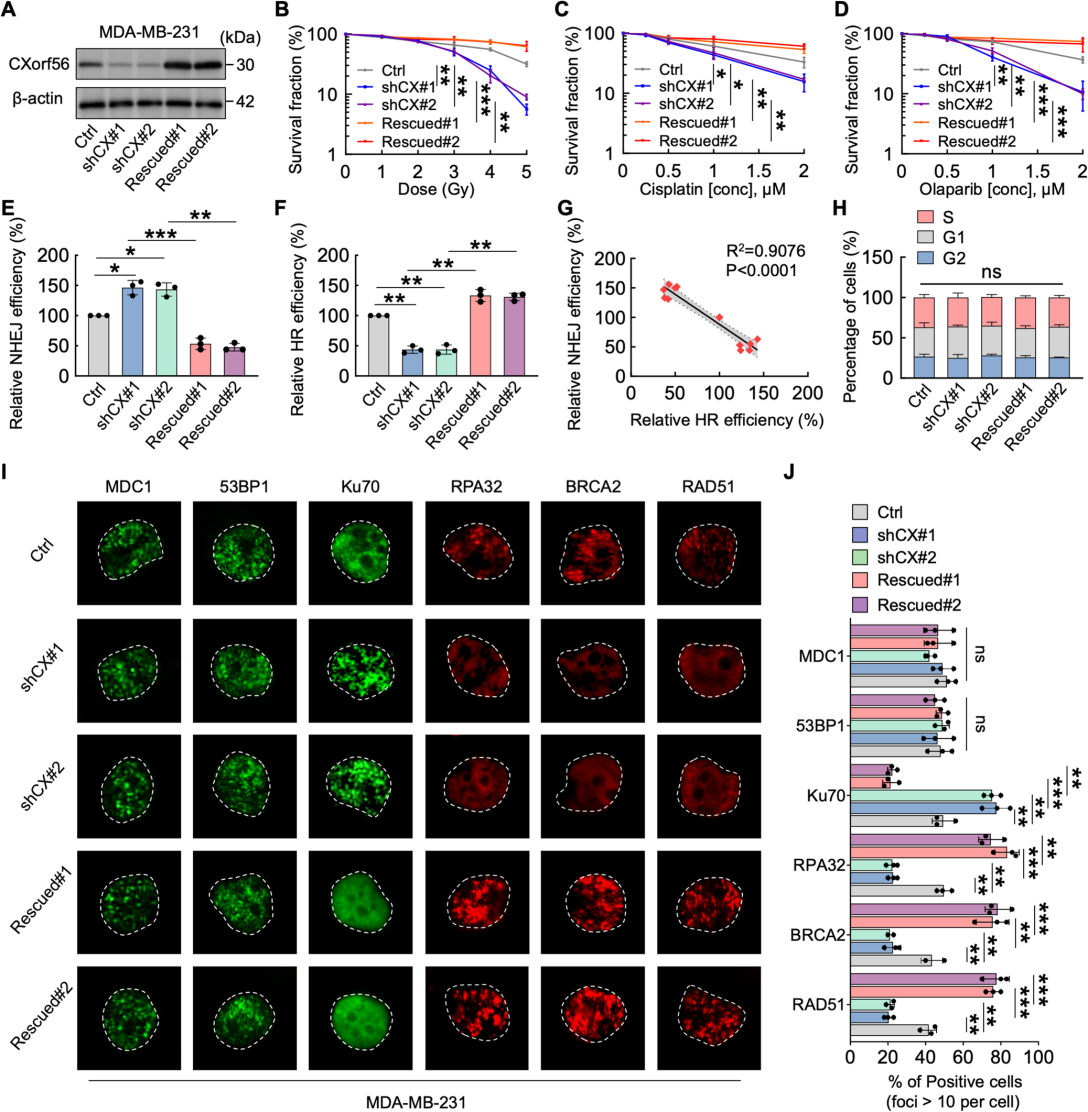
*CXorf56* protein inhibits NHEJ and promotes HR, increasing resistance to DNA-damaging agents. **A** Confirmation of *CXorf56* protein knockdown (shCX#1 and shCX#2) and re-expression (rescued#1 and rescued#2) in MDA-MB-231 cells. The sensitivity of control (Ctrl), *Cxorf56*-knockdown, or *CXorf56* re-expression MDA-MB-231 cells to IR (**B**), Cisplatin (**C**), and Olaparib (**D**) was assessed by colony formation assays. Data were analyzed by a two-tailed *t* test. **E**, **F** Ctrl, *CXorf56*-knockdown, or *CXorf56* re-expression MDA-MB-231 cells, were transfected with NHEJ reporter (EJ5-GFP) or HR reporter (DR-GFP) along with pCBA-I-SceI and mCherry. Forty-eight hours later, cells were harvested and subjected to flow cytometric analysis. Data were analyzed by a two-tailed *t* test. **G** Analysis shows linear regressions and Pearson correlations between relative NHEJ and HR efficiency in Ctrl, *CXorf56*-knockdown, and *CXorf56* re-expression MDA-MB-231 cells. Data were analyzed by a *F* test. **H** Cell cycle analyses of Ctrl, *CXorf56*-knockdown, or *CXorf56* re-expression MDA-MB-231 cells show that the change of *CXorf56* protein levels did not alter the cell cycle distribution of MDA-MB-231 cells. Data were analyzed by ANOVA and two-tailed *t* test. **I**, **J** Ctrl, *CXorf56*-knockdown, and *CXorf56* re-expression MDA-MB-231 cells were treated with IR (1 Gy, 1 hour for MDC1, 53BP1, Ku70; 1 Gy, 3 hours for RPA32; 1 Gy, 5 hours for BRAC2 and RAD51), and indicated foci were detected by immunofluorescence. Representative images are shown in **i**. Quantification of focus signals is shown in **J**. Data were analyzed by ANOVA and two-tailed *t* test. Remarks: ^ns^*p* ≥ 0.05, *p < 0.05, **p < 0.01, ***p < 0.001; Data are presented as mean ± SD. Scale bars = 10 μm.

Since MDA-MB-231 cells harbored the highest *CXorf56* protein expression and *CXorf56*-knockdown caused significant genome instability in these cells, we chose MDA-MB-231 cells to further confirm the role of *CXorf56* pretein in the DDR of TNBC cells. Since we found that *CXorf56*-knockdown renders TNBC cells more sensitive to IR, cisplatin, and olaparib treatment, we next sought to exclude the possible off-target effects of *CXorf56*-knockdown. Reintroducing *CXorf56* protein with shRNA-immune cDNA (Rescued#1 and Rescued#2) into *CXorf56*-knockdown cells reversed their vulnerability to DNA-damaging agents (Fig. 4A–D). Furthermore, NHEJ or HR reporter assays showed that *CXorf56* protein depletion increased NHEJ efficiency, whereas HR was compromised, which was reversed by *CXorf56* re-expression (Fig. 4E, F). In addition, using microhomology-mediated end joining (MMEJ) reporter assays, we showed that compared to the NTC group, *CXorf56*-knockdown or rescue did not affect MMEJ efficiency, however, POLQ (MMEJ marker) knockdown significantly suppressed MMEJ efficiency (Supplementary Fig. 4a), indicating that *CXorf56* protein did not regulate MMEJ repair in TNBC cells. Linear regression analysis showed a negative correlation between NHEJ and HR efficiency in the control, *CXorf56*-knockdown, and *CXorf56* re-expression cells (Fig. 4G), suggesting that *CXorf56* protein may regulate the choice of DNA repair pathway in TNBC cells. Importantly, the change in *CXorf56* protein levels did not affect cell cycle progression (Fig. 4H), suggesting that the alteration in NHEJ and HR efficiency by *CXorf56* protein was not an indirect effect of cell cycle change.

To identify the potential targets of *CXorf56* protein in DDR, we first examined the ability of the main DDR components to form foci after damage. Response to DSBs starts with ATM-directed MDC1 phosphorylation, which then amplifies DDR and recruits repair proteins involved in NHEJ (53BP1) and HR (BRCA1) respectively, to the chromatin surrounding DSBs^21^. *CXorf56* protein did not influence MDC1 and 53BP1 focus formation in response to DNA damage, but *CXorf56* protein downregulation resulted in a significantly elevated accumulation of Ku70 foci, which was reversed by *CXorf56* re-expression (Fig. 4I, J), indicating that *CXorf56* protein inhibited NHEJ in TNBC cells. In contrast, *CXorf56* protein downregulation significantly reduced the accumulation of RPA32, BRCA2, and RAD51 foci, which was also reversed by *CXorf56* re-expression (Fig. 4I, J), suggesting that *CXorf56* protein promotes HR in TNBC cells. Notably, BRCA1/*CXorf56* protein or ATM/*CXorf56* protein double knockdown cells were more sensitive to olaparib, when compared with BRCA1 or ATM single knockdown cells **(**Supplementary Fig. 4b–e), indicating that *CXorf56* protein knockdown has an additive or synergistic effect, with regard to PARPi response, when combined with BRCA/ATM deficiencies. Collectively, these results suggest that *CXorf56* protein inhibits NHEJ, promotes HR, and increases resistance to DNA-damaging agents.

### *CXorf56* protein interacts with Ku70 and suppresses Ku70-mediated NHEJ repair to enhance olaparib resistance

**Fig. 5:**
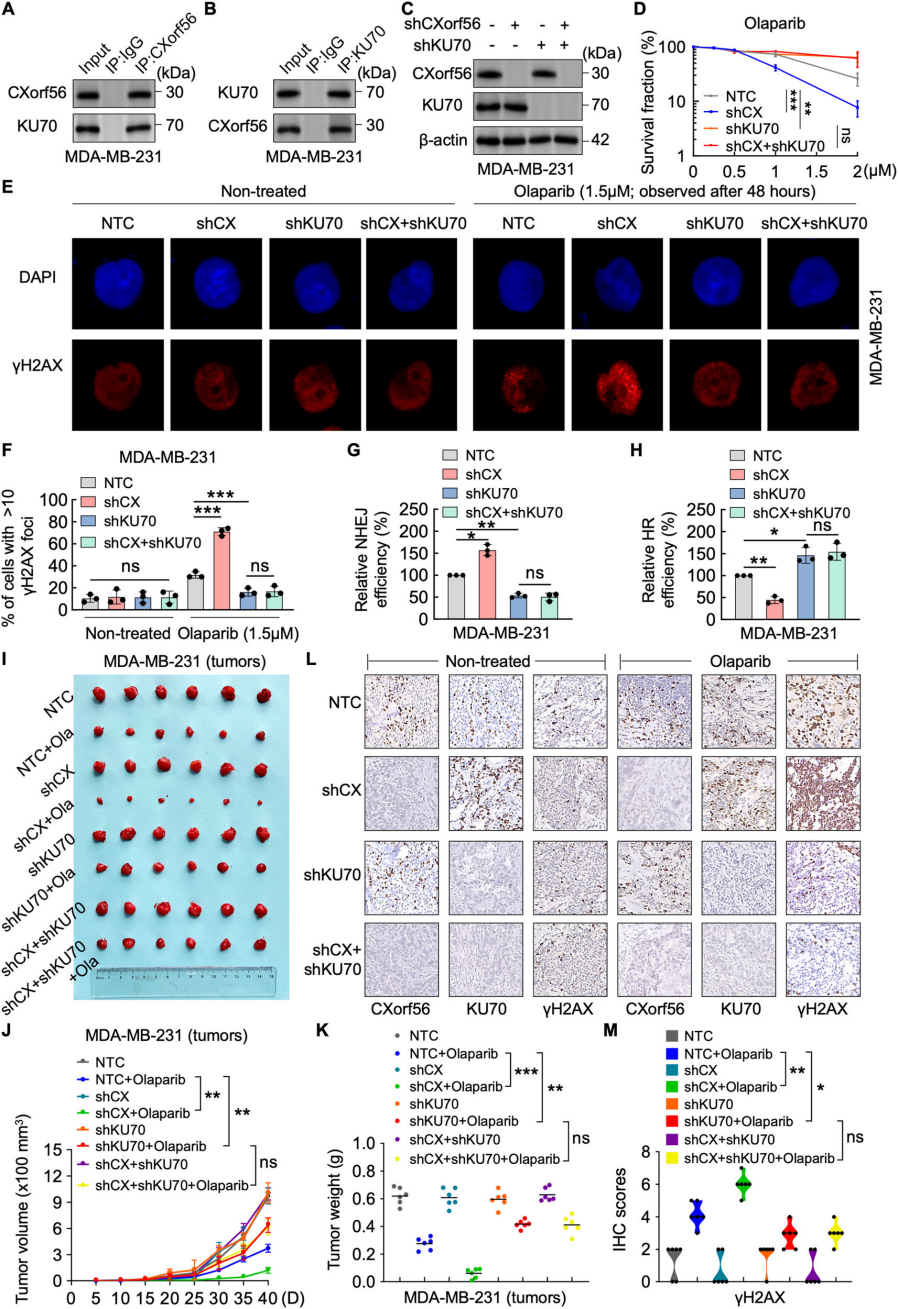
*CXorf56* protein interacts with Ku70, suppressing Ku70-mediated NHEJ repair to enhance Olaparib resistance. *CXorf56* protein (**A**) and Ku70 (**B**) complexes were co-immunoprecipitated with *CXorf56* protein and Ku70 antibodies and immunoblotted with the indicated antibodies. **C**, **D** MDA-MB-231 cells were infected with indicated lentiviral plasmids and treated with Olaparib. The indicated protein levels (**C**) and the sensitivity to Olaparib were assessed (**D**). Data were analyzed by a two-tailed *t* test. **E**, **F** NTC and corresponding knockdown MDA-MB-231 cells were treated with Olaparib (1.5 μM), and γ-H2AX foci before or 48 hours after Olaparib treatment were detected by immunofluorescence. Representative images of γ-H2AX foci are shown in **E**; Scale bars = 10 μm. Quantification of focus signals is shown in **F**. Data were analyzed by a two-tailed *t* test. **G**, **J** NTC and corresponding knockdown MDA-MB-231 cells were transfected with NHEJ reporter or HR reporter along with pCBA-I-SceI and mCherry. 48 hours later, cells were harvested and subjected to flow cytometric analysis. Data were analyzed by a two-tailed *t* test. **I**–**K** The tumor growth of the indicated MDA-MB-231 cells was examined in xenografts under the treatment of Olaparib (*n* = 6). Mouse xenograft tumors (**I**), the elevation of tumor size for 40 days (**J**), and the weights of the xenograft tumors (**K**) were presented. Data were analyzed by a two-tailed *t* test. **L** Representative IHC images of *CXorf56* protein, Ku70, and γ-H2AX expression in mouse xenograft tumor tissues; Scale bars = 100 μm. **M** Differences in the γ-H2AX protein levels in xenograft tumor tissues were presented in the violin plot (*n* = 6). Data were analyzed by a two-tailed *t* test.Remarks: ^ns^*p* ≥ 0.05, *p < 0.05, **p < 0.01, ***p < 0.001; Data are presented as mean ± SD.

Next, we sought to determine how *CXorf56* protein affects Ku70 focus formation in TNBC cells and whether this is related to DDR regulation. NHEJ and HR compete for repairing DSBs during the cell cycle, and Ku70 is the key protein in regulating the DNA repair pathway choice^22^. NHEJ begins with the recognition of DNA ends by the Ku70/80 heterodimer, and HR is subsequently inhibited^23^. Notably, *CXorf56* protein has been implicated in the Ku70 interactome^24^. These implications prompted validation of the relationship between *CXorf56* protein and Ku70 in regulating DDR. We found that *CXorf56* protein was present in the Ku70 complexes (Fig. 5A, B). Furthermore, *CXorf56* protein knockdown failed to suppress the increased resistance of Ku70-knockdown TNBC cells to olaparib (Fig. 5C, D). Additionally, the difference in γH2AX foci (8 h) between *CXorf56*-knockdown and negative control cells was eliminated by Ku70 knockdown (Fig. 5E, F). NHEJ and HR reporter assays also showed that *CXorf56* protein knockdown failed to reverse the decreased NHEJ efficiency and increased HR efficiency in Ku70-knockdown TNBC cells (Fig. 5G, H). We then subcutaneously implanted MDA-MB-231 cells into immunocompromised mice to further confirm the relationship between *CXorf56* protein and Ku70. Mice bearing *CXorf56*-knockdown MDA-MB-231 cells displayed more noticeable tumor shrinkage in the olaparib-treated group; this difference was eliminated with Ku70- knockdown (Fig. 5I–K). Further, changes in *CXorf56* protein or Ku70 levels did not alter cancer cell growth without drug intervention (Fig. 5I–K). Moreover, immunohistochemistry (IHC) analysis of tumor tissues confirmed that olaparib increased DNA damage in tumors with *CXorf56*-knockdown; the difference in γH2AX expression between *CXorf56*-knockdown and negative control cells was eliminated by Ku70 knockdown (Fig. 5L, M). These results suggest that *CXorf56* protein-mediated inhibition of NHEJ and promotion of HR by *CXorf56* protein are Ku70-dependent, and that *CXorf56* protein interacts with Ku70 to suppress Ku70-mediated NHEJ repair and enhances olaparib resistance.

### *CXorf56* protein binds to the Ku70 DNA-binding domain in S and G2 phases, impeding the recruitment of Ku70 and its downstream responders to DNA damage sites

**Fig. 6:**
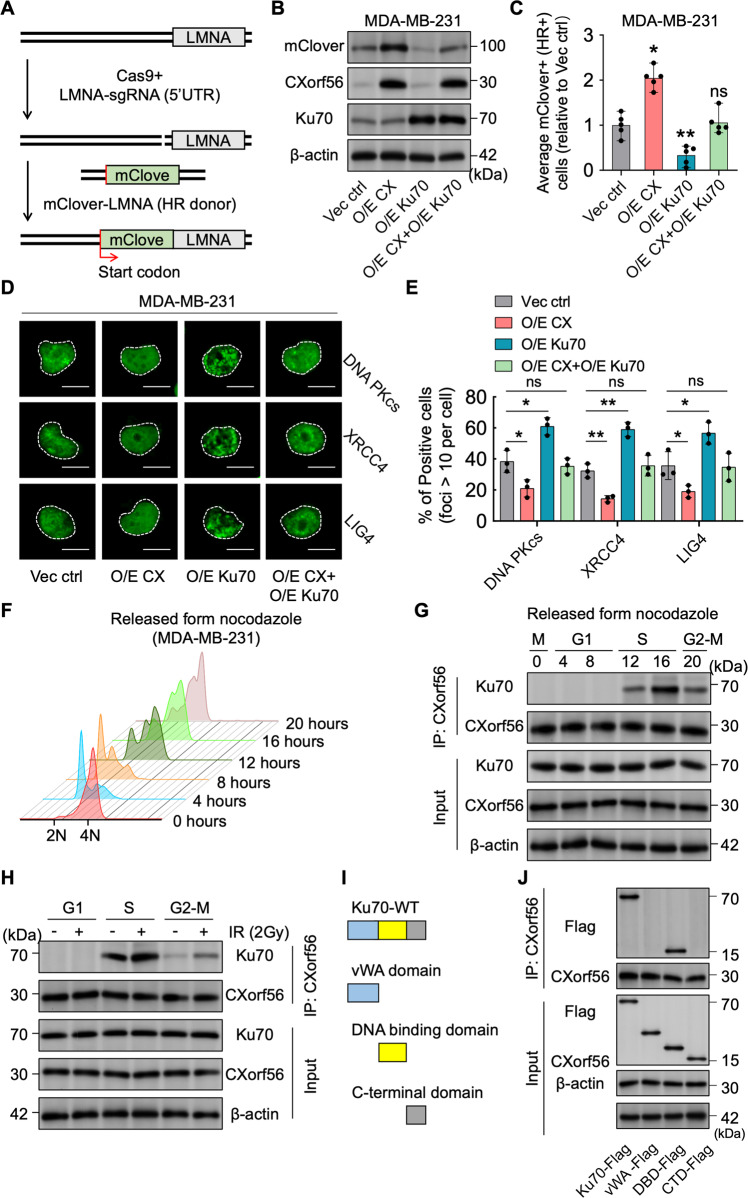
*CXorf56* protein binds to Ku70 DNA binding domain in S and G2 phases, impeding the recruitment of Ku70 and its downstream responders to DNA damage sites. **A** LMNA (lamin A) Cas9 reporter. CRISPR-Cas9 and sgRNA target the 5’ UTR of LMNA. Cas9 with sgRNA generates DSBs. HR fuses mClover with a start codon (red arrow) to LMNA for repair. mClover protein levels (**B**) were investigated by western blotting and the average percentage of mClover+ cells (**C**) in indicated MDA-MB-231 cells in five independent experiments, normalized to the vector control (Vec ctrl) group. Data were analyzed by a two-tailed *t* test. **D**, **E** Corresponding MDA-MB-231 cells were treated with IR (1 Gy, 1 hour for DNA-PKcs, XRCC4, and LIG4), and indicated foci were detected by immunofluorescence. Representative images are shown in **D**, Scale bars = 10 μm., and quantification of focus signals is shown in **E**. Data were analyzed by a two-tailed *t* test. **F**–**H** MDA-MB-231 cells were synchronized with nocodazole (100 ng/ml) for 12 hours and released into the cell cycle. At the indicated time points, cells were harvested for cell cycle (**F**) and co-immunoprecipitation and Western blotting analysis without IR treatment (**G**) or with 1-hour of IR treatment (2 Gy) (**H**). **I** Schematic of the domains of Ku70. There are three main domains in Ku70: the N-terminal vWA domain (blue), the core DNA binding domain (DBD, yellow), and the C-terminal domain (CTD, gray). **J** MDA-MB-231 cells were transfected with plasmids encoding the indicated Flag-tagged truncated Ku70 constructs. Cell lysates were subjected to immunoprecipitation with an anti-*Cxorf56* protein antibody. Precipitated proteins were analyzed by Western blotting with an anti-Flag antibody. Input controls were also included. Remarks: ^ns^*p* ≥ 0.05, *p < 0.05, **p < 0.01; Data are presented as mean ± SD.

**Fig. 7:**
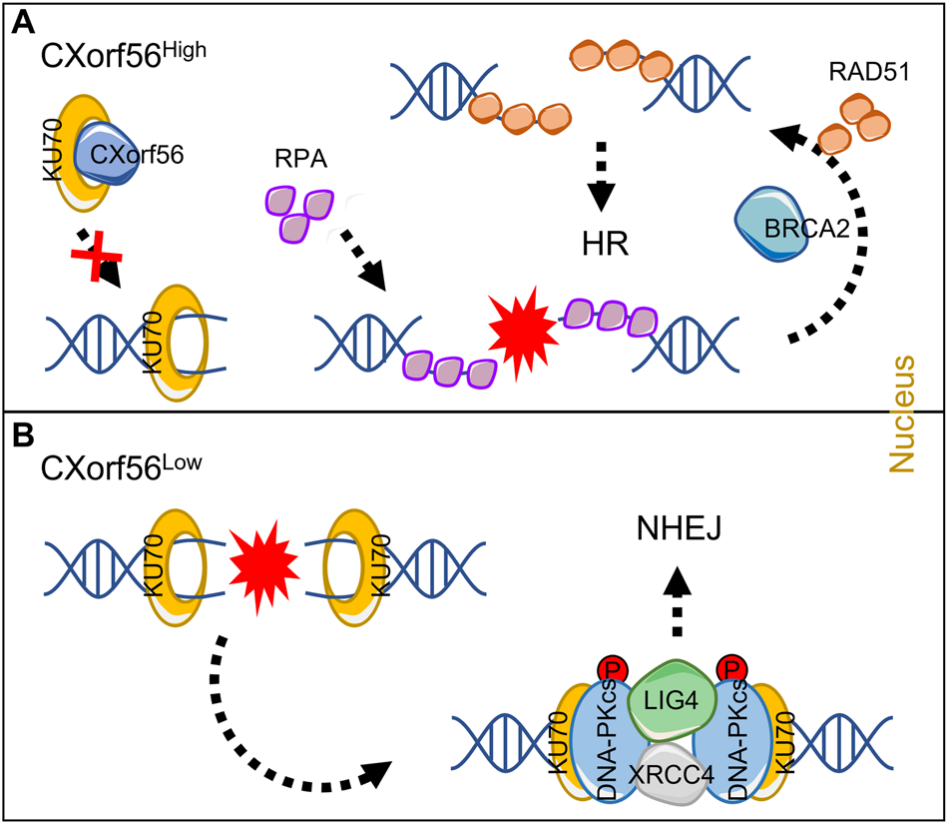
Proposed model for *CXorf56* protein in the regulation of DNA repair pathway choice. **A** Upregulated *CXorf56* protein naturally binds to Ku70, which blocks the binding of Ku70 with the damaged DNA, inducing a selection of HR over NHEJ by competitive recruitment of HR regulators for the repair of DSBs. **B** Without the binding of *CXorf56* protein with Ku70, Ku70 is recruited into chromatin at DSB sites and activates the NHEJ pathway by further forming the phospho-DNA-PKcs, XRCC4, and LIG4 complex.

To further investigate how *CXorf56* protein suppresses Ku70-mediated NHEJ to enhance HR, we generated DSBs using sgRNAs targeting the 5′-UTR of LMNA, providing the mClover–lamin A reporter as an HR donor for HR-mediated fusion of mClover to LMNA for repair (Fig. 6A). We found that *CXorf56* protein overexpression enhanced HR repair of DSBs, whereas Ku70 overexpression inhibited the HR repair of DSBs (Fig. 6B, C). Importantly, inhibition of HR repair by Ku70 overexpression was reversed by *CXorf56* protein overexpression (Fig. 6B, C). When DSBs occur, the heterodimer Ku70/80 recognizes DNA ends and recruits DNA-dependent protein kinase complexes (DNA-PKcs), further promoting the recruitment of XRCC4-LIG4 for NHEJ repair^25^. Therefore, we further investigated the recruitment of DNA-PKcs, XRCC4, and LIG4. Ku70 overexpression was found to promote the recruitment of DNA-PKcs, XRCC4, and LIG4, which was reversed by *CXorf56* protein overexpression (Fig. 6D, E). In addition, we found that compared to the NTC group, recruitment of phospho-DNA-PKcs (Ser2056 and Thr2609) to sites of DNA damage was increased in *CXorf56* knockdown cells (Supplementary Fig. 5a, b). As *CXorf56* protein promotes HR by inhibiting Ku70-mediated NHEJ through *CXorf56*-Ku70 interaction, and because HR is inhibited during the G1 phase of the cell cycle, although both pathways are active in the S and G2 phases, we investigated whether the *CXorf56*-Ku70 interaction occurs in a cell cycle-dependent manner using MDA-MB-231 cells synchronized by release from nocodazole treatment (Fig. 6F). In these cells, *CXorf56* and Ku70 protein expression levels did not vary during the cell cycle (Fig. 6G). However, *CXorf56* protein predominantly interacted with Ku70 in the S- and G2- phase cells (Fig. 6G), and this interaction was enhanced under DNA damage (Fig. 6H). Interestingly, *CXorf56* protein did not promote recruitment to sites of IR-induced DNA damage during the TNBC cell S-phase (PCNA positively marks S-phase cells), however, KU70 recruitment to sites of DNA damage was increased during the TNBC cell S-phase after *CXorf56* knockdown (Supplementary Fig. 5c). Moreover, there are three main domains in the Ku70 protein: the N-terminal vWA domain, core DNA binding domain, and divergent C-terminal domain (Fig. 6I)^26^. To further identify the Ku70 domain bound to *CXorf56* protein, FLAG-tagged Ku70 and its truncated constructs were ectopically expressed in MDA-MB-231 cells. *CXorf56* protein directly bound to the DNA-binding domain of Ku70, rather than the other domains (Fig. 6J). Collectively, these results indicate that *CXorf56* protein indirectly suppresses NHEJ and promotes HR repair during the S/G2 phase by interacting with the DNA-binding domain of Ku70 and suppressing the recruitment of KU70 to sites of DNA damage. Therefore, we propose that *CXorf56* protein is a cell cycle-dependent inhibitor of Ku70-mediated NHEJ that promotes error-free repair by HR in breast cancer (Fig. 7).

## Discussion

Several treatment modalities, such as chemotherapy, radiation, surgery, endocrine therapy, targeted therapy or combination therapy, are used to manage breast cancer. The type of treatment used depends on tumor stage and biology. Breast cancer care has been shown to improve OS, DFS and reduce morbidity and mortality^21^. The number of cancer survivors continues to increase due to the advances in early diagnosis and treatment^22^. Thus, the OS rates of patients with breast cancer largely depends on the therapy response rate. Numerous therapeutic strategies are based inducing cancer cell cytotoxicity, and the cancer cells with a higher DNA repair capacity will result in a lower therapy response rate. TNBCs are characterized by high levels of chromosomal instability, and therapies that leverage DNA repair defects have demonstrated varying degrees of success^27^. Recently, PARPis have emerged as promising therapies for breast cancer. In the OlympiAD Study and OlympiA Study, PARPi significantly reduced the risk of disease recurrence and progression compared with chemotherapy in early-stage and metastatic breast cancer with *BRCA1/2* mutations^5,6^. The St. Gallen consensus, American Society of Clinical Oncology, and National Comprehensive Cancer Network support the therapeutic status of PARPi, particularly for TNBC. In addition, non-cancerous cells replicate slower than cancer cells, lack *BRCA1* mutations and maintain homologous repair capabilities, which allows them to survive in the presence of PARP inhibitors. Therefore, limiting the side effects associated with PARPi treatment, which has great advantages in cancer treatment.

There are two main factors that limit the application of PARPis in breast cancer treatment. First, the low incidence of *BRCA1/2* mutations in breast cancer leads to limited access to PARPis; second, breast cancer cells, particularly TNBC cells, have been observed to rapidly develop resistance to PARPis through multiple pathways after treatment^14^. For example, in *BRCA1*-deficient TNBC cells, downregulation of EMI1 leads to RAD51 accumulation, which restores HR^28^. Further, RAD52 enables RAD51 to gain access to resected DNA ends in the absence of *BRCA1/2*^29,30^, suggesting that *BRCA*-deficiency is insufficient for stable HRD induction owing to the existence of backup HR pathways. Non-TNBC patients with BRCA1/2 mutations seldom develop resistance to PARPi^31^. Therefore, there is a need to evade PARPi resistance and develop potential treatment strategies for TNBC. Therefore, additional targets must be identified to induce a stable HRD status with “*BRCA* mutation-like effects” to sensitize cancer cells to DNA-damaging therapies and expand the addressable population of TNBC patients for PARPis. Moreover, NHEJ plays a critical role in the hypersensitivity of HR-deficient cells to PARPis. Ablation of 53BP1, NHEJ-mediated DSB repair regulator, rescues the genotoxicity of DNA-damaging agents in *BRCA1*- or ATM-deficient TNBC cells^32,33^, suggesting that unrestricted NHEJ can induce genomic instability and eventual lethality in HR-deficient TNBC cells. Therefore, we screened potential candidates from a gene set associated with DDR and clinical prognosis in patients with breast cancer, based on consistent suppression of the HR/NHEJ ratio in TNBC cells upon siRNA knockdown. We found that *CXorf56* protein functions as a novel DDR regulator, whose knockdown increased genomic instability and NHEJ, suppressed HR, and increased the sensitivity of TNBC cells to olaparib in vitro and in vivo, suggesting that combined treatment with *CXorf56* protein depletion and PARPis may be a potential novel strategy to treat the TNBC with *CXorf56* protein expression.

NHEJ and HR compete for DSB repair during the cell cycle^34^, and the choice between HR and NHEJ depends primarily on the cell cycle stage^35^. Considering that *CXorf56* protein simultaneously inhibits NHEJ and enhances HR, but does not alter the cell cycle, NHEJ and HR markers were investigated upon altering *CXorf56* protein levels in TNBC cells. We found that *CXorf56* protein suppressed recruitment of the NHEJ marker Ku70 and promoted the recruitment of HR markers, including RPA32, BRCA2, and RAD51. The Ku heterodimer (Ku70/Ku80) is the main component of the NHEJ pathway that repairs DSBs. Ku70 depletion induces PARPi resistance in *BRCA1-*mutant cells by restoring HR-mediated repair of DSBs^36^, suggesting that Ku70 is a key protein in regulating the DNA repair pathway choice between NHEJ and HR. Ku70 binds directly to exposed DNA ends, triggering NHEJ during the S and G2 phases, and reduces HR repair^36^. Notably, previous evidence^24^ and our results revealed that *CXorf56* protein interacts with Ku70 in TNBC cells, which suggests need for further investigation of whether this interaction of *CXorf56* protein with Ku70 suppresses Ku70-mediated NHEJ repair in response to DNA damage. The core NHEJ machinery encompasses Ku70/Ku80 heterodimer-mediated recruitment of DNA-PKc and LIG4/XRCC4^37^. Furthermore, *CXorf56* protein overexpression suppressed the recruitment of phospho-DNA-PKcs, XRCC4, and LIG4, indicating that *CXorf56*-Ku70 binding impedes the recruitment of downstream responders of Ku70 to DNA damage sites, forcing cancer cells to choose HR rather than NHEJ for DSB repair. During the G1 phase, HR is inactivated and NHEJ is dominant, however, during the S and G2 phases, when sister chromatids are available, NHEJ and HR compete^38^. Although HR inhibition during G1 is well understood, it is unclear why the abundant NHEJ machinery does not always outcompete HR during S and G2, suggesting that an active NHEJ suppressor mechanism operates during and after replication. Further, we observed that the interaction of *CXorf56* protein with Ku70 was predominant in the S- and G2-phase cells, which was enhanced under DNA damage conditions, suggesting that *CXorf56* protein is a cell-cycle-dependent negative regulator of NHEJ that promotes error-free repair by HR in the S and G2 phases.

Herein, the *CXorf56*-Ku70-HR repair axis in breast cancer was identified. HR-based DNA repair affects the clinical outcomes of cancer treatment and drug resistance^39,40^. We found that *CXorf56* transcription levels are significantly upregulated in breast cancer tissues, suggesting that *CXorf56* plays a key role in the development of breast cancer and can be utilized as a specific therapeutic target for breast cancer. Our cohort study revealed that *CXorf56* protein is an independent predictor of survival in patients with breast cancer, underscoring the potentially crucial role of *CXorf56*-mediated HR proficiency in their poor prognosis. We also found that the mRNA and protein expression of *CXorf56* was significantly upregulated in TNBC tissues compared with that in non-TNBC tissues. With this unique feature (PARPi resistance) in TNBC, we assumed that *CXorf56* protein should be certain specific markers responsible for the enhancement of HR ability against PARPi cytotoxicity in TNBC when compared with the non-TNBC. To the best of our knowledge, this is the first study that focuses on the role of *CXorf56* protein in cancers. In addition to its therapeutic value, the diagnostic and prognostic value of *CXorf56* protein in breast cancer were also elucidated.

This study also has certain limitations. Presently, PARPis can only be used off-label in patients with *BRCA*-mutation TNBC in China. Therefore, it is very difficult to collect tissue specimens from patients with PARPi resistance and sensitivity. In our future study, we plan to construct PARPi-resistant cell lines, and will continue to focus on the clinical application of olaparib. In addition, sequencing of tissue samples from patients with PARPi resistance and sensitivity will also be performed to further verify the regulatory role of *CXorf56* on olaparib resistance. This will assist in the discovery of other PARPi resistance-related molecules and new resistance mechanisms. Moreover, determining the relationship between the *BRCA1/2* mutation and *CXorf56* could serve as an indicator to improve the prediction response to PARPis. Since genetic testing is not routine, we did not investigate the relationship between the *BRCA1/2* mutation and *CXorf56* in this study. However, routine genetic testing needs to be considered in the future.

Taken together, we demonstrate that *CXorf56* protein is a binding partner of Ku70, which negatively inhibits Ku70-mediated NHEJ to guide TNBC cells toward HR repair for DSBs in the S and G2 phases. Future studies could prepare and utilize gene inhibitors for TNBC by inhibition of *CXorf56* protein. Thus, targeting the *CXorf56*-Ku70 pathway in TNBC in combination with PARPis can help overcome drug resistance and be used for the treatment of non-*BRCA* mutation TNBC.

## Methods

### Cell lines and cell culture

Breast cancer cell lines were obtained from the American Type Culture Collection (http://www.atcc.org). Breast cancer cell lines are included ZR751 (catalog number CRL-1500, ATCC), MCF-7 (catalog number CRL-3435, ATCC), T47D (catalog number HTB-133, ATCC), BT549 (catalog number HTB-122, ATCC) and MDA-MB-231 (catalog number CRM-HTB-26, ATCC). Cell lines were authenticated twice using morphological and isoenzyme analyses. The cells were cultured in Dulbecco’s modified Eagle’s medium (Invitrogen, Waltham, MA, USA) containing 10% fetal bovine serum (Invitrogen) in a humidified 5% CO_2_ incubator at 37.5 °C. For stable cell line establishment with the indicated gene knockdown and overexpression, specific constructs were transfected into cancer cells using a lentiviral infection system followed by selection with blasticidin (5 μg/mL) or puromycin (3 μg/mL).

### Constructs

Specific shRNAs targeting *CXorf56* and *Ku70* were respectively cloned into a pLKO.1-puro vector (Addgene, Watertown, MA, USA) for gene knockdown; the NTC sequences are listed in the Supplementary Information. For stable overexpression of the indicated proteins, the coding sequence (CDS) for each gene was cloned into the pLenti-CMV-blast vector (Addgene); the primers used to amplify full-length CDS of *CXorf56* and *Ku70*, and the corresponding truncated CDS of *Ku70* together with the FLAG-tag sequence are listed in the Supplementary Information. As the shRNAs of *CXorf56* do not target the CDS, the *CXorf56* full-length construct was used for *CXorf56* re-expression in *CXorf56*-knockdown cells. The pCas9-sgLMNA plasmid (#98971) cloned into the pX330 backbone and mClover-LMNA donor plasmid (#122508) were obtained from Addgene; the sgRNA-targeting sequence is listed in Supplementary information.

### siRNA screening using the SeeSaw 2.0 Reporter assay

In the SSR 2.0 system, two I-SceI target sites with opposite orientations were cloned at the 3′-end of the green fluorescent protein (GFP) gene. I-SceI expression induces DSBs; when damage is repaired by NHEJ or HR, cells express GFP or red fluorescent protein (RFP), respectively^41^. In this study, cells were transfected with SeeSaw 2.0, according to the manufacturer’s protocol, and selected for 2 weeks using G418. Subsequently, cells were infected with lentivirus expressing I-SceI, seeded into 96-wells plates (2000 cells/well), followed by transfection with the indicated siRNAs. The siRNAs and sequences of the NTC for the indicated gene silencing are listed in the Supplementary Information. After 36 h, the cells were harvested and fixed with 4% paraformaldehyde (PFA) for 20 min at room temperature, rinsed with 1× phosphate-buffered saline (PBS), and subjected to flow cytometry.

RFP+ (HR- predominant) and GFP+ (NHEJ- predominant) cells in the NTC and each si-RNA group were detected using flow cytometry. Then, the HR/NHEJ ratio was calculated, and the fold change was determined (siRNA versus. NTC). Using the fold change values, z-scores were computed and combined (the scores for individual cell lines, TNBC and luminal (*n* = 3, each), were averaged) into one composite score. A positive z-score value represents the HR/NHEJ fold change decrease with siRNA knockdown, and indicates that the candidate is important for HR repair. Three average TNBC and luminal z-scores were compared using the *T*-test, and the *p*-value was calculated accordingly. Also, ∆z-scores were calculated by subtracting the average z-scores in the luminal group from the average z-scores in the TNBC group in each trial. A negative ∆z-score indicated that the candidate is important for the TNBC cells. z-score standardisation for siRNA screening has been reported previously^42^.

### Western blotting and immunoprecipitation

Cells were lysed with NETN buffer (0.1 mM EDTA, 10 mM NaF, 20 mM Tris-HCl [pH 8.0], 100 mM NaCl, and 0.5% NP-40 with protease inhibitors) for 30 min before centrifugation. Supernatants were incubated overnight at 4 °C with *CXorf56* protein antibody (catalog number 24021-1-AP, Proteintech, Rosemont, IL, USA), followed by immunoprecipitation (6 h, 4 °C) with agarose beads (Amersham Biosciences, Amersham, UK). For western blotting, protein samples were separated by 10% sodium dodecyl sulfate-polyacrylamide gel electrophoresis and then transferred to PVDF membranes, which were incubated with primary antibodies (overnight, 4 °C), followed by incubation with corresponding secondary antibodies at room temperature. The primary antibodies used were *CXorf56* protein (catalog number 24021-1-AP, Proteintech, 1:1000), Ku70 (catalog number ab92450, Abcam, Cambridge, UK, 1:1000), FLAG (catalog number ab205606, Abcam, 1:5000), and β-actin (catalog number sc-81178, Santa Cruz Biotechnology, Dallas, TX, USA, 1:10000).

### Quantitative real-time PCR (qPCR)

Total mRNA was extracted using RNAiso Plus reagent (Takara, Kusatsu, Japan). qPCR was performed using Power SYBR Green PCR Master Mix (Takara). Gene expression was quantified based on the 2^−ΔΔCT^ value, normalized to GAPDH. The primers used for qPCR are shown in the Supplementary information.

### Colony formation analysis

Briefly, 1000 cells were seeded in six-well plates overnight and then treated. After 12–14 days, colonies were fixed with methanol, stained with 0.1% Giemsa, and quantified. The survival fraction was calculated by dividing the number of colonies in each treated group by the number of colonies in the non-treated group. The difference in the treatment responsiveness was compared using the unpaired two-tailed Student’s *t*-test in each group, using the largest treatment dose (IR: 5 Gy, cisplatin: 2 μM, and olaparib: 2 μM).

### Tissue samples and immunohistochemistry

Archived formalin-fixed, paraffin-embedded samples obtained from 180 patients with breast cancer at Jiangsu Cancer Hospital were used for immunohistochemistry staining. The details of the cohorts that were compared are provided in the Supplementary Table 1. All patients provided signed written informed consent, and the study was approved by the Ethics Committee of the Jiangsu Cancer Hospital Authority (approval number 2022-014). The Institutional Review Board (IRB) approval details and assurance of informed consent are provided in the supplementary materials. IHC staining was performed on tissue sections using *CXorf56* protein (catalog number PA5-58310, Thermo Fisher Scientific, Waltham, MA, USA, 1:100), ER (catalog number PA1-311, Thermo Fisher Scientific, 1:500), PR (catalog number MA1-411, Thermo Fisher Scientific, 1:500), Her2 (catalog number MA5-13105, Thermo Fisher Scientific, 1:500), γ-H2AX (catalog number ab229914, Abcam, 1:200), and Ku70 (catalog number ab92450, Abcam, 1:100) antibodies, followed by a streptavidin-biotin labeling protocol (Dako, Carpinteria, CA, USA). *CXorf56* protein and γ-H2AX expression scores were assessed based on the semiquantitative German scoring system as previously described^43^, which considers the extent of cell staining (≤10% positive cells for 1; 11–50% positive cells for 2; 51–80% positive cells for 3; >80% positive cells for 4) and staining intensity (slight staining for 1; moderate staining for 2; strong staining for 3). The scores for percentage of positive cells and staining intensity were added (slices with no staining were scored 0). Therefore, the IHC scores were either 0, 2, 3, 4, 5, 6, or 7. The *CXorf56* protein scores of 0, 2, and 3 were associated with low *CXorf56* protein expression, while the scores of 4, 5, 6, and 7 were as associated with high *CXorf56* protein expression.

### Flow cytometry

For NHEJ or HR reporter assays, 2 × 10^5^ cells were transfected with an HR reporter (DR-GFP) or NHEJ reporter (EJ5-GFP) along with pCBA-I-SceI and mCherry. After 48 h, cells were trypsinized and resuspended in 25 mM HEPES (pH 7.0, 1% (v/v) fetal bovine serum, 2 mM EDTA, and 1× PBS) and subjected to flow cytometric (Attune NxT Flow cytometer; Thermo Fisher Scientific). For Cas9-LMNA reporter assays, 2 × 10^5^ cells were seeded in a 6-well plate and transfected after 24 h using Lipofectamine-3000 with mClover-LMNA HR donor plasmid (4 μg), and 2.5 μg of Cas9-expressing plasmid and a pair of gRNAs. At 72 h after transfection, cells were analyzed using flow cytometry. For cell cycle assays, cells were dissociated and then fixed in 70% cooled ethanol (overnight, −20 °C), and then incubated with propidium iodide supplemented with RNase for 30 min at room temperature. Flow cytometry and FlowJo software were used to analyze the cell cycle.

### Immunofluorescence

Cells were cultured on coverslips for 24 h before the experiments. For γ-H2AX, MDC1, 53BP1, Ku70, DNA-PKcs, LIG4, XRCC4, and BRCA2 foci, cells were fixed with 4% PFA, and permeabilized with 0.5% Triton X-100; for RAD51 foci, cells were permeabilized with 0.5% Triton X-100 on ice for 5 min and then fixed with 4% PFA. Cells were fixed and permeabilized with methanol: acetone (1:1) at −20 °C for 20 min to detect RPA32 foci. Cells were incubated with primary antibodies (4 °C, overnight) and subsequently incubated with the corresponding Alexa Fluor 488- or 594-conjugated secondary antibodies (37 °C, 20 min), and nuclei were stained with DAPI. Coverslips were then mounted on glass slides using an anti-fade solution and visualized (Eclipse 80i fluorescence microscope; Nikon, Tokyo, Japan) and related software. Foci quantification was performed using ImageJ software v1.8.0 (National Institutes of Health, Bethesda, MD, USA).

### Tumor xenograft

Female BALB/C nude mice (5 weeks, about 20 g) were purchased from Beijing Vital River Laboratory Animal Technology Co., LTD (Beijing, China). Mice were housed in a pathogen-free animal facility at 22 ± 2 °C under controlled 12-h-light-12-h-dark cycles,with humidity at 55 ± 10%. Mice were given regular diets and had access to autoclaved water ad libitum. Animal experiments were performed with the approval of Nanjing Medical University at the Animal Core Facility of Nanjing Medical University and conform to all relevant regulatory standards. Animals were divided into groups by simple randomization through a random number table. MDA-MB-231 cells were injected subcutaneously into the flanks of 5-week-old female BALB/C nude mice. Each mouse was injected with 100 μL of 2 × 10^6^ cells in PBS with growth factor-reduced Matrigel (2:1) (BD Biosciences, Franklin Lakes, NJ, USA). Mice were randomly divided into the vehicle- (10% DMSO with 10% 2-hydroxypropyl-β-cyclodextrin daily) or Olaparib-treated (50 mg/kg every three days) groups. Subsequently, tumor volume was measured every 5 days using calipers and was calculated as length × width^2^. On day 40, all mice were given euthanasia through amobarbital injection of three times standard doses (150 mg/kg), and the tumor were isolated, measured the volume (using the two-tailed unpaired Student’s *t*-test to compare the difference), fixed, and sectioned for IHC staining.

### Cell synchronization

For synchronization, MDA-MB-231 cells were treated with nocodazole (100 ng/mL) for 12 h and then cultured in normal medium. At the indicated time points after treatment, cells were harvested for cell cycle profiling, western blotting, and immunoprecipitation analyses.

### Pulsed-field gel electrophoresis (PFGE)

Flasks of 25 cm^2^ were inoculated with 10^6^ MDA-MB-231 cells in each group 48 hours prior to treatment with IR. In the case of homogeneous DNA labeling, cells were incubated with [14 C]-thymidine (0.5 mM) in media for 24 hours before treatment. This was followed by irradiation with IR (1 Gy, 1 hour). Cells were harvested immediately after irradiation in 37°C, 5% CO_2_. Agarose plugs were prepared as previously described^44^. Quantification was done by ImageJ. Three individual experiments were performed for each setup.

### Microhomology-mediated end joining (MMEJ) assay

The EGFP-MMEJ reporter was described previously^45^. To induce DSBs in cells, MMEJ reporter cells were transfected with Cas9-WT/gRNA plasmids (#44250, Addgene). EGFP-positive events were scored by FACS analysis 5 days later. FACS analysis was performed using a BD Accuri C6 flow cytometer and accompanying data analysis software (FlowJo).

### Statistics and reproducibility

Level-3 RNA sequencing data were downloaded from TCGA (https://portal.gdc.cancer.gov/). The TCGA-BRCA datasets comprised 1068 tumors. The Bioconductor package “edgeR” was used for gene expression calculations. OS-related genes were screened and verified using the GEPIA2 database as described previously^44^. The top 500 OS-related breast cancer genes were obtained by searching the ‘Most Differential Survival Genes’ section of the GEPIA2 website using the following terms: BC (cancer name), OS (method) and median for the expression cut-off for splitting high- and low-expression cohorts, and were ranked according to the *p*-values obtained from Kaplan–Meier analysis, with *p*-value less than 0.05 indicating statistical significance. A Student’s bilateral *t*-test was used for comparison between the two groups. Survival analyses were performed using Kaplan–Meier plots and log-rank tests. Cox proportional hazards model was used to identify independent predictors associated with the OS in patients with breast cancer. Statistical significance was set at *P* < 0.05.

### Reporting summary

Further information on research design is available in the Nature Research Reporting Summary linked to this article.

## Supplementary information

Supplementary Information

Reporting Summary

Supplementary Dataset 1

## Data Availability

The data generated in this study are available upon request from the corresponding author.
