# Supplementary material for: Inhibition of CXorf56 promotes PARP inhibitor-induced cytotoxicity in triple-negative breast cancer
Source: NPJ Breast Cancer. 2023 May 8;9:34. doi: 10.1038/s41523-023-00540-3 (PMC10167262; doi:10.1038/s41523-023-00540-3)
Supplement: Supplementary file 1 — Supplementary Information [file 41523_2023_540_MOESM1_ESM.pdf]

Supplementary information

Supplementary figures

Supplementary Figure 1

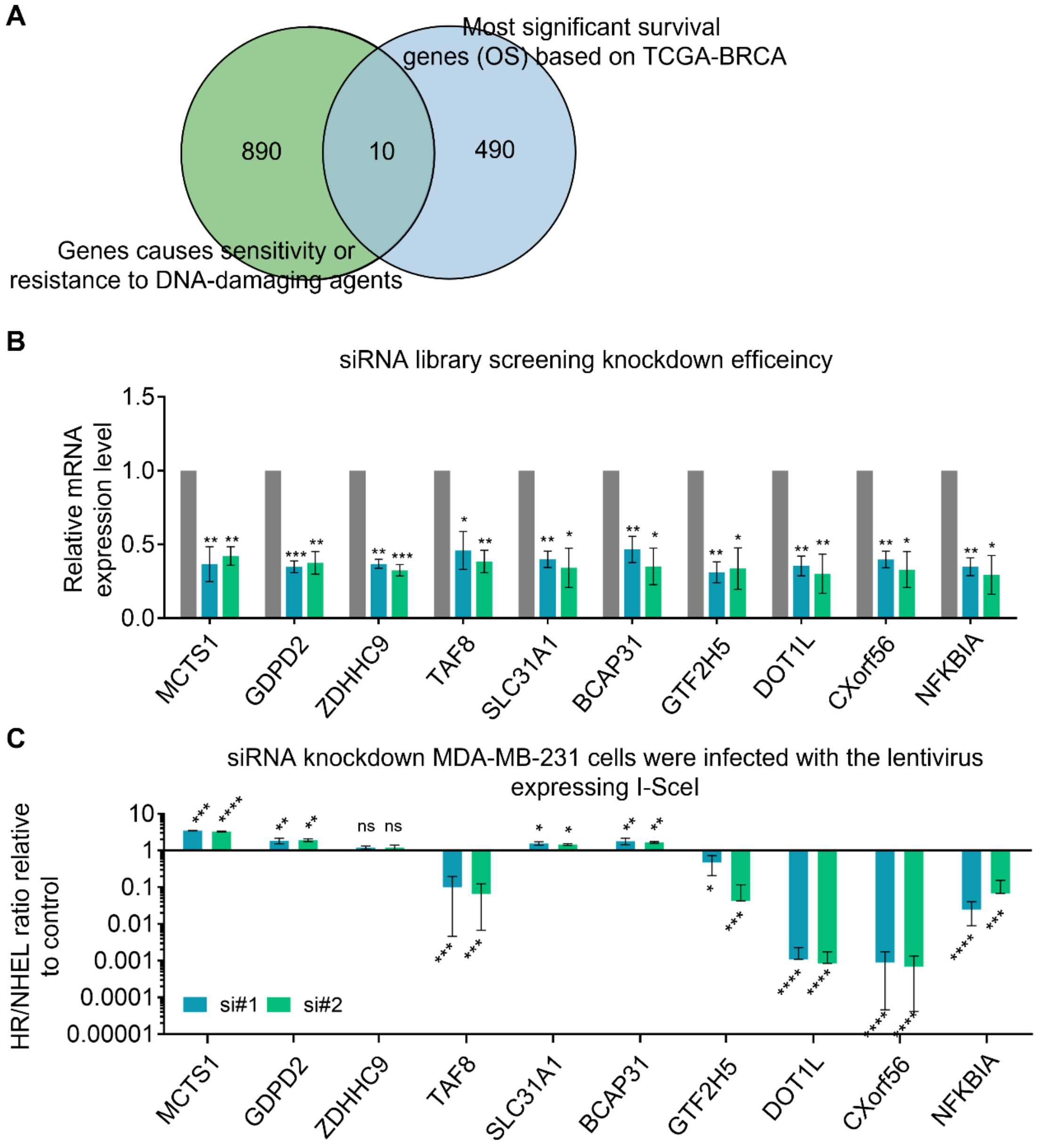

Supplementary Figure 1. Related to Figure 1. (A) Venn diagram for the overlap among the most significant overall survival (OS)-related genes and DNA damage response (DDR)-related genes. **(B)** The qPCR determined the knockdown efficiency of

each gene in MDA-MB-231 cells upon siRNA library screening. Data were analyzed by a two-tailed t test. **(C)** MDA-MB-231 cells expressing the SeeSaw 2.0 reporter system were seeded in 96-well plates containing the siRNA library (5 nM pooled siRNA for each gene) for 72 hours. Then, cells were infected with the lentivirus expressing I-SceI for 48 hours, and the RFP/GFP ratio was measured by flow cytometry to represent each group's HR/NHEJ ratio. Those siRNAs with an average normalized RFP: GFP ratio below 1 were included in the category of gene knockdown that decreases HR. In contrast, the RFP: GFP ratio above 1 means gene knockdown favors HR. Data were analyzed by a two-tailed t test. Remarks: $^{ns}p \geq 0.05$, $*p < 0.05$, $**p < 0.01$, $***p < 0.001$, $****P < 0.0001$. Data are presented as mean ± SD.

Supplementary Figure 2

A

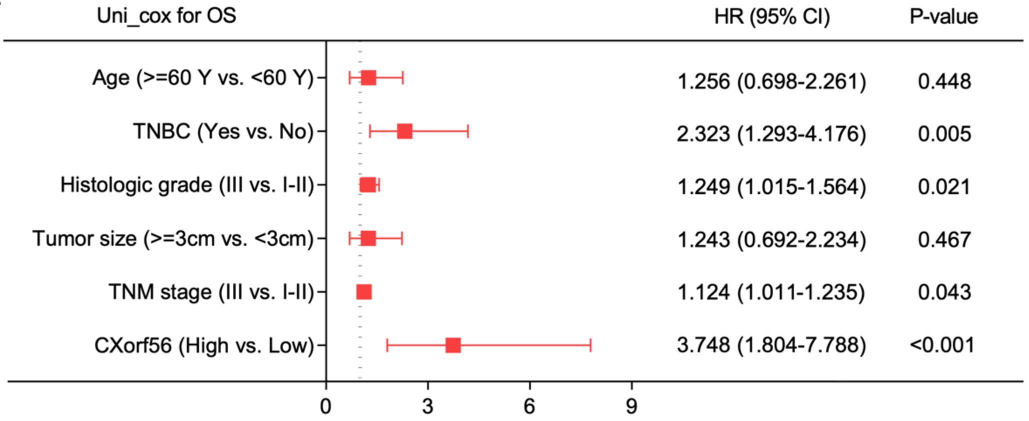

B

Correlation analyses between TNBC and clinicopathological features

Clinical features	Groups	Total number of cases	TNBC		X^2	P-value*
			No	Yes		
Histologic grade	I-II	99	76	23	4.798	0.028
	III-IV	81	50	31		
TNM stage	I-II	111	84	27	4.442	0.035
	III-IV	69	42	27		

Supplementary Figure 2. Related to Figure 2. (A) The prognostic factors for OS of BC patients were figured out based on the univariate analysis, which is shown in the forest plot. Data were analyzed by a Wald test. **(B)** Correlations between TNBC and clinicopathological features in 180 breast cancer cases from the in-house cohort study. Data were analyzed by a chi square test.

Supplementary Figure 3

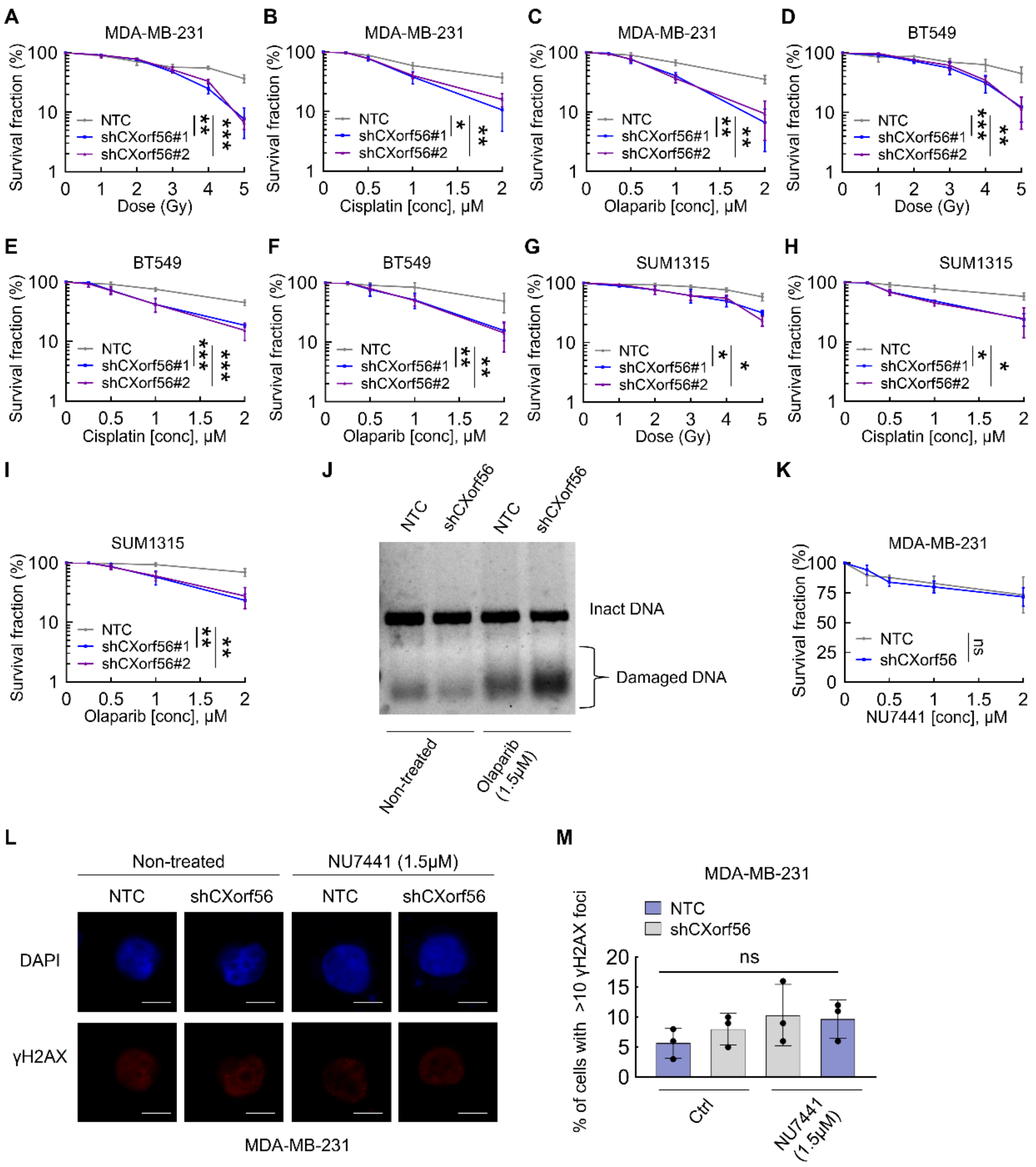

Supplementary Figure 3. Related to Figure 3. (A-C) The sensitivity of non-target control (NTC) and CXorf56 knockdown MDA-MB-231 cells to IR (A), Cisplatin (B), and olaparib (C) was assessed by colony formation assays. Data were analyzed by a two-tailed t test. **(D-F)** The sensitivity of non-target control (NTC) and CXorf56 knockdown BT549 cells to IR (D), Cisplatin (E), and Olaparib (F) was assessed by

colony formation assays. Data were analyzed by a two-tailed t test. **(G-I)** The sensitivity of non-target control (NTC) and CXorf56 knockdown SUM1315 cells to IR (G), Cisplatin (H), and Olaparib (I) was assessed by colony formation assays. Data were analyzed by a two-tailed t test. **(J)** Detection of DSB formation by PFGE in NTC and CXorf56 knockdown MDA-MB-231 cells without or with olaparib treatment. Autoradiography of 0.5-h 14C-TdR-labelled DNA. **(K)** The sensitivity of NTC and CXorf56 knockdown MDA-MB-231 cells to DNA-PKcs inhibitor (NU7441) was assessed by colony formation assays. Data were analyzed by a two-tailed t test. **(L, M)** NTC and CXorf56 knockdown MDA-MB-231 cells were treated with NU7441 (1.5 μM), and γ-H2AX foci before or 48 hours after NU7441 treatment were detected by immunofluorescence. Representative images of γ-H2AX foci are shown in (L), scale bar=10μm. Quantification of focus signals is shown in (M). Data were analyzed by ANOVA and two-tailed t test. Remarks: $^{ns}p \geq 0.05$, $^{*}p < 0.05$, $^{**}p < 0.01$, $^{***}p < 0.001$. Data are presented as mean ± SD.

Supplementary Figure 4

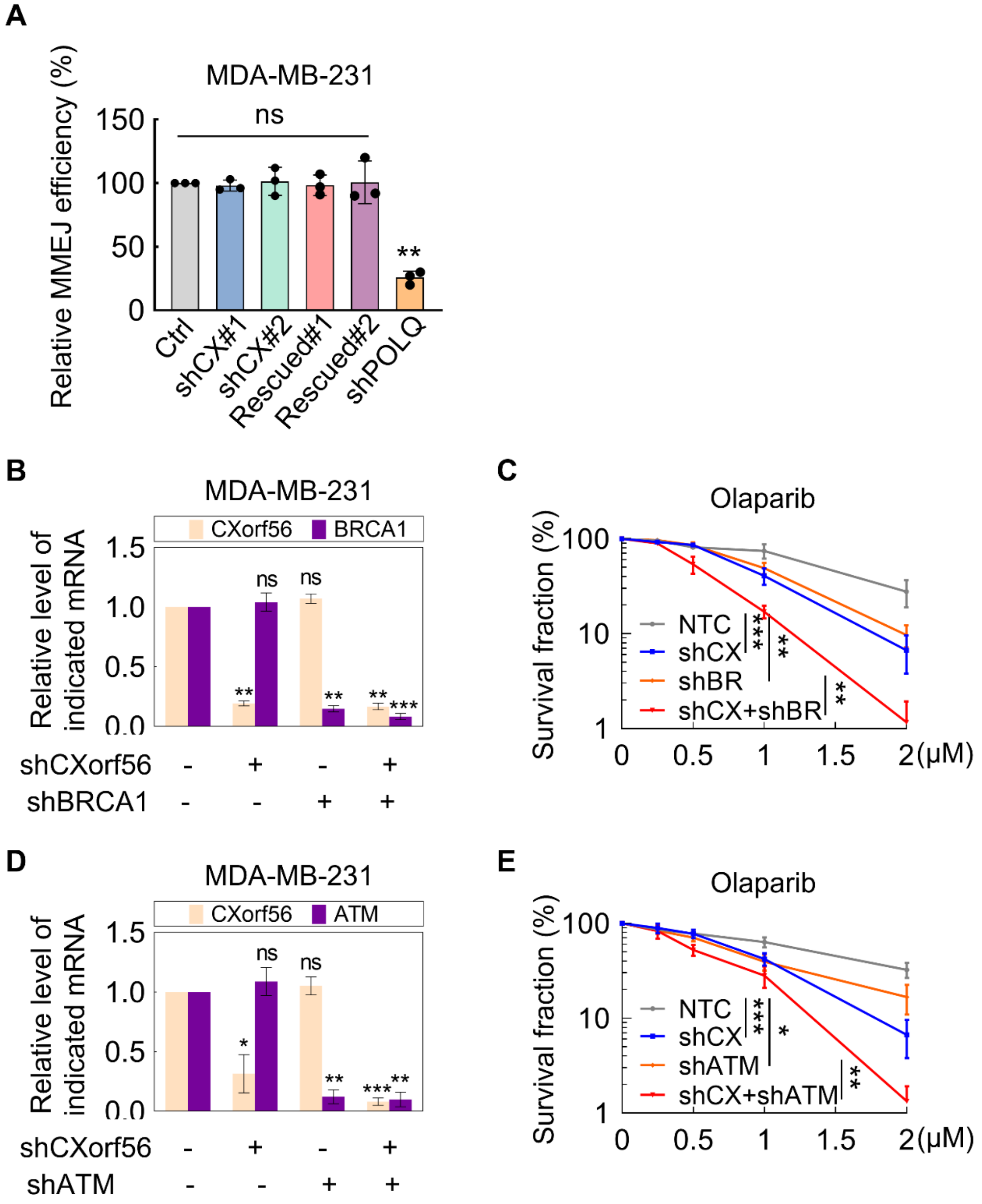

Supplementary Figure 4. Related to Figure 4. (A) The control (Ctrl), CXorf56 knockdown, CXorf56 re-expression, or POLQ knockdown MDA-MB-231 cells with EGFP-MMEJ reporter were transfected with or without lentiviruses encoding Cas9^{WT}, and MMEJ was assayed 5 days after transfection. Data were analyzed by ANOVA and

two-tailed t test. **(B-E)** MDA-MB-231 cells were infected with lentiviruses encoding indicated shRNAs, after validating the knockdown efficiency of each gene (B and D), the sensitivity of the cells in each group to olaparib (C and E) was assessed by colony formation assays. Data were analyzed by a two-tailed t test. Remarks: $^{ns}p \geq 0.05$, $*p < 0.05$, $**p < 0.01$, $***p < 0.001$. Data are presented as mean ± SD.

Supplementary Figure 5

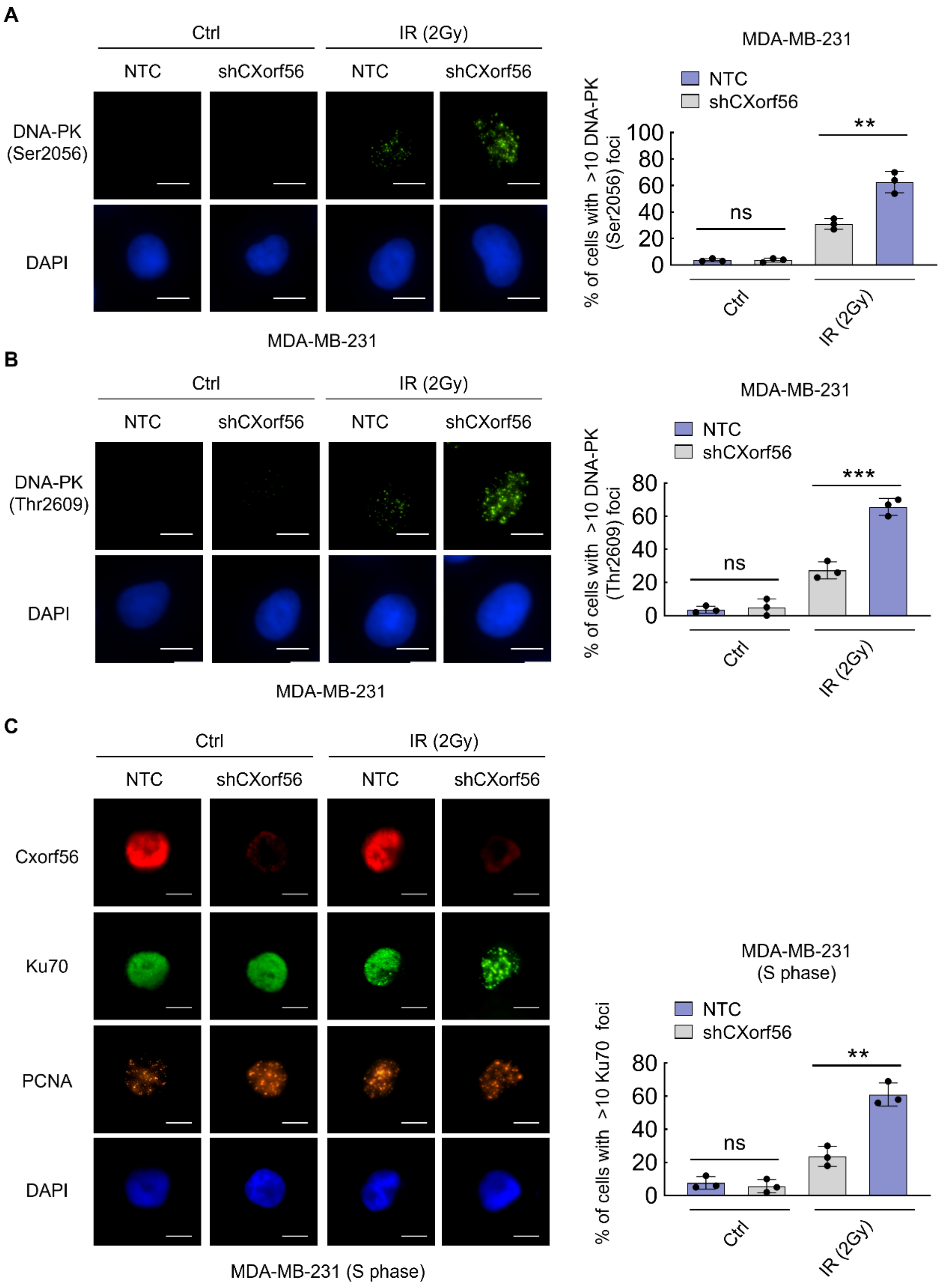

Supplementary Figure 5. Related to Figure 6. (A) Corresponding MDA-MB-231 cells were treated with IR (2 Gy, 1 hour), and phospho-DNA-PKcs (Ser2056) foci were

detected by immunofluorescence. Representative images are shown on the right, and quantification of focus signals is shown on the left. Data were analyzed by a two-tailed t test. **(B)** NTC and CXorf56 MDA-MB-231 cells were treated with IR (2 Gy, 1 hour), and phospho-DNA-PKcs (Thr2609) foci were detected by immunofluorescence. Representative images are shown on the right, and quantification of focus signals is shown on the left. Data were analyzed by a two-tailed t test. **(C)** MDA-MB-231 cells (NTC and CXorf56 knockdown cells) were synchronized with nocodazole (100 ng/ml) for 12 hours and released into the cell cycle. At the 12-hour time points after synchronization, cells were subjected to immunofluorescence following without IR treatment (Ctrl) or with 1-hour IR treatment (2Gy). Representative images are shown on the right, and quantification of focus signals is shown on the left. Data were analyzed by a two-tailed t test. Remarks: $^{ns}p \geq 0.05$, $^{*}p < 0.05$, $^{**}p < 0.01$, $^{***}p < 0.001$. Data are presented as mean ± SD. Scale bars=10μm.

Supplementary Table 1 Breast cancer patient clinicopathological features.

Clinical features	Categories	Number of cases (%)
Age	< 60 years	108 (60.0%)
	>= 60 years	72 (40.0%)
TNBC	No	126 (70.0%)
	Yes	54 (30.0%)
Histologic grade	I-II	99 (55.0%)
	III-IV	81 (45.0%)
Tumor size	< 3 cm	100 (55.6%)
	>= 3 cm	80 (44.4%)
TNM stage	I-II	111 (61.7%)
	III	69 (38.3%)
CXorf56 expression	Low	81 (45.0%)
	High	99 (55.0%)

siRNA sequences

si-NTC:

Sense: 5'-GCACAAGCUGGAGUACAACUACATT-3'

Anti-sense: 5'-UGUAGUUGUACUCCAGCUUGUGCTT-3'

si-CXorf56#1:

Sense: 5'-ACAAACAGUAGUAAACAUGGA-3'

Anti-sense: 5'-CAUGUUUACUACUGUUUGUGC-3'

si-CXorf56#2:

Sense: 5'-UAAAUGGACUCAGUUAAAGAC-3'

Anti-sense: 5'-CUUUAACUGAGUCCAUUUAAC-3'

si-DOT1L#1:

Sense: 5'-AAUUAAAACGUAAUUCUCCAU-3'

Anti-sense: 5'-GGAGAAUUACGUUUUAAUUGA-3'

si-DOT1L#2:

Sense: 5'-UUUCUAAGGAGAUUGUUGCAA-3'

Anti-sense: 5'-GCAACAAUCUCCUUAGAAAGC-3'

si-NFKBIA#1:

Sense: 5'-UUGUACAAAUAUACAAGUCCA-3'

Anti-sense: 5'-GACUUGUAUAUUUGUACAAAA-3'

si-NFKBIA#2:

Sense: 5'-AUCUGUUUAAUAAAUAUACAU-3'

Anti-sense: 5'-GUAUAUUUAUUAAACAGAUUU-3'

si-TAF8#1:

Sense: 5'-UAUUCUGUUCAUAUCUUCCUC-3'

Anti-sense: 5'-GGAAGAUAUGAACAGAAUAAU-3'

si-TAF8#2:

Sense: 5’-AUGUUUCAAGUUGUCUAACCC-3’

Anti-sense: 5’-GUUAGACAACUUGAAACAUUG-3’

si-GTF2H5#1:

Sense: 5’-UAUAAGCACUCCUUUCAAGAC-3’

Anti-sense: 5’-CUUGAAAGGAGUGCUUAUAGA-3’

si-GTF2H5#2:

Sense: 5’-AUACAGAUGGGAAAAUGUGAC-3’

Anti-sense: 5’-CACAUUUUCCCAUCUGUAUUC-3’

si-BCAP31#1:

Sense: 5’-AAUGAGAACCACAAAGAAGGU-3’

Anti-sense: 5’-CUUCUUUGUGGUUCUCAUUGU-3’

si-BCAP31#2:

Sense: 5’-AUCAUACUUCCGAAUUUCGCG-3’

Anti-sense: 5’-CGAAAUUCGGAAGUAUGAUGA-3’

si-SLC31A1#1:

Sense: 5’-AUUCUUAAAGCCAAAGUAGAA-3’

Anti-sense: 5’-CUACUUUGGCUUUAAGAAUGU-3’

si-SLC31A1#2:

Sense: 5’-AAGUCAUUGAACAAAAAGCUA-3’

Anti-sense: 5’-GCUUUUUGUUCAAUGACUUGA-3’

si-GDPD2#1:

Sense: 5’-AAUAGCAAGGAUGCAUAUGUG-3’

Anti-sense: 5’-CAUAUGCAUCCUUGCUAUUGG-3’

si-GDPD2#2:

Sense: 5’-UGUUCUAGUUUCAUUUGAGGA-3’

Anti-sense: 5’-CUCAAAUGAAACUAGAACAGA-3’

si-ZDHHC9#1:

Sense: 5’-AGAACAAAGAUCAAAAUCCGU-3’

Anti-sense: 5’-GGAUUUUGAUCUUUGUUCUUC-3’

si-ZDHHC9#2:

Sense: 5’-UACUUACAUAUAUCUAAUGGG-3’

Anti-sense: 5’-CAUUAGAUAUAUGUAAGUAGU-3’

si-MCTS1#1:

Sense: 5’-UAAAAGGAUAUUUGUGAAGUA-3’

Anti-sense: 5’-CUUCACAAAUAUCCUUUUAUC-3’

si-MCTS1#2:

Sense: 5’-UAUCAUUAGCCUAAGAAAGGA-3’

Anti-sense: 5’-CUUUCUUAGGCUAAUGAUAUA-3’

Regular qPCR primers

CXorf56:

Forward: 5’-ACTGTCAGAGGAGACCCATTA-3’

Reverse: 5’-CCTCCCAAACTGCTAGGATTAC-3’

DOT1L:

Forward: 5’-ACATTGGAGAGAGGCGATTTC-3’

Reverse: 5’-CAAGTATGGTGCGGTCGATAG-3’

NFKBIA:

Forward: 5’-CATCCTGAAGGCTACCAACTAC-3’

Reverse: 5’-CTCTGTGAACTCCGTGAACTC-3’

TAF8:

Forward: 5’-CCTCCCAAAGTGCTGAGATTAC-3’

Reverse: 5’-GGCCAGATGAGGAGAGAAATAAG-3’

GTF2H5:

Forward: 5’-GTCGAAACCCTGTCTCTACAAA-3’

Reverse: 5’-TCCCAAAGTGCTGGGATTAC-3’

BCAP31:

Forward: 5’-GCCCAGAGGAATCTCTACATTG-3’

Reverse: 5’-CCTGTTCTCTTCCTCCAACTTC-3’

SLC31A1:

Forward: 5’-GGGAAATTCTTGCCCAACTAAAC-3’

Reverse: 5’-CCAGACACACCATCTACCAATC-3’

GDPD2:

Forward: 5’-CCAGATGAAGATCGGGCTAATG-3’

Reverse: 5’-CACACCAAAGCAGAGAGAAGAG-3’

ZDHHC9:

Forward: 5’-CTCTCTCCCTCCTCACAATCTA-3’

Reverse: 5’-CAGCTTACTTGCTCTCTGTCTC-3’

MCTS1:

Forward: 5’-AGCAAATCCCAGACATCCTATC-3’

Reverse: 5’-CAGGTCCATGTCCCTCATATTC-3’

GAPDH:

Forward: 5’-AGAAGGCTGGGGCTCATTTG-3’

Reverse: 5’-AGGGGCCATCCACAGTCTTC-3’

shRNA sequences (cloned into pLKO.1-puro vector)

sh-NTC:

Sense: 5’-CCGGTGGTTTACATGTTTTCTGACTCGAGTCAGAAAACATGTAAACCATTTTTG-3’

Anti-sense: 5’-AATTCAAAAATGGTTTACATGTTTTCTGACTCGAGTCAGAAA
ACATGTAAACCA-3’

sh-CXorf56#1:

Sense: 5’-CCGGGGGAATCATGCCGAAAGTATTCAAGAGATACTTTCGGCATG
ATTCCCTTTTTT-3’

Anti-sense: 5’-AATTAAAAAAGGGAATCATGCCGAAAGTATCTCTTGAATACTT
TCGGCATGATTCCC-3’

sh-CXorf56#2:

Sense: 5’-CCGGGCAGAGGAGTGTTGTATATTTCAAGAGAATATACAACACTC
CTCTGCTTTTTT-3’

Anti-sense: 5’-AATTAAAAAAGCAGAGGAGTGTTGTATATTCTCTTGAAATATA
CAACACTCCTCTGC-3’

sh-Ku70:

Sense: 5’-CCGGGGTGGGAGTCATATTACAATTCAAGAGATTGTAATATGACTC
CCACCTTTTTT-3’

Anti-sense: 5’-AATTAAAAAAGGTGGGAGTCATATTACAATCTCTTGAATTGTA
ATATGACTCCCACC-3’

sh-BRCA1:

Sense: 5’-CCGGGCTATGCAAGGGTCCCTTAAATTCAAGAGATTTAAGGGACC
CTTGCATAGCTTTTTT-3’

Anti-sense: 5’-AATTAAAAAAGCTATGCAAGGGTCCCTTAAATCTCTTGAATTT
AAGGGACCCTTGCATAGC-3’

sh-ATM:

Sense: 5’-CCGGGCTATTTACGGAGCTGATTGTTTCAAGAGAACAATCAGCTC

CGTAAATAGCTTTTTT-3’

Anti-sense: 5’-AATTAAAAAAGCTATTTACGGAGCTGATTGTTCTCTTGAAACA

ATCAGCTCCGTAAATAGC-3’

sh-POLQ:

Sense: 5’-CCGGACAACAACCCTTATCGTAAAGTTCAAGAGACTTTACGATAA

GGGTTGTTGTTTTTTT-3’

Anti-sense: 5’-AATTAAAAAAACAACAACCCTTATCGTAAAGTCTCTTGAACT

TTACGATAAGGGTTGTTGT-3’

Overexpression construction primers (cloned into pLenti-CMV-blast vector)

pLenti-CMV-blast-Cxorf56:

Forward: 5’-GGGGATCCGCCACC ATGAGGCCCCGGGACCGGTCCC-3’

Reverse: 5’-CTCGAGCTTGTCGTCATCGTCTTTGTAGTCTTTGAACTGGTTGT

CAATCAAGGT-3’

pLenti-CMV-blast-Ku70-WT:

Forward: 5’-GGGGATCCGCCACCATGTCAGGGTGGGAGTCATATTAC-3’

Reverse: 5’-CTCGAGCTTGTCGTCATCGTCTTTGTAGTCGTCCTGGAAGTGCT

TGGTGAGGG-3’

pLenti-CMV-blast-Ku70-vWa-domain:

Forward: 5’-GGGGATCCGCCACCATGTCAGGGTGGGAGTCATATTAC-3’

Reverse: 5’-CTCGAGCTTGTCGTCATCGTCTTTGTAGTCAAAGTGAACCCTGA

GGTCCTCATCC-3’

pLenti-CMV-blast-Ku70-DNA-binding-domain:

Forward: 5’-GGGGATCCGCCACC GAGGAATCCAGCAAGCTAGAAG-3’

Reverse: 5’-CTCGAGCTTGTCGTCATCGTCTTTGTAGTCAAAGACCAGCTGGA AGCCTGGAGG-3’

pLenti-CMV-blast-Ku70-C-terminal-domain:

Forward: 5’-GGGGATCCGCCACC TTACCCTTTGCTGATGATAAAAGG-3’

Reverse: 5’-CTCGAGCTTGTCGTCATCGTCTTTGTAGTCGTCCTGGAAGTGCT TGGTGAGGG-3’

sgRNAs target sequence (Cas9-reporter):

5’ UTR of LMNA: 5’-GGTTGGCAGCGCTGCCCGCG-3’

Uncropped and unprocessed scans of blots

Figure 4A

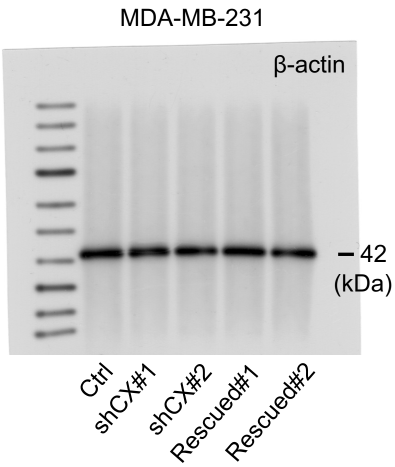

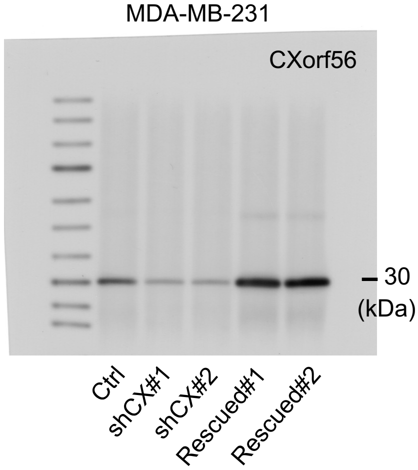

Figure 5A

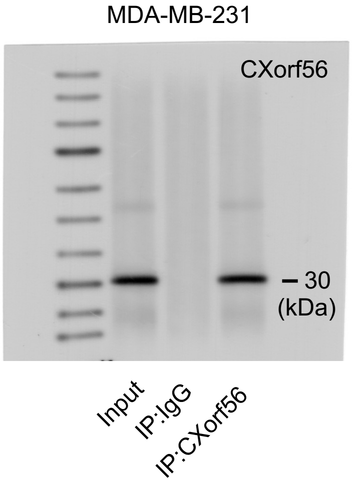

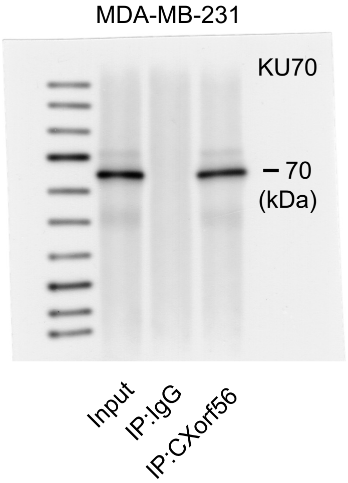

Figure 5B

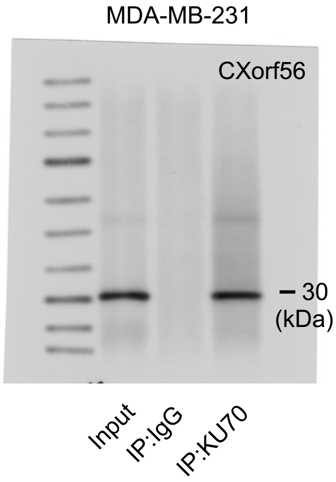

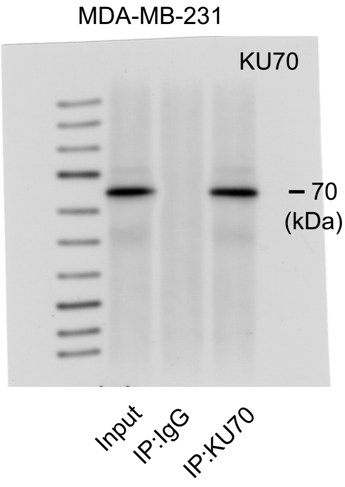

Figure 5C

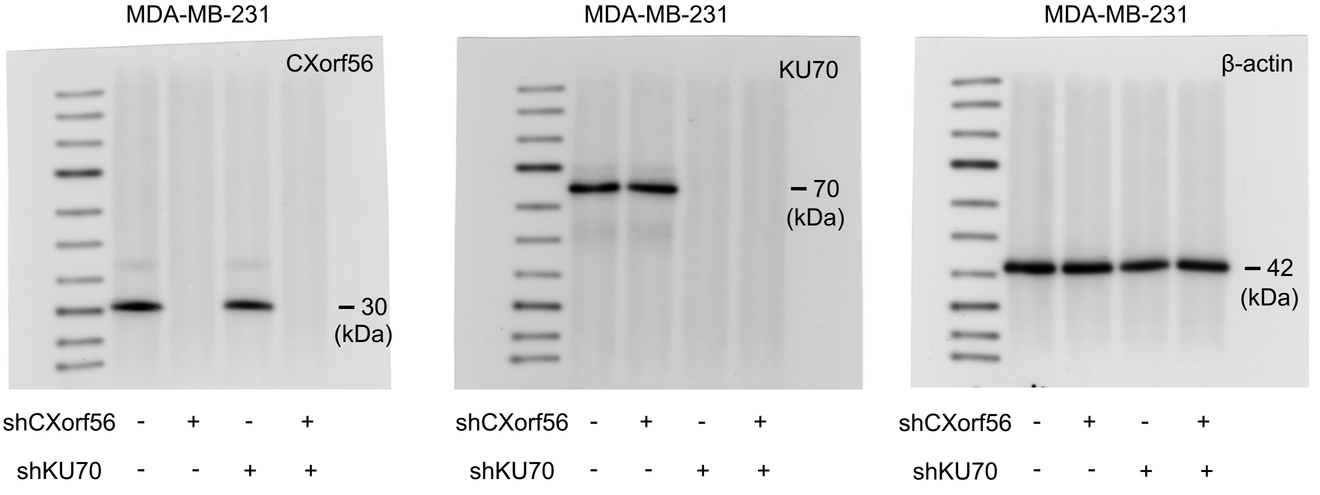

Figure 6B

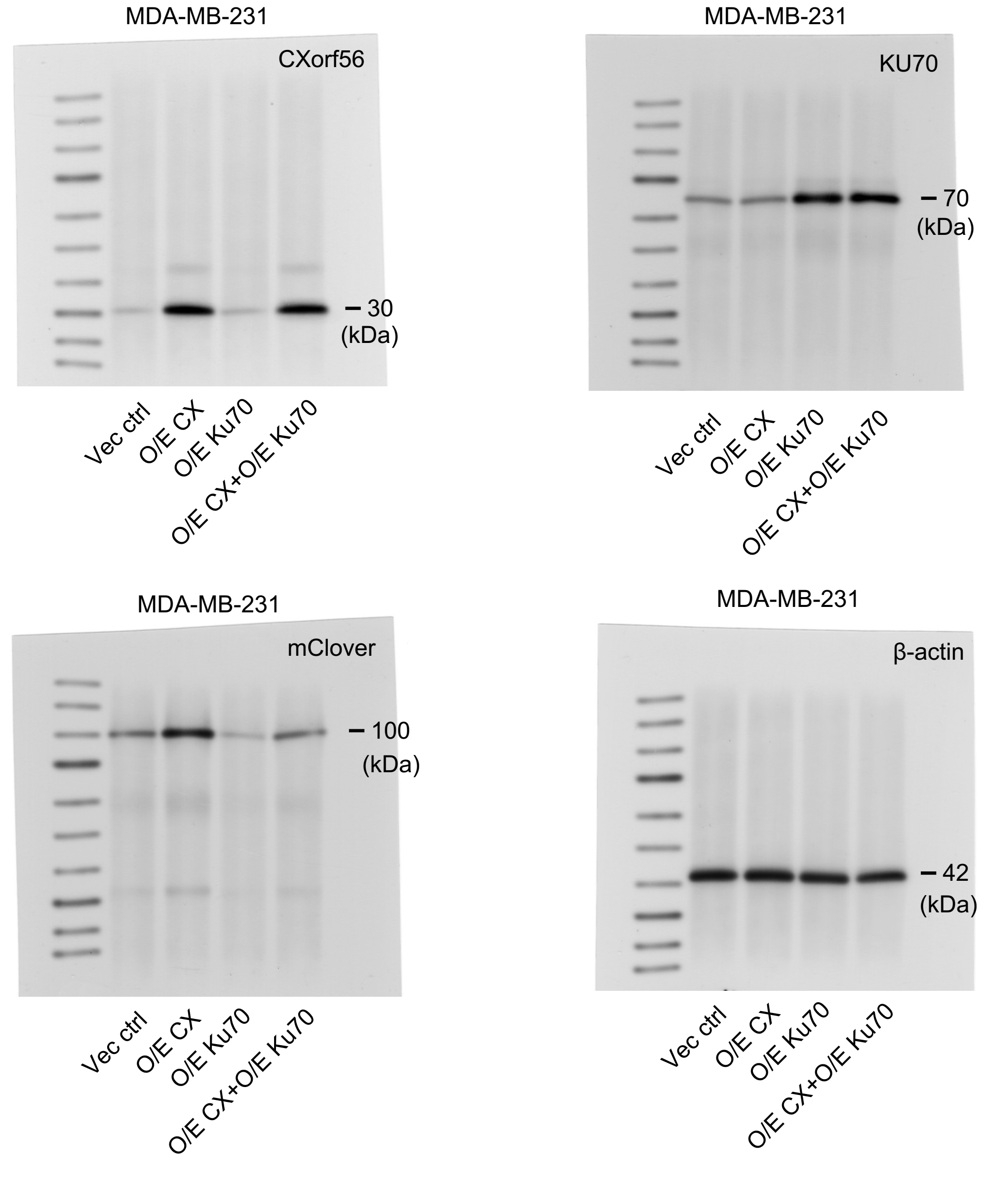

Figure 6G

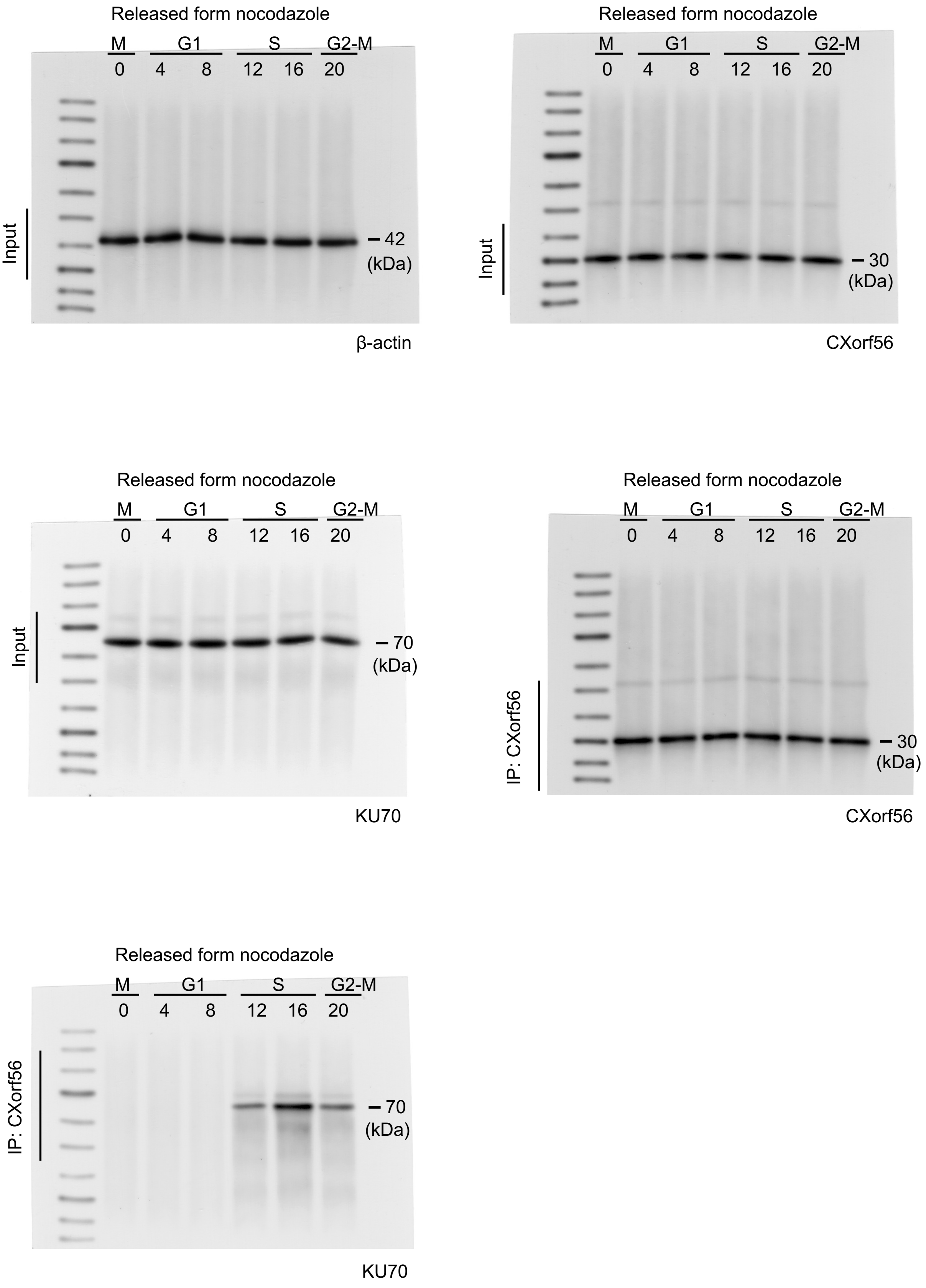
Released form nocodazole
M G1 S G2-M
0 4 8 12 16 20
Input
42 (kDa)
β-actin
Released form nocodazole
M G1 S G2-M
0 4 8 12 16 20
Input
30 (kDa)
CXorf56
Released form nocodazole
M G1 S G2-M
0 4 8 12 16 20
Input
70 (kDa)
KU70
Released form nocodazole
M G1 S G2-M
0 4 8 12 16 20
IP: CXorf56
30 (kDa)
CXorf56
Released form nocodazole
M G1 S G2-M
0 4 8 12 16 20
IP: CXorf56
70 (kDa)
KU70

Figure 6H

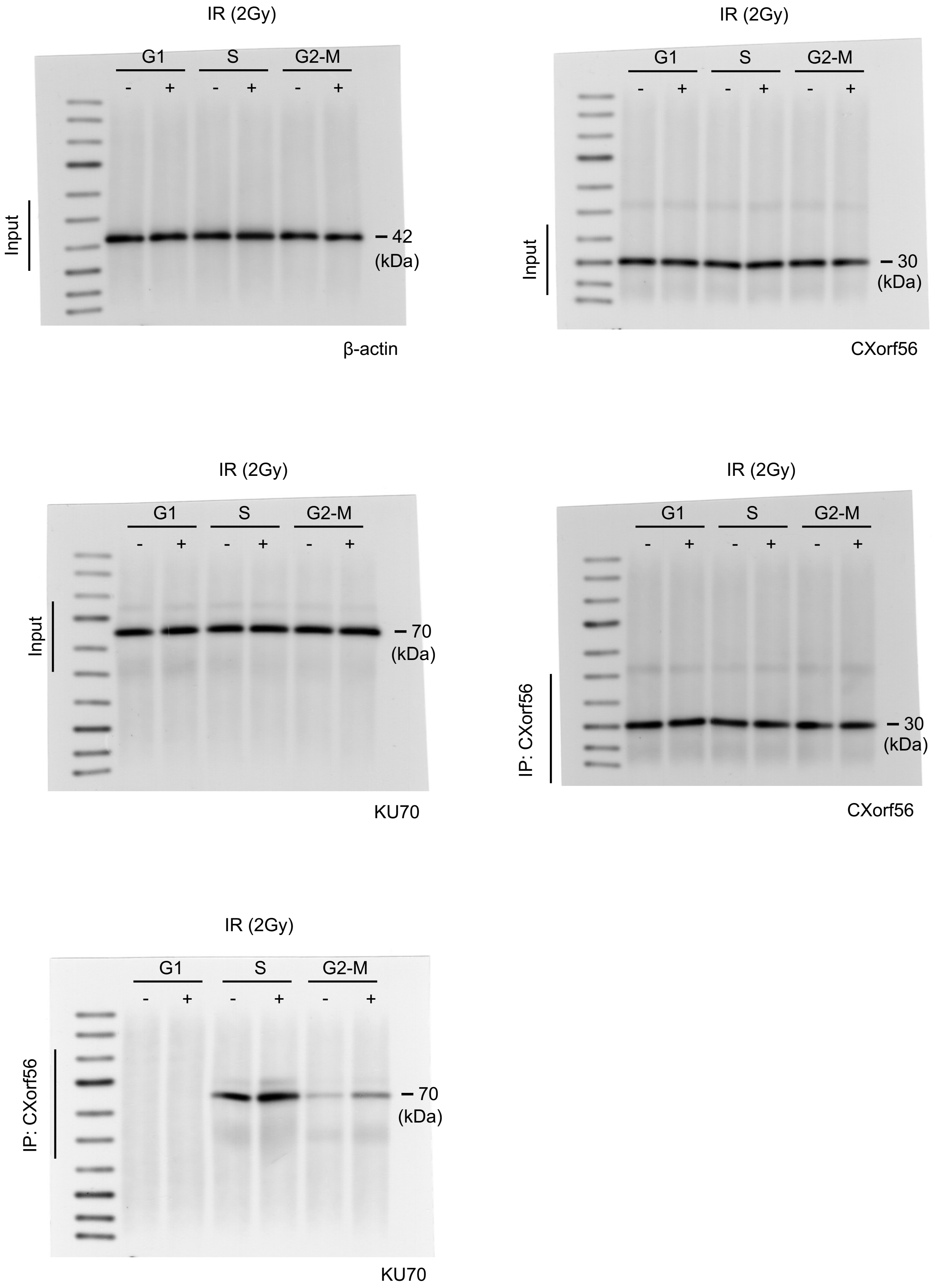
IR (2Gy)
G1
S
G2-M
- + - + - +
Input
42 (kDa)
β-actin
IR (2Gy)
G1
S
G2-M
- + - + - +
Input
30 (kDa)
CXorf56
IR (2Gy)
G1
S
G2-M
- + - + - +
Input
70 (kDa)
KU70
IR (2Gy)
G1
S
G2-M
- + - + - +
IP: CXorf56
30 (kDa)
CXorf56
IR (2Gy)
G1
S
G2-M
- + - + - +
IP: CXorf56
70 (kDa)
KU70

Figure 6J

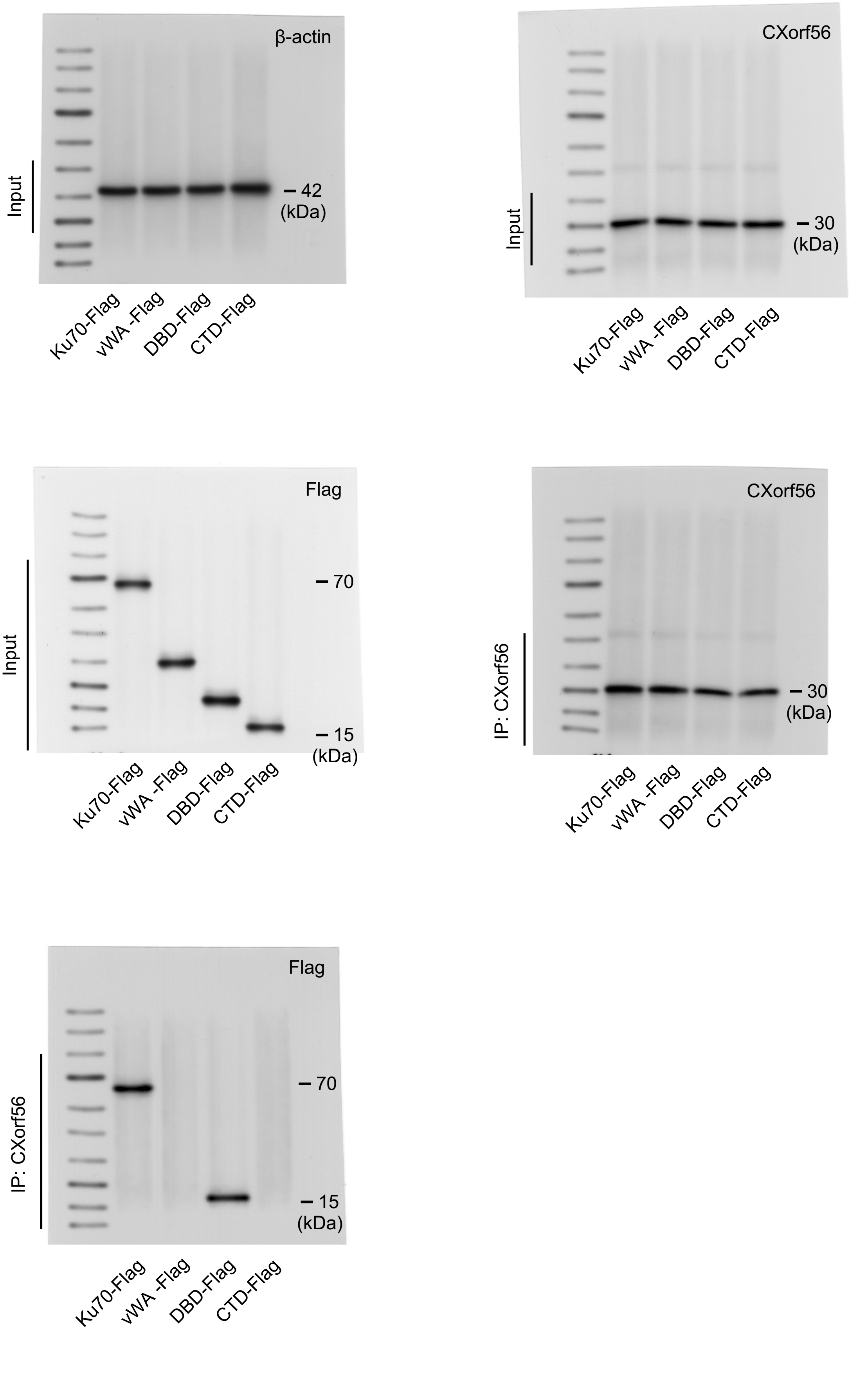
β-actin
Input
42
(kDa)
Ku70-Flag
vWA -Flag
DBD-Flag
CTD-Flag
CXorf56
Input
30
(kDa)
Ku70-Flag
vWA -Flag
DBD-Flag
CTD-Flag
Flag
Input
70
15
(kDa)
Ku70-Flag
vWA -Flag
DBD-Flag
CTD-Flag
CXorf56
IP: CXorf56
30
(kDa)
Ku70-Flag
vWA -Flag
DBD-Flag
CTD-Flag
Flag
IP: CXorf56
70
15
(kDa)
Ku70-Flag
vWA -Flag
DBD-Flag
CTD-Flag

BRCA_survival related genes TOP500

Gene Symbol	Gene ID	P-Value (Survival DFS)	Gene Symbol	Gene ID	P-Value (Survival DFS)
MCTS1	ENSG00000232119.7	2.02E-06	CD3E	ENSG00000198851.9	3.89E-04
OVOS2	ENSG00000177359.17	1.19E-05	RP11-211G3.2	ENSG00000223401.1	4.01E-04
MAPT-IT1	ENSG00000279685.2	1.51E-05	MAFA-AS1	ENSG00000254338.1	4.16E-04
ATG4A	ENSG00000101844.17	1.68E-05	LMAN2	ENSG00000169223.14	4.24E-04
SLC16A2	ENSG00000147100.9	2.15E-05	TRBV5-1	ENSG00000211734.3	4.41E-04
SLC35A2	ENSG00000102100.14	2.67E-05	PAX7	ENSG00000009709.11	4.61E-04
VDAC1	ENSG00000213585.10	3.01E-05	CD74	ENSG00000019582.14	4.66E-04
TBC1D24	ENSG00000162065.11	5.02E-05	NUP43	ENSG00000120253.13	4.69E-04
RP11-214F16.8	ENSG00000280710.2	5.21E-05	TESPA1	ENSG00000135426.14	4.95E-04
PDP1	ENSG00000164951.15	5.29E-05	AC137932.6	ENSG00000261253.2	5.06E-04
TCP1	ENSG00000120438.11	6.14E-05	RP1-193H18.2	ENSG00000267194.1	5.06E-04
QPRT	ENSG00000103485.17	6.21E-05	SLC6A3	ENSG00000142319.17	5.12E-04
TRBC2	ENSG00000211772.8	6.88E-05	PITRM1	ENSG00000107959.15	5.22E-04
C8orf33	ENSG00000182307.12	8.16E-05	TNFRSF13B	ENSG00000240505.8	5.28E-04
RTN4IP1	ENSG00000130347.12	8.77E-05	C10orf67	ENSG00000179133.11	5.32E-04
TP53AIP1	ENSG00000120471.14	8.90E-05	CD40LG	ENSG00000102245.7	5.32E-04
IGHD	ENSG00000211898.7	9.03E-05	RP11-551L14.4	ENSG00000226472.7	5.45E-04
GTF2H5	ENSG00000272047.1	9.59E-05	IVL	ENSG00000163207.6	5.53E-04
FAM155B	ENSG00000130054.4	1.07E-04	AC006042.8	ENSG00000233264.2	5.54E-04
DAPL1	ENSG00000163331.10	1.17E-04	TCN1	ENSG00000134827.7	5.87E-04
LRRC37A6P	ENSG00000230445.4	1.19E-04	KLRB1	ENSG00000111796.3	5.88E-04
CD24	ENSG00000272398.5	1.58E-04	RP11-405A12.1	ENSG00000256849.1	5.91E-04
DCTPP1	ENSG00000179958.8	1.67E-04	FAM159A	ENSG00000182183.14	6.02E-04
PGK1	ENSG00000102144.13	1.72E-04	MORF4L2	ENSG00000123562.16	6.05E-04
TP53I11	ENSG00000175274.18	1.73E-04	FABP7	ENSG00000164434.11	6.08E-04
PVR	ENSG00000073008.14	1.82E-04	MROH7	ENSG00000184313.19	6.08E-04
SEC61G	ENSG00000132432.13	1.98E-04	CD79A	ENSG00000105369.9	6.09E-04
PRR13P5	ENSG00000187534.6	2.07E-04	MAPT-AS1	ENSG00000264589.1	6.13E-04
PRSS1	ENSG00000204983.12	2.08E-04	STAT4	ENSG00000138378.17	6.25E-04
CCDC24	ENSG00000159214.12	2.12E-04	OR51E2	ENSG00000167332.7	6.28E-04
NANOS1	ENSG00000188613.6	2.15E-04	ALG3	ENSG00000214160.9	6.28E-04
HYAL4	ENSG00000106302.9	2.16E-04	RP11-444E17.6	ENSG00000272442.2	6.35E-04
TIMM8A	ENSG00000126953.5	2.38E-04	C8orf76	ENSG00000189376.11	6.43E-04
CYB561	ENSG00000008283.15	2.41E-04	TRBV25-1	ENSG00000211751.7	6.63E-04
ENO1-IT1	ENSG00000236269.1	2.44E-04	CTC-498J12.1	ENSG00000250237.1	6.69E-04
NKAP	ENSG00000101882.9	2.46E-04	PCMT1	ENSG00000120265.16	6.78E-04
RP11-131N11.4	ENSG00000254271.1	2.49E-04	RP6-65G23.5	ENSG00000275630.1	6.90E-04
ZIC2	ENSG00000043355.10	2.55E-04	C11orf84	ENSG00000168005.8	7.16E-04
BCAP31	ENSG00000185825.15	2.61E-04	RP11-431K24.1	ENSG00000238290.1	7.33E-04
RP11-334L9.1	ENSG00000215895.4	2.74E-04	RP11-386M24.4	ENSG00000258741.3	7.35E-04
CTSW	ENSG00000172543.7	2.78E-04	MURC	ENSG00000170681.6	7.39E-04
ZSWIM3	ENSG00000132801.6	2.78E-04	RP11-745A24.2	ENSG00000242667.1	7.46E-04
PXDNL	ENSG00000147485.12	2.79E-04	ARPP21	ENSG00000172995.16	7.47E-04
CHCHD7	ENSG00000170791.17	2.89E-04	AHRR	ENSG00000063438.16	7.65E-04

CYP24A1	ENSG00000019186.9	3.01E-04	RP11-277P12.6	ENSG00000255641.1	7.67E-04
RP11-283G6.5	ENSG00000255750.5	3.07E-04	SLC35C1	ENSG00000181830.8	7.78E-04
ATP5B	ENSG00000110955.8	3.10E-04	AC109333.10	ENSG00000227495.1	7.84E-04
DERL1	ENSG00000136986.9	3.17E-04	KRT6B	ENSG00000185479.5	8.04E-04
PROM2	ENSG00000155066.15	3.22E-04	AC007318.5	ENSG00000172974.11	8.05E-04
VDAC1P2	ENSG00000213856.3	3.23E-04	RP11-116O18.3	ENSG00000267193.5	8.22E-04
CLPTM1L	ENSG00000049656.13	3.44E-04	IGKV2D-24	ENSG00000241566.1	8.30E-04
RP11-44N11.3	ENSG00000272043.1	3.66E-04	RP11-1070N10.3	ENSG00000258572.1	8.32E-04
RP11-693J15.5	ENSG00000205056.8	8.98E-04	SERAC1	ENSG00000122335.13	8.82E-04
AC241377.2	ENSG00000281741.1	9.11E-04	RP11-153M3.1	ENSG00000257576.1	8.90E-04
EMC2	ENSG00000104412.7	9.17E-04	RP11-153M7.3	ENSG00000250771.2	1.58E-03
BTLA	ENSG00000186265.9	9.18E-04	MRPL49	ENSG00000149792.8	1.59E-03
RP11-770G2.2	ENSG00000255557.1	9.39E-04	IKBKAP	ENSG00000070061.14	1.61E-03
ITGAE	ENSG00000083457.11	1.00E-03	CHGA	ENSG00000100604.12	1.65E-03
STXBP5-AS1	ENSG00000233452.6	1.01E-03	ZPR1	ENSG00000109917.10	1.65E-03
PTPN11	ENSG00000179295.15	1.01E-03	TRBV5-5	ENSG00000211725.3	1.66E-03
ICOSLG	ENSG00000160223.16	1.02E-03	DLK2	ENSG00000171462.14	1.67E-03
RP11-567F11.1	ENSG00000281469.1	1.02E-03	CYP2D8P	ENSG00000226450.2	1.67E-03
DTHD1	ENSG00000197057.8	1.06E-03	TMED9	ENSG00000184840.11	1.68E-03
ELAC2	ENSG00000006744.18	1.11E-03	DLG3	ENSG00000082458.11	1.70E-03
GRIK2	ENSG00000164418.19	1.11E-03	ALOX15	ENSG00000161905.12	1.72E-03
RP11-155O18.6	ENSG00000272100.1	1.12E-03	PNO1	ENSG00000115946.7	1.73E-03
LINC00861	ENSG00000245164.6	1.12E-03	C1GALT1C1	ENSG00000171155.7	1.73E-03
IFNG	ENSG00000111537.4	1.13E-03	SNX12	ENSG00000147164.11	1.74E-03
MRPS28	ENSG00000147586.9	1.15E-03	MYBPH	ENSG00000133055.8	1.75E-03
RAC2	ENSG00000128340.14	1.17E-03	CTC-231O11.1	ENSG00000253522.3	1.75E-03
RP4-635E18.7	ENSG00000226849.1	1.17E-03	PLAT	ENSG00000104368.17	1.75E-03
TMEM31	ENSG00000179363.6	1.17E-03	DNAJC5	ENSG00000101152.10	1.75E-03
NDUFAB1	ENSG00000004779.9	1.18E-03	SLC25A5	ENSG00000005022.5	1.76E-03
FAM127B	ENSG00000203950.6	1.18E-03	PCED1B-AS1	ENSG00000247774.6	1.76E-03
PLAC8	ENSG00000145287.10	1.19E-03	JCHAIN	ENSG00000132465.10	1.78E-03
PDXP	ENSG00000241360.1	1.19E-03	SH3D21	ENSG00000214193.9	1.80E-03
PFN1P1	ENSG00000233328.3	1.19E-03	ESRP1	ENSG00000104413.15	1.80E-03
ZNF75D	ENSG00000186376.14	1.19E-03	CCT8	ENSG00000156261.12	1.81E-03
NRBP1	ENSG00000115216.13	1.20E-03	TMSB15A	ENSG00000158164.6	1.83E-03
PRPF18	ENSG00000165630.13	1.20E-03	MTRF1L	ENSG00000112031.15	1.83E-03
MORC4	ENSG00000133131.14	1.20E-03	RP11-641D5.1	ENSG00000213178.3	1.84E-03
SLC27A6	ENSG00000113396.12	1.20E-03	NUPR1	ENSG00000176046.8	1.85E-03
GCGR	ENSG00000215644.9	1.23E-03	ITK	ENSG00000113263.12	1.85E-03
PDSS2	ENSG00000164494.11	1.24E-03	MED27	ENSG00000160563.13	1.86E-03
TAPBPL	ENSG00000139192.11	1.26E-03	FAM181B	ENSG00000182103.4	1.86E-03
RP11-472K17.3	ENSG00000279965.1	1.26E-03	TRAV26-1	ENSG00000211807.3	1.86E-03
GRPEL1	ENSG00000109519.12	1.27E-03	REPS1	ENSG00000135597.18	1.86E-03
COX18	ENSG00000163626.16	1.29E-03	IGKV6-21	ENSG00000211611.2	1.88E-03
AP1S1	ENSG00000106367.13	1.29E-03	TRBV14	ENSG00000275743.1	1.92E-03

CAMKV	ENSG00000164076.16	1.31E-03	FIG4	ENSG00000112367.10	1.94E-03
RP11-530A18.1	ENSG00000220685.3	1.33E-03	DHX40P1	ENSG00000266992.1	1.94E-03
TRBV29-1	ENSG00000232869.2	1.33E-03	ELOVL2-AS1	ENSG00000230314.6	1.97E-03
TRAT1	ENSG00000163519.13	1.34E-03	CDKL3	ENSG00000006837.11	1.97E-03
ARID1B	ENSG00000049618.21	1.37E-03	FAM92B	ENSG00000153789.12	1.98E-03
MIA	ENSG00000261857.6	1.38E-03	SLC22A11	ENSG00000168065.15	1.99E-03
RP11-285E23.2	ENSG00000274560.1	1.38E-03	PPP1R14D	ENSG00000166143.9	2.01E-03
RAB1B	ENSG00000174903.14	1.39E-03	AGPAT1	ENSG00000204310.10	2.02E-03
TPD52	ENSG00000076554.15	1.40E-03	KIAA0125	ENSG00000226777.7	2.02E-03
AC093616.4	ENSG00000234231.3	1.41E-03	ATP5J	ENSG00000154723.12	2.04E-03
TPD52L2	ENSG00000101150.17	1.42E-03	SURF4	ENSG00000148248.13	2.05E-03
LACE1	ENSG00000135537.16	1.42E-03	TCL1A	ENSG00000100721.10	2.05E-03
KRT17	ENSG00000128422.15	1.44E-03	RP11-728K20.2	ENSG00000260555.1	2.06E-03
SERPINA1	ENSG00000197249.12	1.49E-03	SH2D1A	ENSG00000183918.14	2.06E-03
TRAV8-4	ENSG00000211790.2	1.52E-03	LAMP2	ENSG00000005893.15	2.07E-03
EDAR	ENSG00000135960.9	2.12E-03	RPL4P1	ENSG00000240954.1	2.12E-03
TNFRSF13C	ENSG00000159958.4	2.12E-03	RPL14	ENSG00000188846.13	2.13E-03
SEPT14P19	ENSG00000281379.2	2.91E-03	RP11-674P19.2	ENSG00000264705.1	2.15E-03
CHPF	ENSG00000123989.13	2.91E-03	RP11-480D4.2	ENSG00000249159.6	2.16E-03
TBX21	ENSG00000073861.2	2.92E-03	UNC50	ENSG00000115446.11	2.16E-03
KDELR1	ENSG00000105438.8	2.93E-03	AC073130.1	ENSG00000237870.6	2.17E-03
PGM5P2	ENSG00000277778.1	2.94E-03	HRH2	ENSG00000113749.6	2.17E-03
GRAMD1C	ENSG00000178075.19	2.96E-03	GFI1	ENSG00000162676.11	2.18E-03
RP11-423H2.1	ENSG00000170089.15	2.98E-03	TRBV13	ENSG00000276405.1	2.24E-03
TMC8	ENSG00000167895.14	3.00E-03	PPP2R5B	ENSG00000068971.13	2.24E-03
DYNLT1	ENSG00000146425.10	3.02E-03	CHCHD4	ENSG00000163528.12	2.25E-03
SDAD1	ENSG00000198301.11	3.03E-03	AC006129.2	ENSG00000268027.5	2.25E-03
SORBS1	ENSG00000095637.20	3.04E-03	RP11-20G13.5	ENSG00000274294.1	2.26E-03
UGT3A2	ENSG00000168671.9	3.05E-03	ACAD8	ENSG00000151498.11	2.27E-03
PLEKHA4	ENSG00000105559.11	3.07E-03	LINC01215	ENSG00000271856.1	2.28E-03
IL18RAP	ENSG00000115607.9	3.08E-03	RP4-740C4.5	ENSG00000269896.2	2.28E-03
IGKV6D-21	ENSG00000225523.2	3.08E-03	SCAANT1	ENSG00000280620.1	2.29E-03
NDUFV3	ENSG00000160194.17	3.12E-03	SEMA4G	ENSG00000095539.15	2.30E-03
NIPAL2	ENSG00000104361.9	3.13E-03	P4HA2	ENSG00000072682.18	2.31E-03
TMEM242	ENSG00000215712.10	3.13E-03	OTOF	ENSG00000115155.16	2.34E-03
LINC01224	ENSG00000269416.5	3.14E-03	CXorf56	ENSG00000018610.13	2.36E-03
EGR3	ENSG00000179388.8	3.16E-03	CDC42BPA	ENSG00000143776.18	2.37E-03
IGKV2D-30	ENSG00000239571.1	3.16E-03	SYS1-DBNDD2	ENSG00000254806.5	2.38E-03
SEPHS1P6	ENSG00000213938.3	3.16E-03	AIMP2	ENSG00000106305.9	2.41E-03
RNASE10	ENSG00000182545.6	3.18E-03	RP11-762L8.6	ENSG00000278989.1	2.42E-03
CTC-366B18.2	ENSG00000248881.1	3.19E-03	TNFAIP8L2	ENSG00000163154.5	2.43E-03
C4BPA	ENSG00000123838.10	3.20E-03	UBASH3A	ENSG00000160185.13	2.45E-03
MINA	ENSG00000170854.17	3.20E-03	CYTIP	ENSG00000115165.9	2.45E-03
CPT1A	ENSG00000110090.12	3.21E-03	RP11-406H23.2	ENSG00000259113.1	2.46E-03
RP11-50D9.1	ENSG00000244021.4	3.22E-03	PPAP2C	ENSG00000141934.9	2.46E-03

CDC40	ENSG00000168438.14	3.23E-03
TMIGD2	ENSG00000167664.8	3.23E-03
ZNF750	ENSG00000141579.6	3.25E-03
RP11-120K24.5	ENSG00000269376.1	3.26E-03
GPN3	ENSG00000111231.8	3.26E-03
BDH1	ENSG00000161267.11	3.27E-03
AP000320.6	ENSG00000225555.1	3.27E-03
ATP5C1	ENSG00000165629.19	3.28E-03
MRPL18	ENSG00000112110.9	3.28E-03
RP11-532M24.1	ENSG00000272486.1	3.29E-03
RP11-379K17.4	ENSG00000239219.2	3.30E-03
RTN3	ENSG00000133318.13	3.31E-03
SLC25A32	ENSG00000164933.11	3.33E-03
ABCB9	ENSG00000150967.17	3.33E-03
TRAV5	ENSG00000211779.3	3.34E-03
CDK8	ENSG00000132964.11	3.34E-03
LINC00926	ENSG00000247982.6	3.34E-03
PTK2	ENSG00000169398.19	3.35E-03
NDUFAF6	ENSG00000156170.12	3.37E-03
ANKRD55	ENSG00000164512.17	3.37E-03
CD163L1	ENSG00000177675.8	3.41E-03
MAP2K6	ENSG00000108984.13	3.42E-03
RP11-254F19.4	ENSG00000279622.1	3.55E-03
SLFN12L	ENSG00000205045.8	3.56E-03
SEC13	ENSG00000157020.17	3.56E-03
RP11-144L1.8	ENSG00000231434.1	3.57E-03
BCAR4	ENSG00000262117.5	3.58E-03
CD96	ENSG00000153283.12	3.59E-03
RP11-126L15.4	ENSG00000236305.1	3.60E-03
KIF21A	ENSG00000139116.17	3.60E-03
CCT4	ENSG00000115484.14	3.60E-03
KIAA0196	ENSG00000164961.15	3.61E-03
AP000442.1	ENSG00000255139.1	3.62E-03
SDCCAG3	ENSG00000165689.16	3.64E-03
CD5	ENSG00000110448.10	3.64E-03
NEFH	ENSG00000100285.9	3.65E-03
TRAC	ENSG00000277734.4	3.65E-03
PHGR1	ENSG00000233041.8	3.65E-03
CXCL2	ENSG00000081041.8	3.67E-03
GDPD2	ENSG00000130055.13	3.72E-03
KRT8P32	ENSG00000250221.1	3.72E-03
DRP2	ENSG00000102385.12	3.73E-03
UBE2V1	ENSG00000244687.11	3.73E-03
RP11-49I11.1	ENSG00000260552.1	3.74E-03
RP11-91P24.6	ENSG00000255449.1	3.77E-03
NDUFAF4	ENSG00000123545.5	2.49E-03
ROPN1B	ENSG00000114547.9	2.51E-03
SLC7A3	ENSG00000165349.11	2.53E-03
AP5S1	ENSG00000125843.10	2.53E-03
ASAP1-IT2	ENSG00000280543.1	2.57E-03
BYSL	ENSG00000112578.9	2.58E-03
RP11-522I20.3	ENSG00000254473.1	2.59E-03
CAMK4	ENSG00000152495.10	2.60E-03
BLK	ENSG00000136573.12	2.60E-03
SUMO1P3	ENSG00000235082.2	2.60E-03
BRMS1L	ENSG00000100916.13	2.61E-03
AC010900.2	ENSG00000232479.2	2.61E-03
CCNE1	ENSG00000105173.13	2.63E-03
ADAMTSL2	ENSG00000197859.9	2.63E-03
CDH22	ENSG00000149654.9	2.64E-03
IGLC7	ENSG00000211685.3	2.69E-03
MAL2	ENSG00000147676.13	2.70E-03
SLC31A1	ENSG00000136868.10	2.73E-03
LSMEM2	ENSG00000179564.3	2.77E-03
RP4-803A2.2	ENSG00000239670.1	2.78E-03
PSMD8	ENSG00000099341.11	2.78E-03
PAFAH1B1	ENSG00000007168.12	2.83E-03
TMEM55A	ENSG00000155099.7	3.49E-03
SLC26A4-AS1	ENSG00000233705.6	3.50E-03
STAR	ENSG00000147465.11	3.54E-03
PPARD	ENSG00000112033.13	4.21E-03
RP11-519G16.5	ENSG00000259342.1	4.21E-03
HSP90AB3P	ENSG00000183199.6	4.21E-03
AIMP1	ENSG00000164022.16	4.21E-03
IGHV3-74	ENSG00000224650.2	4.23E-03
TRAF3IP3	ENSG00000009790.14	4.23E-03
CXCL14	ENSG00000145824.12	4.27E-03
MAGOHB	ENSG00000111196.9	4.29E-03
SKAP1	ENSG00000141293.15	4.31E-03
MGAT4EP	ENSG00000184774.8	4.33E-03
RP11-75L1.2	ENSG00000213443.2	4.35E-03
GGCT	ENSG00000006625.17	4.40E-03
PRKAB2	ENSG00000131791.7	4.44E-03
IYD	ENSG00000009765.14	4.53E-03
VDAC1P8	ENSG00000229036.7	4.54E-03
LARP6	ENSG00000166173.10	4.54E-03
ARMC1	ENSG00000104442.9	4.54E-03
APOO	ENSG00000184831.13	4.56E-03
RP11-91P24.1	ENSG00000241782.1	4.56E-03
ST8SIA6-AS1	ENSG00000204832.9	4.56E-03

RBP7	ENSG00000162444.11	3.77E-03	RBP1	ENSG00000114115.9	4.57E-03
RP11-481F24.3	ENSG00000278928.1	3.77E-03	BMP5	ENSG00000112175.7	4.60E-03
UBE2A	ENSG00000077721.15	3.78E-03	CCL23	ENSG00000274736.4	4.60E-03
TF	ENSG00000091513.14	3.80E-03	CRLS1	ENSG00000088766.11	4.63E-03
IMMT	ENSG00000132305.20	3.81E-03	OTUD6B-AS1	ENSG00000253738.1	4.63E-03
RP11-538D16.2	ENSG00000224810.1	3.82E-03	RP11-263K19.4	ENSG00000231064.5	4.66E-03
SPIB	ENSG00000269404.6	3.82E-03	HRSP12	ENSG00000132541.10	4.67E-03
RP11-26H16.1	ENSG00000232037.3	3.84E-03	COX7B	ENSG00000131174.4	4.68E-03
CD52	ENSG00000169442.8	3.86E-03	NXNL2	ENSG00000130045.15	4.70E-03
PEX7	ENSG00000112357.12	3.86E-03	LINC01297	ENSG00000274827.4	4.71E-03
RP11-386M24.6	ENSG00000260337.3	3.86E-03	TMC7	ENSG00000170537.12	4.72E-03
FDCSP	ENSG00000181617.5	3.90E-03	KRT14	ENSG00000186847.5	4.72E-03
FOXP4-AS1	ENSG00000234753.5	3.90E-03	SLC25A14	ENSG00000102078.15	4.73E-03
EGOT	ENSG00000235947.1	3.91E-03	APC2	ENSG00000115266.11	4.74E-03
PABPC3	ENSG00000151846.8	3.92E-03	RP11-35O15.2	ENSG00000278022.1	4.74E-03
CACNA1H	ENSG00000196557.10	3.92E-03	IGHA2	ENSG00000211890.3	4.74E-03
ARMC12	ENSG00000157343.8	3.93E-03	AC104699.1	ENSG00000224220.1	4.75E-03
APOBEC3G	ENSG00000239713.7	3.94E-03	ATP6V1H	ENSG00000047249.16	4.77E-03
CYP4F12	ENSG00000186204.14	3.98E-03	RLN2	ENSG00000107014.8	4.81E-03
SPECC1	ENSG00000128487.16	4.00E-03	PGAM1	ENSG00000171314.8	4.81E-03
MYBPC1	ENSG00000196091.12	4.02E-03	RP11-79H23.3	ENSG00000261618.1	4.81E-03
TFB1M	ENSG00000029639.10	4.02E-03	NFKBIA	ENSG00000100906.10	4.81E-03
RP11-222K16.2	ENSG00000272282.1	4.07E-03	CTD-2561B21.5	ENSG00000261924.1	4.82E-03
CLSTN1	ENSG00000171603.16	4.08E-03	TUBAP2	ENSG00000214391.3	4.84E-03
FAM179A	ENSG00000189350.12	4.10E-03	HLA-K	ENSG00000230795.2	4.86E-03
VSIG8	ENSG00000243284.1	4.14E-03	RP11-516A11.1	ENSG00000228328.2	4.89E-03
RP13-39P12.2	ENSG00000213513.3	4.15E-03	SLC20A2	ENSG00000168575.9	4.89E-03
ADORA3	ENSG00000282608.1	4.16E-03	C12orf54	ENSG00000177627.9	4.91E-03
PSMD10	ENSG00000101843.18	4.17E-03	APOOL	ENSG00000155008.13	4.91E-03
SCML4	ENSG00000146285.13	4.95E-03	HSPA8P1	ENSG00000234176.1	3.66E-04
CD8B	ENSG00000172116.21	4.95E-03	AIFM1	ENSG00000156709.13	3.79E-04
TMEM65	ENSG00000164983.7	4.98E-03	RP11-686D22.10	ENSG00000267554.1	3.83E-04
RP11-7I15.3	ENSG00000254829.1	5.00E-03	KRT15	ENSG00000171346.13	8.98E-04
LINC01235	ENSG00000270547.5	5.02E-03	LRP11	ENSG00000120256.9	8.39E-04
CD2	ENSG00000116824.4	5.03E-03	HDAC2	ENSG00000196591.11	8.47E-04
MSRB1	ENSG00000198736.11	5.03E-03	IGHV3-64	ENSG00000223648.3	8.56E-04
GARS	ENSG00000106105.13	5.04E-03	TARS	ENSG00000113407.13	8.80E-04
CXCL9	ENSG00000138755.5	5.04E-03	PDCD2	ENSG00000071994.10	1.53E-03
RP11-1109F11.3	ENSG00000271327.1	5.04E-03	IGF2R	ENSG00000197081.12	1.53E-03
SETD7	ENSG00000145391.13	5.07E-03	CTD-2506J14.1	ENSG00000246084.2	1.54E-03
RAPSN	ENSG00000165917.9	5.09E-03	HLA-DQB2	ENSG00000232629.8	1.58E-03
RGL4	ENSG00000159496.14	5.12E-03	INPP5A	ENSG00000068383.18	2.09E-03
CDCA7	ENSG00000144354.13	5.13E-03	PICALM	ENSG00000073921.17	2.10E-03
RP11-48B3.4	ENSG00000260317.1	5.15E-03	ST7-AS1	ENSG00000227199.1	2.11E-03
CXCL16	ENSG00000161921.14	5.18E-03	TAF8	ENSG00000137413.15	2.11E-03

C2CD2	ENSG00000157617.16	5.22E-03	RPL7P22	ENSG00000226544.4	3.43E-03
CTD-2591A1.1	ENSG00000280159.1	5.26E-03	ELOVL2	ENSG00000197977.3	3.45E-03
CCT7	ENSG00000135624.15	5.26E-03	DACT2	ENSG00000164488.11	3.46E-03
APOA1	ENSG00000118137.9	5.27E-03	LINC00402	ENSG00000235532.1	3.47E-03
C6orf99	ENSG00000203711.11	5.27E-03	HSP90B2P	ENSG00000259706.1	2.84E-03
DSTN	ENSG00000125868.15	5.31E-03	STC2	ENSG00000113739.10	2.85E-03
RP5-1042I8.7	ENSG00000261662.1	5.32E-03	PRSS27	ENSG00000172382.9	2.90E-03
NME9	ENSG00000181322.13	5.32E-03	ATP6V1E1	ENSG00000131100.12	2.90E-03
RPS11P5	ENSG00000232888.4	5.32E-03	CATSPERG	ENSG00000099338.22	4.18E-03
PSMD2	ENSG00000175166.16	5.33E-03	RP5-844F9.1	ENSG00000279853.1	4.19E-03
ZDHHC9	ENSG00000188706.12	5.34E-03	MTDH	ENSG00000147649.9	4.19E-03
SLC7A5	ENSG00000103257.8	5.35E-03	IL24	ENSG00000162892.15	4.20E-03
RMDN1	ENSG00000176623.11	5.36E-03	DUSP5P1	ENSG00000183929.7	4.92E-03
DOT1L	ENSG00000104885.17	5.36E-03	MAP4K1	ENSG00000104814.12	4.92E-03
RP11-294C11.4	ENSG00000281179.1	5.38E-03	BCL2L14	ENSG00000121380.12	4.93E-03
IGHV1OR15-1	ENSG00000270505.1	5.38E-03	MLX	ENSG00000108788.11	4.93E-03
RP11-332H14.1	ENSG00000272861.1	5.41E-03			
HEPN1	ENSG00000221932.6	5.41E-03			
OCRL	ENSG00000122126.15	5.44E-03			
FXYD7	ENSG00000221946.7	5.47E-03			
RP11-689B22.2	ENSG00000257221.1	5.52E-03			
RORB	ENSG00000198963.10	5.53E-03			
TRNT1	ENSG00000072756.16	5.54E-03			
LRRC2	ENSG00000163827.12	5.54E-03			
DCTN2	ENSG00000175203.15	5.55E-03			
TNFRSF14	ENSG00000157873.17	5.57E-03			
RP11-114O18.1	ENSG00000273406.1	5.58E-03			
PDCD6	ENSG00000249915.7	5.58E-03			
CTA-280A3.2	ENSG00000279712.1	5.61E-03			
IL27RA	ENSG00000104998.3	5.62E-03			
LINC01281	ENSG00000235304.1	5.62E-03			
YWHAB	ENSG00000166913.12	5.64E-03			
SYCP3	ENSG00000139351.14	5.66E-03			
TMEM70	ENSG00000175606.10	5.67E-03			
GNPNAT1	ENSG00000100522.8	5.67E-03			
SFTPD	ENSG00000133661.15	5.70E-03			

Candidate Gene

Gene Symbol	Gene ID	P-Value (Survival OS)
MCTS1	ENSG00000232119.7	2.02E-06
GDPD2	ENSG00000130055.13	3.72E-03
ZDHHC9	ENSG00000188706.12	5.34E-03
TAF8	ENSG00000137413.15	2.11E-03
SLC31A1	ENSG00000136868.10	2.73E-03
BCAP31	ENSG00000185825.15	2.61E-04
GTF2H5	ENSG00000272047.1	9.59E-05
DOT1L	ENSG00000104885.17	5.36E-03
CXorf56	ENSG00000018610.13	2.36E-03
NFKBIA	ENSG00000100906.10	4.81E-03

DNA damage related genes

B3GALT5	SRCAP	SLC18B1	SPOP	NAA35	SLC7A6OS	PSMC3IP
BARD1	TBC1D20	TONSL	STK19	NUB1	SOD2	SLC6A5
BRCA1	TFDP1	UBE2H	UFL1	PCBP1	STAG1	TMEM165
BRIP1	TMEM208	USP7	UFSP2	PITPNM1	STARD7	TMEM2
C17orf70	TRA2B	WDR26	XPC	POLA2	STOML2	MYL5
C19orf40	UBE2R2	WIPI2	AP2M1	PPP1R3A	TAX1BP1	SECISBP2
COG5	VCPIP1	XRN1	AP2S1	PRIM1	TCEB2	CCDC15
DSCC1	VTA1	YPEL5	AUNIP	RAMP1	THRAP3	CLCN3
ELOF1	YIPF5	AHR	BLM	RNF113A	TMX2	ESR1
ENO1	ZNHIT1	AIP	BOD1L1	ROCK2	TP53BP1	FAM208B
ERCC1	MYBL1	ANKRD49	BRAP	RPA1	UIMC1	GPRC5D
ERCC2	AHCYL1	ARIH2	BRCA2	RPA2	XRCC1	LIPT1
ERCC4	CERS2	ARNT	C17orf53	SERINC4	XRCC4	RAD51
ERCC6	GATA6	ATG13	C1orf112	SLC39A10	YRDC	RWDD4
ERCC8	GNA12	BCL3	CCAR1	SNRNP40	ZFX	CABIN1
FANCA	LGI3	C9orf139	CHTF8	SPAG9	ABCC1	CDK10
FANCB	MND1	CAPZB	CNOT1	TEN1	ASIC3	CKS2
FANCC	NONO	CLN8	CYB5R4	TMX1	ATP8B1	EIF3K
FANCD2	SDCBP	CUL3	DCLRE1A	TP53	BMPR1A	ENY2
FANCE	ZNF207	CUL5	DRAP1	TRIM37	C7orf49	FBXO44
FANCF	CDC25B	DPH1	ECHS1	TRIP12	CBY1	HIRA
FANCG	CDK12	EREG	EME1	TSSC1	CDC37	KANSL3
FANCI	COPS8	F2R	ESCO1	UBE2F	CRAMP1L	KDM5A
FANCL	CPSF4	FKBPL	ETS1	UBE2L3	HIST1H1B	LCMT1
FANCM	DAPK3	FZR1	FAN1	UBE2S	HIST1H1C	MNAT1
FLT4	DPY19L2	INTS5	HELQ	UBOX5	NFRKB	MTA2
FUT1	EDC3	IRF2BPL	INTS10	ZCCHC14	PPM1G	NPHP1
GALM	ELAVL1	ITGB1	KIAA1524	AGK	SMAD4	NUP188
GORASP1	FOXM1	KEAP1	LEO1	ATM	SMARCB1	PHF12
GTF2H5	HDGFRP2	MAPK14	MCM8	C7orf26	SYCE1	PPIL1
HUS1	HERC4	OTUD5	MCM9	CTNNBL1	TDP2	RNASEH2C
KCTD8	JMJD6	PAWR	MCPH1	DBF4	TMPRSS9	RRM1
KDM8	LIN37	PKMYT1	MECR	DHX9	TOP1	RRP12
LCT	MCM6	RBM38	MUS81	DNTTIP1	UBE2C	RTN4RL1
MAD2L2	NAA25	RNASEH2A	NAA30	ESCO2	ZNF451	S1PR1
MYH2	NAA50	RNASEH2B	NAE1	GIGYF2	APEX2	SHFM1
NBN	PPP4C	SETD2	NPEPPS	MAU2	C11orf30	SUPT20H
NSMF	RPRD2	SKA2	OXSM	NARG2	C5orf22	THAP7
PALB2	SIRT6	SLC29A3	PAXIP1	PSMB6	ADH5	TLK2
PHIP	SRSF6	SNAI2	PDS5B	RAD51AP1	ARID4B	TRNAU1AP
PPP2R4	TAF3	TAOK1	PGD	RBBP8	BRMS1	USP19
PPP6C	TDP1	THOC6	PPP1R8	RMI1	CALM3	USP37
RAD1	TRIM33	TM2D2	PTBP1	RNF8	CDH3	WEE1
RAD51B	ZC3H7A	TOPBP1	PTEN	SART1	CLDN9	YWHAE
RAD51C	ZFYVE19	UBA5	RAD17	SFR1	DGAT2L6	MSH2
REV3L	ANAPC2	UBR5	RAD51D	SRSF11	ESD	MSH6
RFWD3	GATAD2A	ACTA2	RTEL1	SUZ12	GOT1	ATAD5
RMI2	KRT74	ANAPC15	SIVA1	SYNE1	HSD17B12	ATXN7L3

SULT1E1	MFSD2B	ASUN	SLX4	TPI1	IRX6	C1orf86
TMPRSS13	NACC2	ATP2C1	STRA13	WDHD1	METAP2	CCNF
TRAIP	TADA2B	BTAF1	TAF13	ACTG1	OR2T4	CDK2
UBE2M	UBR4	C16orf80	TIPRL	APEX1	RPS23	CELF1
UBE2T	ZNF428	CCS	TXNDC17	ARFRP1	SDC4	CHAF1A
UVSSA	ATP6V1D	COPZ1	USP1	ARHGAP29	SFRP5	CHERP
XPA	BMPR2	CXorf56	USP9X	ATMIN	SRR	CTSH
XRCC2	BRD4	DHX35	VWA9	ATP1A1	THUMPD2	EAPP
XRCC3	CENPC	DNAJC8	WDR48	BABAM1	TIAL1	FBXO11
ZBTB17	DDRGK1	GPATCH1	ZFAT	BRE	XPR1	FBXW7
ASNA1	EPT1	HSF1	DOHH	C15orf56	ZC2HC1C	FEN1
ATP6AP1	FAM180A	HSPA8	MED12	C16orf72	ZNF385D	GTF2F1
ATP6AP2	FNDC3B	HSPB3	AMBRA1	C20orf196	DIS3	KPNA2
MBTPS1	GTF2H1	IL6ST	AXIN1	CLUH	DKC1	LRWD1
MBTPS2	IFI30	LIME1	BCL2L1	COPS4	GTF2H4	NXT1
SLC12A9	ITGB1BP1	LRRC23	CBFB	CTC-534A2.:	MOCS3	ORC2
WDR7	KIAA1432	OSTC	CEBPB	DCLRE1C	RCL1	ORC6
WRB	KRIT1	PPIE	CPEB4	DDI2	WDR55	PAGR1
ACTB	LDLR	SF3A2	CSNK1A1	DUT	PTGR1	PIAS1
ATG9A	MAP2K7	SLC31A1	ELMSAN1	ELL	BBX	POLH
BAP1	NUDT6	SNRPA	FRYL	ERCC6L2	CKAP2	PRPF39
C12orf44	PIGW	SNRPB2	HMGCR	FAM175A	DNM2	RNF111
C7orf55-LU(	PIH1D1	STIP1	HMGCS1	FAM35A	ERCC3	RPRD1B
CAB39	PSTK	SUPT3H	MAP7D1	FIGNL1	GNA13	SENP1
CAND1	PUF60	SYNGR2	NF2	GATAD1	LIG1	SERF2
CAPZA1	RPL36	TADA1	RB1	GLRX3	MARK2	TAF8
CCM2	UFM1	TMED10	TSC1	H2AFX	MED1	TGFBR2
CCNC	VPS41	TMED2	TSC2	HMCES	MED16	TMOD3
CDYL	ZNHIT3	UBA3	VPS37A	IREB2	OTUB1	AGER
CIRH1A	ZWINT	USP14	C15orf41	KANSL2	PAN2	CNOT10
CNBP	SETDB1	WDR83	ACTR1A	KIF18A	RIC8A	FOXN3
CRYBA4	ATXN7L3B	ATR	ANKRD17	LIG4	SAMD4B	ISG20L2
CSNK2A2	CNOT3	C1orf27	ARIH1	MDC1	SEPHS1	ALDH1A1
DHX36	DCUN1D5	C5orf30	ARMC5	MMS19	SKA1	PRH2
DMAP1	KLF6	CBX1	ATRIP	MRE11A	SLBP	ULK2
EPC1	KMT2D	CDC25C	B4GALT7	MRTO4	SMARCD1	AR
FAM49B	KRTAP25-1	CNOT2	BBC3	NCOA4	SNAP29	ARID2
GABPB1	LAMTOR5	CYLD	BRD9	NDST1	SPTLC1	BMX
H2AFZ	LARP7	DDX11	CLASRP	NHEJ1	SPTLC2	CDCA2
HRAS	OR51J1	ERCC5	CREB1	NPLOC4	STAG2	EXTL3
IRF2	RABGEF1	EXO1	CRYBA2	NSUN5	TAF11	HAUS5
KIR3DL1	RBPJ	HSP90AB1	CTDSPL2	PARP1	TMEM30A	KIF4A
KRTAP10-3	RECQL5	HSPA4	CXXC1	PNKP	TRAF2	KIRREL
LRRC71	RFC3	LMNB1	CYP3A43	POLE3	TRAF3	LMNA
MAEA	RLF	POLK	DCP2	POLE4	TRIM28	NAP1L4
MAPK1	RRAGA	PRIMPOL	DDX42	POLL	UBE2G1	NFKBIA
MEMO1	RRM2	RAD18	FBXW11	POLQ	UFC1	NPC1
CYFIP1	PTPN14	GPI	DOT1L	RD3L	GSG2	PFN1

DOLPP1	RALGAPB	GRB2	DYRK1A	RNF146	GZF1	PPP2CA
RHOA	SPNS2	MATR3	EXT2	RB1CC1	IER2	DAZAP1
ZNF827	STK11IP	MCTS1	F8	SAMD1	KIAA0907	DDX5
ABCG2	TAOK2	MPHOSPH8	FBXO32	RAD9A	LATS2	DENR
ADPGK	TGIF2	MSL1	FXR1	REV1	SECISBP2L	DNAJA2
ADSL	THOC1	MVD	GCA	RPS6KA6	SLFN11	DTYMK
ADSS	TIGD3	NAPG	GDPD2	SCAI	PRTN3	DYNC1LI2
AHSA1	TRIM35	NEK2	GMFB	SEMA4C	RLIM	E2F3
ANAPC13	UBE3A	NSD1	GNAS	N6AMT1	RRAGC	EIF4E2
AP2B1	ZDHHC9	PBRM1	HMCN1	SLC25A37	SCAP	EP300
ARL1	ZPBP2	PHF10	HOXB7	SLC35B2	SCN11A	FAM122A
ASH2L	MLH1	PIP5K1A	HTATSF1	SLC39A9	TMEM221	FBXO42
ATP13A1	PMS2	PMVK	HTR3D	SEPSECS	VPS16	FCHO2
BCAP31	ACTR5	PPP1CA	IKBKB	SIN3B	VPS33A	FITM2
BRD1	ATP6V0C	PPP1CC	JAK3	SOX9	VPS39	FKBP8
BRD7	C1orf85	PRDX1	KCNK3	SRSF12	ZNF823	FOXP1
C9orf41	CCDC130	PSME3	KCTD10	SUPT7L	ARPC4	GET4
CAP1	CDC6	RANBP3	KLHL4	TMEM233	ASF1A	GID8
CCDC6	DCPS	RAVER1	LRRC28	UGP2	BEND3	EPB41L2
CCNB1	FAM135B	RBM10	MAGEA1	VAC14	CCDC101	EXT1
CDC25A	FAM205A	RNF214	NMUR2	ZNF766	SCNM1	MIB1
CDCA5	FLCN	SACS	PATL1	GLMN	SLC25A28	MNS1
CENPO	HDAC7	SGOL1	PPM1F	HAUS3	PIGF	PCGF1
CFL1	HNRNPU	SGOL2	PPP1R3B	HNRNPLL	PTPN12	PDCD10
CLASP2	LAMTOR2	SLC10A7	PPP2R2A	HUWE1	RAD54L2	PDS5A
CLK3	LAMTOR3	SMARCA4	VARS2	KCTD11	WDR20	PUM1
CORO1C	MMGT1	SMG7	WNK1	KDM1A	ABL1	RAB18
CUL1	NASP	SP3	ADAMTS20	LRRK2	AMOTL2	
CWC27	OLA1	RAD54L	BAZ1B	MAP4K4	APRT	
CAST	CIRBP	RIF1	C2orf70	MTMR12	B3GALT6	
CDRT1	CPZ	RNF168	ENC1	PPIL2	B3GAT3	
CHTF18	CRYBB3	RNF25	RPL28	RAD50		

Subtype by IHC-CXorf56 expression

Normalized read counts		
TNBC	HER2+	ER/PR+
539.382095	692.846604	370.842948
595.540735	720.102136	475.035597
745.333986	777.293805	581.934907
849.428493	813.886531	623.545144
938.385939	837.349231	671.319397
982.83886	862.164079	675.172668
996.905424	896.6716	688.89274
998.435187	902.140281	692.846604
1004.30685	968.531534	720.102136
1027.03835	978.184853	721.421763
1097.98923	1018.291	768.255716
1109.54454	1023.15785	777.293805
1193.50271	1047.11977	805.872399
1194.50246	1050.46809	807.309266
1206.6174	1063.31739	813.055554
1297.36411	1082.16559	816.596535
1307.41585	1110.52852	820.183245
1316.54274	1123.3308	837.349231
1328.94449	1128.0254	843.666978
1330.54462	1144.09547	848.028268
1332.42561	1145.42432	848.639151
1334.84168	1165.57286	850.599464
1337.77654	1170.15885	852.61941
1349.88936	1175.75378	855.102483
1351.48453	1195.03893	862.164079
1355.98734	1206.77328	862.782492
1382.07289	1218.48664	866.356055
1383.90456	1237.24521	895.607628
1390.64452	1262.891	896.6716
1412.32284	1266.63734	902.140281
1436.37502	1276.33867	920.832903
1443.15235	1290.75469	925.081959
1456.05166	1324.22607	926.105707
1462.95096	1325.94145	928.751648
1501.66799	1332.85544	931.991838
1540.27794	1333.20042	932.997908
1543.29268	1339.50822	936.533454
1580.82504	1342.63316	938.005543
1584.76121	1349.30777	941.818407
1597.24575	1354.98077	945.16369
1602.30861	1356.42012	948.951509
1635.60879	1359.09494	951.484409
1640.80478	1370.17109	957.916477
1646.93378	1370.72516	957.982499
1648.4895	1372.10778	959.26788
1651.80784	1376.40434	962.30703
1669.74623	1393.72119	964.279145
1674.27415	1407.49442	967.937649
1674.35716	1426.51724	969.896646
1681.36861	1427.01818	973.583139

Averraged normalized read counts	
TNBC	non-TNBC
1953.460979	1510.029621

Normalized read counts		
TNBC	HER2+	ER/PR+
1684.94495	1428.87406	978.184853
1685.06451	1447.45931	980.370991
1695.92871	1456.17496	984.198381
1711.28273	1462.57823	989.088375
1718.7829	1472.04247	990.583356
1729.83606	1483.85553	1015.79834
1730.86634	1485.57672	1018.291
1732.86329	1494.09887	1023.15785
1740.27497	1495.00268	1025.42159
1756.4756	1495.21394	1036.91835
1760.3456	1510.17937	1042.24491
1774.36893	1514.75233	1043.88958
1803.79558	1532.99684	1047.11977
1810.99155	1534.44916	1049.37622
1861.25952	1541.57828	1050.1868
1871.43312	1552.43975	1050.22631
1885.52715	1552.77268	1054.85514
1891.39168	1602.76704	1061.31891
1895.31827	1639.13456	1063.31739
1919.1878	1644.3962	1064.31752
1930.10838	1682.62013	1065.44392
1951.58857	1705.02243	1066.72541
1951.63022	1714.19674	1073.57614
1964.52475	1715.93143	1077.17621
1964.88691	1719.84888	1082.16559
1978.8142	1721.70843	1083.89156
1982.68611	1724.20085	1086.98783
2016.13158	1739.49179	1087.90391
2050.4883	1758.11034	1088.57547
2057.29744	1779.91416	1088.94892
2061.77085	1784.49396	1089.72373
2065.5643	1817.09571	1092.92365
2089.94055	1821.11409	1096.81636
2097.23445	1830.01178	1105.06397
2123.3022	1846.06408	1106.5737
2136.51914	1882.94953	1108.95668
2192.40458	1935.79628	1110.52852
2218.38809	1966.32541	1114.18558
2218.76192	1969.67241	1115.36491
2227.76403	1983.8188	1123.3308
2241.17174	2006.63408	1125.25946
2338.46743	2038.08575	1127.81166
2355.32835	2073.68551	1128.0254
2379.40012	2140.06723	1128.25803
2400.45463	2148.9023	1130.00791
2409.0211	2160.22524	1132.94658
2415.87685	2175.47883	1134.33334
2435.9075	2261.29381	1134.99776
2443.48013	2261.86259	1135.27339
2462.20422	2266.84167	1137.43856

Normalized read counts		
TNBC	HER2+	ER/PR+
2485.40324	2297.4675	1140.04042
2516.30898	2346.05001	1145.42432
2560.90315	2392.96089	1147.99079
2571.21151	2406.42741	1149.36907
2656.4697	2440.07137	1151.43078
2671.0028	2756.68098	1152.07782
2683.81789	2974.88432	1152.21666
2748.55065	3763.53004	1153.43855
2828.39137		1154.71892
2831.15753		1156.34396
2848.73596		1159.47898
2852.80979		1162.85525
2941.47397		1163.77198
2949.46238		1165.57286
2969.79805		1168.1256
3047.41306		1170.15885
3458.75963		1170.60206
3688.86011		1173.53119
4126.62442		1173.70732
4491.74911		1175.20096
4693.96505		1175.75378
6514.09924		1179.83772
		1181.0188
		1181.89425
		1182.38142
		1184.18802
		1188.09169
		1193.15844
		1194.87688
		1195.03893
		1195.08533
		1195.41791
		1195.67446
		1196.97767
		1197.52614
		1199.38455
		1199.48437
		1204.43082
		1206.06329
		1206.77328
		1208.60404
		1212.98706
		1213.17277
		1216.19025
		1216.52834
		1216.558
		1216.76706
		1218.48664
		1220.06734
		1224.08932

Normalized read counts		
TNBC	HER2+	ER/PR+
		1224.35598
		1224.52943
		1226.10515
		1228.02424
		1229.0422
		1229.75989
		1231.04712
		1232.98788
		1235.88294
		1235.98964
		1237.24521
		1238.15276
		1238.79799
		1241.49997
		1244.28639
		1245.86823
		1249.23912
		1250.41966
		1250.61631
		1252.06803
		1253.48014
		1254.46453
		1255.65814
		1259.80463
		1261.98086
		1262.891
		1263.89921
		1265.35594
		1266.63734
		1272.8329
		1274.83864
		1275.79469
		1276.33867
		1277.15562
		1277.7161
		1283.78007
		1284.95779
		1287.74696
		1288.46245
		1289.18489
		1289.93085
		1289.98537
		1290.75469
		1291.13593
		1291.52818
		1292.29258
		1293.01809
		1293.7116
		1297.46453
		1299.4352

Normalized read counts		
TNBC	HER2+	ER/PR+
		1299.73555
		1300.08881
		1302.08518
		1303.23277
		1303.39337
		1303.61554
		1304.73421
		1305.72839
		1307.07335
		1312.06345
		1313.33251
		1313.47547
		1314.32479
		1315.68045
		1318.8914
		1319.35534
		1321.01805
		1323.09559
		1323.6113
		1323.74538
		1324.22607
		1325.94145
		1327.97317
		1330.75899
		1330.83221
		1332.40331
		1332.85544
		1333.13733
		1333.20042
		1333.26937
		1333.70643
		1333.88737
		1338.1778
		1339.50822
		1340.37837
		1340.6288
		1341.00519
		1341.88606
		1342.00045
		1343.36474
		1344.16049
		1345.78731
		1348.26753
		1349.30777
		1350.01368
		1354.98077
		1355.51951
		1356.10085
		1356.42012
		1359.09135

Normalized read counts		
TNBC	HER2+	ER/PR+
		1362.14762
		1363.07386
		1365.63282
		1366.28915
		1366.84543
		1367.29547
		1368.27331
		1369.2726
		1371.49823
		1372.10778
		1372.81061
		1376.40434
		1377.09666
		1377.45543
		1377.63406
		1378.23604
		1382.1357
		1382.17201
		1382.64918
		1383.87248
		1389.22267
		1391.31079
		1392.9814
		1395.2097
		1398.87808
		1401.22644
		1401.36252
		1402.13055
		1404.21851
		1404.51606
		1405.50863
		1407.49442
		1408.12484
		1409.42523
		1410.2249
		1414.19776
		1415.8321
		1416.61802
		1416.81934
		1416.97439
		1417.28944
		1419.06529
		1419.47031
		1420.20024
		1420.45528
		1422.30911
		1422.45927
		1422.56778
		1427.98756
		1428.04631

Normalized read counts		
TNBC	HER2+	ER/PR+
		1432.29303
		1434.07072
		1434.32137
		1434.58155
		1438.52577
		1439.17543
		1439.74854
		1441.86574
		1442.40617
		1442.68862
		1443.46773
		1445.11931
		1445.97312
		1447.45931
		1451.93553
		1452.35485
		1454.3912
		1456.17496
		1458.483
		1458.90375
		1459.01827
		1459.06839
		1460.23932
		1460.93724
		1461.06154
		1461.84346
		1464.44851
		1466.0603
		1468.51415
		1469.22532
		1469.78795
		1471.37318
		1471.38871
		1472.04247
		1473.45376
		1474.50184
		1475.88197
		1475.97649
		1479.5764
		1481.58872
		1485.15208
		1485.57672
		1486.00167
		1486.23624
		1486.89041
		1488.22808
		1490.93721
		1491.3892
		1492.08629
		1493.32474

Normalized read counts		
TNBC	HER2+	ER/PR+
		1493.62184
		1494.40495
		1495.21394
		1500.67662
		1502.32099
		1504.69175
		1510.17937
		1512.40549
		1512.66019
		1514.75233
		1517.16661
		1519.83606
		1520.4081
		1523.77156
		1525.80692
		1526.80589
		1527.04695
		1528.4986
		1530.04932
		1533.13484
		1533.30841
		1533.78927
		1534.44916
		1537.01705
		1537.07955
		1542.55977
		1543.0619
		1544.4316
		1544.87164
		1550.26523
		1551.53744
		1552.36784
		1552.43975
		1552.77268
		1558.68094
		1561.11347
		1561.21143
		1562.10393
		1562.73766
		1564.01575
		1566.2027
		1567.26653
		1568.3528
		1572.8627
		1574.16712
		1576.45525
		1578.93704
		1581.36483
		1581.38929
		1583.26525

Normalized read counts		
TNBC	HER2+	ER/PR+
		1584.56177
		1584.6235
		1586.62144
		1587.65854
		1589.2551
		1591.21597
		1598.90544
		1601.15435
		1602.52205
		1602.76704
		1604.14805
		1607.72474
		1609.725
		1609.84786
		1611.05012
		1611.08437
		1616.17931
		1619.71101
		1620.39631
		1623.65893
		1627.96008
		1629.18042
		1630.47713
		1630.75759
		1631.4579
		1631.67245
		1633.00623
		1640.68433
		1641.8146
		1641.88304
		1642.05201
		1645.04619
		1655.33986
		1655.5938
		1657.15762
		1660.70335
		1664.63803
		1666.60264
		1667.71349
		1668.65388
		1668.78204
		1672.79648
		1672.89593
		1679.08512
		1681.17716
		1682.57184
		1685.72143
		1689.05651
		1691.72653
		1700.88866

Normalized read counts		
TNBC	HER2+	ER/PR+
		1701.30543
		1705.89561
		1710.5134
		1713.45389
		1714.19674
		1715.8862
		1715.93143
		1719.84888
		1721.40096
		1721.70843
		1724.20085
		1728.51946
		1732.6121
		1734.34833
		1735.51369
		1739.49179
		1742.27037
		1743.89698
		1746.22124
		1762.46034
		1764.48418
		1764.55903
		1769.08736
		1772.16244
		1778.22125
		1779.91416
		1784.49396
		1786.99919
		1789.24745
		1789.51047
		1790.92282
		1794.54834
		1795.21465
		1797.63076
		1797.99688
		1800.78149
		1801.52651
		1802.64605
		1813.66004
		1815.29643
		1817.09571
		1821.11409
		1824.09423
		1830.01178
		1834.84192
		1844.52193
		1845.35094
		1852.88602
		1860.73512
		1866.11137

Normalized read counts		
TNBC	HER2+	ER/PR+
		1870.05455
		1878.89539
		1889.19442
		1896.41527
		1899.49504
		1901.70985
		1911.26582
		1915.34662
		1915.554
		1918.89141
		1920.83001
		1925.5936
		1929.04529
		1935.79628
		1935.83652
		1941.12675
		1943.27765
		1943.40747
		1945.57766
		1946.91292
		1947.3305
		1952.3676
		1960.03823
		1960.80305
		1962.318
		1969.67241
		1981.75809
		1983.8188
		1992.81261
		1995.2007
		2006.63408
		2008.69548
		2009.64453
		2019.02044
		2038.08575
		2044.28055
		2055.39568
		2057.07908
		2059.85732
		2071.42478
		2075.56742
		2075.62667
		2081.89826
		2090.66571
		2091.74515
		2110.65962
		2122.87381
		2128.05018
		2140.06723
		2145.55472

Normalized read counts		
TNBC	HER2+	ER/PR+
		2148.69901
		2148.9023
		2153.37981
		2160.22524
		2175.47883
		2186.67708
		2188.74186
		2194.30551
		2203.74232
		2213.99385
		2224.96465
		2250.36477
		2261.29381
		2261.4819
		2261.86259
		2284.31488
		2285.84498
		2307.02496
		2309.49809
		2321.0374
		2329.6371
		2346.05001
		2348.60356
		2355.62007
		2377.1036
		2378.20665
		2382.3021
		2386.63323
		2392.96089
		2406.42741
		2408.32446
		2425.93406
		2445.34819
		2475.09635
		2516.5823
		2539.56239
		2542.87654
		2561.0549
		2566.64748
		2612.40271
		2621.04939
		2647.79924
		2649.55675
		2653.4473
		2947.32062
		3049.25076
		3171.78245
		3410.91293
		3549.73388
		3757.60315

Normalized read counts		
TNBC	HER2+	ER/PR+
		4553.4811

Subtype by PAM50-CXorf56 expression

Normalized read counts				
Basal-like	HER2+	Luminal A	Luminal B	Normal-like
745.333986	720.102136	623.545144	581.934907	688.89274
849.428493	813.886531	671.319397	692.846604	1088.57547
996.905424	837.349231	675.172668	848.028268	1355.98734
998.435187	843.666978	768.255716	848.639151	1340.37837
1004.30685	850.599464	807.309266	855.102483	1382.64918
1027.03835	862.164079	813.055554	902.140281	1389.22267
1097.98923	896.6716	816.596535	932.997908	1540.27794
1066.72541	964.279145	852.61941	936.533454	1488.22808
1193.50271	968.531534	982.83886	951.484409	
1181.0188	1043.88958	920.832903	989.088375	
1297.36411	1050.46809	925.081959	1015.79834	
1206.77328	1123.3308	926.105707	1018.291	
1328.94449	1128.0254	931.991838	1050.1868	
1334.84168	1144.09547	938.005543	1050.22631	
1337.77654	1165.57286	941.818407	1082.16559	
1349.88936	1170.15885	945.16369	1088.94892	
1283.78007	1193.15844	948.951509	1137.43856	
1382.07289	1195.03893	957.982499	1152.07782	
1383.90456	1229.0422	962.30703	1204.43082	
1332.40331	1237.24521	967.937649	1208.60404	
1456.05166	1303.39337	973.583139	1213.17277	
1362.14762	1330.54462	980.370991	1216.558	
1462.95096	1332.85544	984.198381	1224.08932	
1445.97312	1339.50822	990.583356	1265.35594	
1456.17496	1349.30777	1023.15785	1275.79469	
1580.82504	1359.09494	1025.42159	1293.7116	
1584.76121	1393.72119	1042.24491	1299.73555	
1493.62184	1426.51724	1047.11977	1302.08518	
1592.00497	1427.01818	1061.31891	1304.73421	
1597.24575	1428.87406	1063.31739	1312.06345	
1519.83606	1462.57823	1064.31752	1324.22607	
1635.60879	1483.85553	1073.57614	1333.26937	
1640.80478	1495.00268	1077.17621	1366.28915	
1646.93378	1602.30861	1083.89156	1377.45543	
1674.27415	1651.80784	1087.90391	1382.17201	
1681.36861	1602.52205	1089.72373	1402.13055	
1684.94495	1682.62013	1105.06397	1404.21851	
1685.06451	1705.02243	1106.5737	1405.50863	
1695.92871	1721.70843	1108.95668	1422.30911	
1711.28273	1758.11034	1125.25946	1422.45927	
1729.83606	1762.46034	1127.81166	1428.04631	
1737.43498	1779.91416	1128.25803	1432.29303	
1740.27497	1794.54834	1130.00791	1438.52577	
1760.3456	1817.09571	1134.33334	1439.74854	
1668.78204	1830.01178	1134.99776	1460.93724	
1803.79558	1846.06408	1135.27339	1469.78795	
1810.99155	1978.8142	1145.42432	1485.57672	
1764.55903	1918.89141	1147.99079	1486.23624	
1861.25952	1983.8188	1153.43855	1486.89041	
1871.43312	1995.2007	1154.71892	1491.3892	

Averraged normalized read counts	
TNBC	non-TNBC
2014.28947	1502.364699

Normalized read counts				
Basal-like	HER2+	Luminal A	Luminal B	Normal-like
1919.1878	2008.69548	1156.34396	1493.32474	
1930.10838	2203.74232	1163.77198	1494.40495	
1946.43667	2261.29381	1168.1256	1510.17937	
1951.58857	2297.4675	1170.60206	1523.77156	
1951.63022	2462.20422	1173.53119	1526.80589	
1964.52475	2756.68098	1179.83772	1527.04695	
1964.88691	2974.88432	1182.38142	1533.30841	
2016.13158	3763.53004	1184.18802	1550.26523	
2050.4883		1194.87688	1551.53744	
2057.29744		1195.08533	1552.77268	
1962.318		1195.41791	1561.21143	
2057.48241		1196.97767	1576.45525	
2065.5643		1197.52614	1581.36483	
2089.94055		1199.38455	1584.6235	
2097.23445		1199.48437	1587.65854	
2123.3022		1206.06329	1602.76704	
2136.51914		1216.19025	1611.05012	
2192.40458		1216.52834	1619.71101	
2218.38809		1216.76706	1620.39631	
2218.76192		1220.06734	1640.68433	
2128.05018		1224.52943	1641.8146	
2227.76403		1228.02424	1642.05201	
2241.17174		1232.98788	1664.63803	
2290.61892		1241.49997	1668.65388	
2355.32835		1244.28639	1689.05651	
2409.0211		1252.06803	1701.30543	
2443.48013		1254.46453	1724.20085	
2485.40324		1261.98086	1732.6121	
2516.30898		1266.63734	1764.48418	
2560.90315		1272.8329	1769.08736	
2656.4697		1277.15562	1778.22125	
2566.64748		1284.95779	1786.99919	
2671.0028		1287.74696	1789.24745	
2683.81789		1288.46245	1885.52715	
2748.55065		1289.93085	1801.52651	
2828.39137		1289.98537	1852.88602	
2831.15753		1290.75469	1866.11137	
2852.80979		1291.13593	1878.89539	
2949.46238		1292.29258	1925.5936	
2969.79805		1300.08881	1935.79628	
3047.41306		1307.07335	1952.3676	
3458.75963		1313.33251	1960.80305	
3541.30633		1313.47547	1969.67241	
4491.74911		1314.32479	2006.63408	
4553.4811		1412.32284	2009.64453	
4693.96505		1319.35534	2044.28055	
6514.09924		1321.01805	2055.39568	
		1323.09559	2075.56742	
		1323.6113	2140.06723	
		1323.74538	2148.69901	

Normalized read counts				
Basal-like	HER2+	Luminal A	Luminal B	Normal-like
		1325.94145	2175.47883	
		1330.75899	2188.74186	
		1330.83221	2194.30551	
		1333.20042	2213.99385	
		1333.88737	2224.96465	
		1338.1778	2261.86259	
		1340.6288	2307.02496	
		1341.00519	2346.05001	
		1341.88606	2348.60356	
		1342.00045	2355.62007	
		1343.36474	2386.63323	
		1344.16049	2408.32446	
		1443.26131	2425.93406	
		1350.01368	2445.34819	
		1354.98077	2475.09635	
		1359.09135	2539.56239	
		1363.07386	2542.87654	
		1366.84543	2649.55675	
		1367.29547	2947.32062	
		1368.27331	3410.91293	
		1369.2726		
		1372.10778		
		1372.81061		
		1376.40434		
		1377.09666		
		1377.63406		
		1378.23604		
		1391.31079		
		1401.36252		
		1404.51606		
		1408.12484		
		1414.19776		
		1416.81934		
		1416.97439		
		1417.28944		
		1420.20024		
		1422.56778		
		1427.98756		
		1434.07072		
		1434.32137		
		1434.58155		
		1439.17543		
		1442.68862		
		1452.35485		
		1454.3912		
		1458.90375		
		1459.01827		
		1459.06839		
		1460.23932		
		1464.44851		

Normalized read counts				
Basal-like	HER2+	Luminal A	Luminal B	Normal-like
		1466.0603		
		1563.83766		
		1471.38871		
		1475.88197		
		1479.5764		
		1481.58872		
		1490.93721		
		1502.32099		
		1512.40549		
		1525.80692		
		1537.01705		
		1537.07955		
		1542.55977		
		1552.36784		
		1562.10393		
		1566.2027		
		1574.16712		
		1581.38929		
		1584.56177		
		1589.2551		
		1604.14805		
		1607.72474		
		1609.725		
		1609.84786		
		1616.17931		
		1623.65893		
		1630.47713		
		1630.75759		
		1631.4579		
		1633.00623		
		1645.04619		
		1657.15762		
		1660.70335		
		1667.71349		
		1672.79648		
		1682.57184		
		1691.72653		
		1705.89561		
		1713.45389		
		1714.19674		
		1728.51946		
		1735.51369		
		1739.49179		
		1743.89698		
		1746.22124		
		1789.51047		
		1790.92282		
		1802.64605		
		1815.29643		
		1834.84192		

Normalized read counts				
Basal-like	HER2+	Luminal A	Luminal B	Normal-like
		1845.35094		
		1860.73512		
		1870.05455		
		1889.19442		
		1896.41527		
		1899.49504		
		1901.70985		
		1929.04529		
		1945.57766		
		1946.91292		
		1947.3305		
		1960.03823		
		1981.75809		
		2019.02044		
		2075.62667		
		2091.74515		
		2110.65962		
		2122.87381		
		2186.67708		
		2250.36477		
		2415.87685		
		2435.9075		
		2377.1036		
		2378.20665		
		2528.41266		
		2612.40271		
		2647.79924		
		2653.4473		
		3757.60315		
