# Supplementary material for: Inhibition of CXorf56 promotes PARP inhibitor-induced cytotoxicity in triple-negative breast cancer
Source: NPJ Breast Cancer. 2023 May 8;9:34. doi: 10.1038/s41523-023-00540-3 (PMC10167262; doi:10.1038/s41523-023-00540-3)
Supplement: Supplementary file 2 — Reporting Summary [file 41523_2023_540_MOESM2_ESM.pdf]

Corresponding author(s): Jifeng Feng, Hongxia Ma, Wei Bao

Last updated by author(s): Mar 21, 2023

Reporting Summary

Nature Portfolio wishes to improve the reproducibility of the work that we publish. This form provides structure for consistency and transparency in reporting. For further information on Nature Portfolio policies, see our Editorial Policies and the Editorial Policy Checklist.

Statistics

For all statistical analyses, confirm that the following items are present in the figure legend, table legend, main text, or Methods section.

n/a	Confirmed	
☐	☒	The exact sample size (n) for each experimental group/condition, given as a discrete number and unit of measurement
☐	☒	A statement on whether measurements were taken from distinct samples or whether the same sample was measured repeatedly
☐	☒	The statistical test(s) used AND whether they are one- or two-sided *Only common tests should be described solely by name; describe more complex techniques in the Methods section.*
☐	☒	A description of all covariates tested
☐	☒	A description of any assumptions or corrections, such as tests of normality and adjustment for multiple comparisons
☐	☒	A full description of the statistical parameters including central tendency (e.g. means) or other basic estimates (e.g. regression coefficient) AND variation (e.g. standard deviation) or associated estimates of uncertainty (e.g. confidence intervals)
☐	☒	For null hypothesis testing, the test statistic (e.g. F, t, r) with confidence intervals, effect sizes, degrees of freedom and P value noted *Give P values as exact values whenever suitable.*
☒	☐	For Bayesian analysis, information on the choice of priors and Markov chain Monte Carlo settings
☒	☐	For hierarchical and complex designs, identification of the appropriate level for tests and full reporting of outcomes
☐	☒	Estimates of effect sizes (e.g. Cohen's d, Pearson's r), indicating how they were calculated

Our web collection on statistics for biologists contains articles on many of the points above.

Software and code

Policy information about availability of computer code

Data collection	-
Data analysis	The number of the foci was quantified using ImageJ software v1.8.0 (National Institutes of Health, Bethesda, MD, USA).<br>Level-3 RNA sequencing data were downloaded from TCGA (https://portal.gdc.cancer.gov/).<br>Top 500 genes related to OS from breast cancer patients were obtained by searching the 'Most Differential Survival Genes' section of the GEPIA2 website.

For manuscripts utilizing custom algorithms or software that are central to the research but not yet described in published literature, software must be made available to editors and reviewers. We strongly encourage code deposition in a community repository (e.g. GitHub). See the Nature Portfolio guidelines for submitting code & software for further information.

Materials & experimental systems

n/a	Involved in the study
☐	☒ Antibodies
☐	☒ Eukaryotic cell lines
☐	☐ Palaeontology and archaeology
☐	☒ Animals and other organisms
☐	☒ Clinical data
☒	☐ Dual use research of concern

Methods

n/a	Involved in the study
☒	☐ ChIP-seq
☒	☐ Flow cytometry
☒	☐ MRI-based neuroimaging

Antibodies

Antibodies used	CXorf56 (PA5-58310, Thermo Fisher Scientific, Waltham, MA, USA);<br>ER (PA1-311, Thermo Fisher Scientific);<br>PR (MA1-411, Thermo Fisher Scientific),<br>Her2 (MA5-13105, Thermo Fisher Scientific),<br>γ-H2AX (ab229914, Abcam),<br>Ku70 (ab92450, Abcam) ,<br>MDC1(ab271061, Abcam)<br>53BP1 (ab87097, Abcam)<br>DNA-PKcs (ab44815, Abcam)<br>LIG4 (ab232658, Abcam)<br>XRCC4 (ab213729, Abcam)<br>BRCA2 (PA5-105731, Thermo Fisher Scientific, Waltham, MA, USA);<br>CXorf56 (24021-1-AP, Proteintech),<br>Ku70 (ab92450, Abcam, Cambridge, UK),<br>FLAG (ab205606, Abcam),<br>β-actin (sc-81178, Santa Cruz Biotechnology, Dallas, TX, USA).
Validation	CXorf56 (PA5-58310, WB and IP, Rabbit polyclonal antibody),<br>ER (PA1-311, WB, IF, IHC, ICC/IF and GS, Rabbit polyclonal antibody)<br>PR (MA1-411, WB, IHC, ICC/IF, Flow, ELISA, IP, DB and GS, Mouse monoclonal antibody),<br>Her2 (MA5-13105, Thermo Fisher Scientific),<br>γ-H2AX (ab229914, PepArr, ChIP, ICC/IF, Flow Cyt (Intra), WB, IHC-P and IP, Rabbit monoclonal antibody),<br>Ku70 (ab92450, WB, IP, IHC-P, ICC/IF and Flow Cyt (Intra), Rabbit monoclonal antibody) ,<br>MDC1(ab271061, Flow Cyt, ICC/IF, IHC-P and WB, Rabbit monoclonal antibody)<br>53BP1 (ab87097, CC/IF, IHC-P, IP and WB, Rabbit polyclonal antibody)<br>DNA-PKcs (ab44815, ICC/IF and WB, Mouse monoclonal antibody)<br>LIG4 (ab232658, ICC/IF, IHC-P and WB, Rabbit monoclonal antibody)<br>XRCC4 (ab213729, ICC/IF, IHC-P and WB, Rabbit polyclonal antibody)<br>BRCA2 (PA5-105731, WB and ICC/IF, Rabbit Polyclonal Antibody)<br>CXorf56 (24021-1-AP, IF, IP, WB,ELISA, Rabbit Polyclonal Antibody),<br>Ku70 (ab92450, WB, IP, IHC-P, ICC/IF and Flow Cyt (Intra), Rabbit monoclonal antibody),<br>FLAG (ab205606, WB, ICC/IF, Flow Cyt, IHC-P and IP, Rabbit monoclonal antibody),<br>β-actin (sc-81178, WB, IP, IF and FCM, Mouse monoclonal antibody)

Eukaryotic cell lines

Policy information about cell lines and Sex and Gender in Research

Cell line source(s)	Breast cancer cell lines (MDA-MB-231, BT549, SUM1315) were obtained from the American Type Culture Collection (http://www.atcc.org). Cell lines were authenticated twice using morphological and isoenzyme analyses
Authentication	The Cell lines have been authenticated based on morphological criteria.
Mycoplasma contamination	All cell lines were tested negative for mycoplasma.
Commonly misidentified lines (See ICLAC register)	-

Palaeontology and Archaeology

Specimen provenance	-
Specimen deposition	-

Dating methods	-

☐ Tick this box to confirm that the raw and calibrated dates are available in the paper or in Supplementary Information.

Ethics oversight	-

Note that full information on the approval of the study protocol must also be provided in the manuscript.

Animals and other research organisms

Policy information about studies involving animals; ARRIVE guidelines recommended for reporting animal research, and Sex and Gender in Research

Laboratory animals	5-week-old female BALB/C nude mice
Wild animals	The study did not involve wild animals.
Reporting on sex	All 48 nude mice are female.
Field-collected samples	The study did not involve samples collected from the field.
Ethics oversight	All mouse experiments were approved by the Laboratory Animal Ethics Committee of Nanjing Medical University and conformed to the legal mandates and national guidelines for the care and maintenance of laboratory animals.

Note that full information on the approval of the study protocol must also be provided in the manuscript.

Clinical data

Policy information about clinical studies

All manuscripts should comply with the ICMJE guidelines for publication of clinical research and a completed CONSORT checklist must be included with all submissions.

Clinical trial registration	-
Study protocol	-
Data collection	-
Outcomes	-
