# Supplementary material for: Inhibition of CXorf56 promotes PARP inhibitor-induced cytotoxicity in triple-negative breast cancer
Source: NPJ Breast Cancer. 2023 May 8;9:34. doi: 10.1038/s41523-023-00540-3 (PMC10167262; doi:10.1038/s41523-023-00540-3)
Supplement: Supplementary file 3 — Supplementary Dataset 1 [file 41523_2023_540_MOESM3_ESM.pdf]

BCAP31 flow records															Z-score			Average Z-score			
			Positive cells (100%)						HR/NHEJ ratio			Fold change of HR/NHEJ ratio									
Cell lines		Group	T1 HR RFP	T1 NHEJ GFP	T2 HR RFP	T2 NHEJ GFP	T3 HR RFP	T3 NHEJ GFP	T1	T2	T3	T1	T2	T3	Avergae	SD		T1	T2	T3	
MDA-MB-231	TNBC	NTC	0.411242	0.027542	0.425457	0.034986	0.545716	0.03981	14.93155	12.16087	13.70802	si/NTC			1.39721	0.40198					
		si1	0.56288	0.026235	0.675001	0.026017	0.671712	0.027055	21.45521	25.94455	24.82729	1.436904	2.133445	1.811151	0.09874	1.83151	1.02974				
		si2	0.432298	0.018529	0.732014	0.035888	0.21861	0.009135	23.33052	20.3972	23.93007	1.562498	1.677281	1.745698	0.41118	0.69672	0.86692				
BT549	TNBC	NTC	0.149051	0.003244	0.243093	0.00583	0.173446	0.004175	45.94604	41.69372	41.54135	si/NTC									
		si1	0.52464	0.010206	0.508503	0.010129	0.541469	0.011913	51.40651	50.20311	45.45056	1.118845	1.204093	1.094104	-0.6925	-0.4804	-0.754	-0.1658	0.0737	0.24825	TNBC
		si2	0.259155	0.00517	0.515108	0.010915	0.438375	0.009376	50.12411	47.19449	46.75303	1.090934	1.131933	1.125457	-0.7619	-0.6599	-0.676				
SUM1315	TNBC	NTC	0.363454	0.041674	0.324764	0.019532	0.285908	0.020035	8.721403	16.62714	14.2702	si/NTC									
		si1	0.403907	0.029759	0.60881	0.030092	0.352803	0.013745	13.57269	20.23151	25.66769	1.556251	1.216776	1.798691	0.39564	-0.4489	0.99875				
		si2	0.205687	0.019362	0.672285	0.033765	0.665005	0.033123	10.62321	19.91084	20.077	1.218062	1.197491	1.406918	-0.4457	-0.4968	0.02415				
T47D	luminal	NTC	0.221665	0.005778	0.282156	0.016987	0.374382	0.020273	38.36124	16.61031	18.46725	si/NTC						T1	T2	T3	
		si1	0.317997	0.008131	0.40303	0.01849	0.372346	0.01731	39.10795	21.79706	21.50993	1.019465	1.312261	1.164761	-0.9397	-0.2113	-0.5783				
		si2	0.536383	0.012835	0.277583	0.010959	0.637326	0.028194	41.79064	25.3296	22.60477	1.089398	1.524933	1.224046	-0.7657	0.31773	-0.4308				
MCF7	luminal	NTC	0.173446	0.005952	0.154054	0.003294	0.198082	0.005544	29.14024	46.76433	35.72607	si/NTC									
		si1	0.449646	0.016982	0.481325	0.010719	0.462592	0.012723	26.47826	44.90586	36.35923	0.908649	0.960259	1.017723	-1.2154	-1.087	-0.944	-0.3167	0.03339	0.12715	luminal
		si2	0.303873	0.011625	0.343042	0.008191	0.305551	0.008137	26.13933	41.87982	37.54981	0.897018	0.895551	1.051048	-1.2443	-1.248	-0.8611				
ZR751	luminal	NTC	0.155782	0.074296	0.29758	0.139027	0.168903	0.080932	2.096759	2.140448	2.086971	si/NTC									
		si1	0.063568	0.014049	0.360511	0.111881	0.014037	0.002767	4.524826	3.222285	5.073924	2.15801	1.505426	2.431239	1.89261	0.2692	2.57232				
		si2	0.317081	0.097767	0.361249	0.074501	0.07752	0.020623	3.243245	4.848911	3.758918	1.546789	2.265373	1.801136	0.3721	2.1597	1.00483				
																		0.15099	0.04031	0.12109	TNBC-luminal (Z-score)
																		0.595275448			P value

CXorf56 flow records															Z-score			Average Z-score			
			Positive cells (100%)						HR/NHEJ ratio			Fold change of HR/NHEJ ratio									
Cell lines		Group	T1 HR RFP	T1 NHEJ GFP	T2 HR RFP	T2 NHEJ GFP	T3 HR RFP	T3 NHEJ GFP	T1	T2	T3	T1	T2	T3	Avergae	SD		T1	T2	T3	
MDA-MB-231	TNBC	NTC	0.41124	0.027542	0.425457	0.0349857	0.54572	0.03981	14.9315	12.1609	13.708	si/NTC			0.20449	0.22565					
		si1	0.00182	0.430927	0.012296	0.5440976	0.00079	0.10626	0.00423	0.0226	0.00742	0.00028	0.00186	0.00054	-0.90496	-0.89798	-0.90382				
		si2	0.00164	0.5006	0.010298	0.5936443	0.00154	0.26799	0.00329	0.01735	0.00575	0.00022	0.00143	0.00042	-0.90524	-0.89989	-0.90436				
BT549	TNBC	NTC	0.14905	0.003244	0.243093	0.0058305	0.17345	0.004175	45.946	41.6937	41.5414	si/NTC									
		si1	0.0255	0.023641	0.112635	0.0308891	0.07285	0.067025	1.07843	3.64642	1.08697	0.02347	0.08746	0.02617	-0.8022	-0.51864	-0.79026	-0.6391	-0.6609	-0.7348	TNBC
		si2	0.06118	0.012887	0.072934	0.0434193	0.15098	0.078312	4.74713	1.67975	1.92787	0.10332	0.04029	0.04641	-0.44834	-0.72767	-0.70055				
SUM1315	TNBC	NTC	0.36345	0.041674	0.324764	0.0195322	0.28591	0.020035	8.7214	16.6271	14.2702	si/NTC									
		si1	0.17293	0.13402	0.221665	0.089112	0.16341	0.11422	1.29035	2.48749	1.43069	0.14795	0.1496	0.10026	-0.25054	-0.24322	-0.46191				
		si2	0.12476	0.165596	0.243093	0.2836748	0.12786	0.153741	0.75342	0.85694	0.83164	0.08639	0.05154	0.05828	-0.52338	-0.67781	-0.64795				
T47D	luminal	NTC	0.22166	0.005778	0.282156	0.0169868	0.37438	0.020273	38.3612	16.6103	18.4673	si/NTC						T1	T2	T3	
		si1	0.24309	0.087431	0.173446	0.0355521	0.5247	0.247767	2.78039	4.87865	2.11773	0.07248	0.29371	0.11467	-0.58501	0.39541	-0.39802				
		si2	0.28216	0.086429	0.188801	0.0194599	0.48393	0.063786	3.26459	9.70204	7.58687	0.0851	0.5841	0.41083	-0.52908	1.68229	0.91442				
MCF7	luminal	NTC	0.17345	0.005952	0.154054	0.0032943	0.19808	0.005544	29.1402	46.7643	35.7261	si/NTC									
		si1	0.17888	0.278839	0.162658	0.1092237	0.23378	0.034264	0.64152	1.48922	6.82285	0.02201	0.03185	0.19098	-0.80865	-0.76509	-0.05988	0.69297	0.86475	0.47707	luminal
		si2	0.15405	0.007152	0.154064	0.0077706	0.29322	0.048782	21.5414	19.8267	6.01082	0.73923	0.42397	0.16825	2.36978	0.97266	-0.1606				
ZR751	luminal	NTC	0.15578	0.074296	0.29758	0.1390272	0.1689	0.080932	2.09676	2.14045	2.08697	si/NTC									
		si1	0.15524	0.12481	0.155241	0.1382027	0.37021	0.357859	1.24381	1.12328	1.03452	0.59321	0.52479	0.49571	1.72267	1.41945	1.29057				
		si2	0.10298	0.075198	0.102976	0.089208	0.23093	0.224722	1.3694	1.15433	1.02763	0.6531	0.5393	0.4924	1.9881	1.48374	1.27594				
																		-1.3321	-1.5256	-1.2119	TNBC-luminal (Z-score)
																		0.000304039			P value

DOT1L flow records															Z-score			Average Z-score			
			Positive cells (100%)						HR/NHEJ ratio			Fold change of HR/NHEJ									
Cell lines		Group	T1 HR RFP	T1 NHEJ GFP	T2 HR RFP	T2 NHEJ GFP	T3 HR RFP	T3 NHEJ GFP	T1	T2	T3	T1	T2	T3	Avergae	SD		T1	T2	T3	
MDA-MB-231	TNBC	NTC	0.411242	0.027542	0.425457	0.034986	0.545716	0.03981	14.9315	12.1609	13.708		si/NTC		0.185004	0.21305					
		si1	0.0023	0.428178	0.01776	0.596652	0.0006	0.093686	0.00537	0.02977	0.0064	0.00036	0.00245	0.00047	-0.86667	-0.85687	-0.86616				
		si2	0.002063	0.497487	0.014874	0.650984	0.001339	0.261955	0.00415	0.02285	0.00511	0.00028	0.00188	0.00037	-0.86705	-0.85954	-0.86661				
BT549	TNBC	NTC	0.149051	0.003244	0.243093	0.00583	0.173446	0.004175	45.946	41.6937	41.5414		si/NTC								TNBC
		si1	0.033427	0.023018	0.162689	0.033873	0.086487	0.06529	1.4522	4.80297	1.32466	0.03161	0.1152	0.03189	-0.72	-0.32766	-0.71868	-0.48903	-0.52608	-0.64715	
		si2	0.080347	0.01232	0.105345	0.047613	0.179764	0.076336	6.5218	2.21252	2.35491	0.14194	0.05307	0.05669	-0.20211	-0.61928	-0.60228				
SUM1315	TNBC	NTC	0.363454	0.041674	0.324764	0.019532	0.285908	0.020035	8.7214	16.6271	14.2702		si/NTC								
		si1	0.227308	0.132821	0.320173	0.097719	0.194615	0.111476	1.71138	3.27645	1.74581	0.19623	0.19705	0.12234	0.052685	0.056564	-0.29413				
		si2	0.163964	0.164231	0.351124	0.311075	0.152161	0.150151	0.99838	1.12874	1.01339	0.11447	0.06789	0.07101	-0.33105	-0.54972	-0.53503				
T47D	luminal	NTC	0.221665	0.005778	0.282156	0.016987	0.374382	0.020273	38.3612	16.6103	18.4673		si/NTC					T1	T2	T3	
		si1	0.295258	0.112704	0.215836	0.046096	0.678465	0.318328	2.61976	4.68227	2.13134	0.06829	0.28189	0.11541	-0.54781	0.454757	-0.32664				
		si2	0.34272	0.111407	0.234944	0.025232	0.525709	0.381728	3.07629	9.31151	1.37718	0.08019	0.56059	0.07457	-0.49195	1.762883	-0.51832				
MCF7	luminal	NTC	0.173446	0.005952	0.154054	0.003294	0.198082	0.005544	29.1402	46.7643	35.7261		si/NTC								
		si1	0.217239	0.360536	0.252412	0.141618	0.302008	0.143763	0.60255	1.78234	2.10073	0.02068	0.03811	0.0588	-0.7713	-0.68946	-0.59236				luminal
		si2	0.187075	0.00876	0.111718	0.040075	0.378927	0.062434	21.3564	2.7877	6.06927	0.73289	0.05961	0.16988	2.571606	-0.58856	-0.07097	0.755414	0.666065	0.240784	
ZR751	luminal	NTC	0.155782	0.074296	0.29758	0.139027	0.168903	0.080932	2.09676	2.14045	2.08697		si/NTC								
		si1	0.188517	0.161102	0.193181	0.179192	0.378556	0.359906	1.17018	1.07807	1.05182	0.55809	0.50366	0.50399	1.751158	1.495708	1.497249				
		si2	0.125016	0.096864	0.128143	0.115666	0.298326	0.288693	1.29063	1.10787	1.03337	0.61553	0.51759	0.49515	2.020789	1.561059	1.455755				
																		-1.24445	-1.19215	-0.88793	TNBC-luminal (Z-score)
																			0.002604489		P value

GDPD2 flow records															Z-score			Average Z-score			
			Positive cells (100%)						HR/NHEJ ratio			Fold change of HR/NHEJ									
Cell lines		Group	T1 HR RFP	T1 NHEJ GFP	T2 HR RFP	T2 NHEJ GFP	T3 HR RFP	T3 NHEJ GFP	T1	T2	T3	T1	T2	T3	Avergae	SD		T1	T2	T3	
MDA-MB-231	TNBC	NTC	0.411242	0.027542	0.425457	0.034986	0.545716	0.03981	14.9315	12.1609	13.708	si/NTC			1.599321	0.323586					
		si1	0.121941	0.005536	0.139055	0.00552	0.436499	0.016331	22.0267	25.19	26.7287	1.475178	2.071401	1.94986	-0.38365	1.458903	1.083298				
		si2	0.275752	0.010224	0.054314	0.002428	0.281324	0.009758	26.9718	22.367	28.8315	1.806362	1.83926	2.103258	0.639834	0.741501	1.557355				
BT549	TNBC	NTC	0.149051	0.003244	0.243093	0.00583	0.173446	0.004175	45.946	41.6937	41.5414	si/NTC									
		si1	0.108873	0.002092	0.365996	0.007777	0.580615	0.010011	52.051	47.0616	57.9949	1.132872	1.128745	1.396075	-1.4415	-1.45425	-0.6281	-0.3972	0.08581	0.72981	TNBC
		si2	0.242032	0.004721	0.358838	0.006827	0.746495	0.013208	51.2654	52.5578	56.5194	1.115774	1.260568	1.360558	-1.49434	-1.04687	-0.73787				
SUM1315	TNBC	NTC	0.363454	0.041674	0.324764	0.019532	0.285908	0.020035	8.7214	16.6271	14.2702	si/NTC									
		si1	0.227725	0.01606	0.616256	0.023938	0.923477	0.030618	14.1793	25.7436	30.1614	1.625805	1.548291	2.11359	0.081845	-0.1577	1.589284				
		si2	0.077827	0.005347	0.073135	0.002298	0.371212	0.012449	14.5541	31.8288	29.8178	1.668783	1.914266	2.089516	0.214666	0.973298	1.514885				
T47D	luminal	NTC	0.221665	0.005778	0.282156	0.016987	0.374382	0.020273	38.3612	16.6103	18.4673	si/NTC						T1	T2	T3	
		si1	0.557458	0.012423	0.154125	0.005197	0.077691	0.001949	44.8747	29.6566	39.8719	1.169792	1.785431	2.15906	-1.3274	0.57515	1.729804				
		si2	0.050385	0.001057	0.175115	0.005168	0.089955	0.002801	47.6572	33.8821	32.1098	1.242327	2.039821	1.738744	-1.10324	1.361311	0.43087				
MCF7	luminal	NTC	0.173446	0.005952	0.154054	0.003294	0.198082	0.005544	29.1402	46.7643	35.7261	si/NTC									
		si1	0.12616	0.003211	0.094295	0.001729	0.781444	0.014006	39.2888	54.5308	55.7925	1.348266	1.166078	1.561673	-0.77585	-1.33888	-0.11634	-0.6346	-0.191	0.40722	luminal
		si2	0.094571	0.002194	0.318468	0.00636	0.06457	0.001304	43.1125	50.0769	49.5269	1.479483	1.070835	1.386296	-0.37034	-1.63322	-0.65833				
ZR751	luminal	NTC	0.155782	0.074296	0.29758	0.139027	0.168903	0.080932	2.09676	2.14045	2.08697	si/NTC									
		si1	0.064047	0.018449	0.347072	0.107372	0.165377	0.044029	3.47157	3.23243	3.75611	1.655682	1.510164	1.799789	0.174178	-0.27553	0.619522				
		si2	0.257506	0.083643	0.215478	0.060913	0.108121	0.029758	3.07863	3.53746	3.63339	1.468281	1.652674	1.740985	-0.40496	0.164883	0.437797				
																		0.23741	0.27686	0.32259	TNBC-luminal (Z-score)
																		0.564424072			P value

GTF2H5 flow records															Z-score			Average Z-score			
			Positive cells (100%)						HR/NHEJ ratio			Fold change of HR/NHEJ ratio									
Cell lines		Group	T1 HR RFP	T1 NHEJ GFP	T2 HR RFP	T2 NHEJ GFP	T3 HR RFP	T3 NHEJ GFP	T1	T2	T3	T1	T2	T3	Avergae	SD		T1	T2	T3	
MDA-MB-231	TNBC	NTC	0.411242	0.027542	0.425457	0.034986	0.545716	0.03981	14.93155	12.16087	13.70802	si/NTC			0.17572	0.17473					
		si1	0.621606	0.06794	0.305263	0.149369	0.40225	0.046515	9.149351	2.043677	8.647836	0.61275	0.16805	0.63086	2.50128	-0.0439	2.60491				
		si2	0.00147	0.789176	0.0044	0.599379	0.204338	0.117607	0.001863	0.00734	1.737461	0.00012	0.0006	0.12675	-1.005	-1.0022	-0.2803				
BT549	TNBC	NTC	0.149051	0.003244	0.243093	0.00583	0.173446	0.004175	45.94604	41.69372	41.54135	si/NTC									TNBC
		si1	0.119454	0.037679	0.448619	0.031368	0.20236	0.118128	3.170263	14.30201	1.713057	0.069	0.34303	0.04124	-0.6108	0.95756	-0.7697	-0.20184	0.042703	-0.0401	
		si2	0.046357	0.020735	0.031465	0.044015	0.419288	0.092882	2.235712	0.714864	4.514224	0.04866	0.01715	0.10867	-0.7272	-0.9075	-0.3837				
SUM1315	TNBC	NTC	0.363454	0.041674	0.324764	0.019532	0.285908	0.020035	8.721403	16.62714	14.2702	si/NTC									
		si1	0.130622	0.211593	0.695731	0.090134	0.453825	0.480034	0.617327	7.718832	0.945402	0.07078	0.46423	0.06625	-0.6006	1.65125	-0.6265				
		si2	0.094302	0.261343	0.504991	0.286514	0.355093	0.646468	0.360836	1.762532	0.549281	0.04137	0.106	0.03849	-0.7689	-0.399	-0.7854				
T47D	luminal	NTC	0.221665	0.005778	0.282156	0.016987	0.374382	0.020273	38.36124	16.61031	18.46725	si/NTC						T1	T2	T3	
		si1	0.132141	0.155673	0.109585	0.043185	0.112171	0.118557	0.848837	2.537604	0.946136	0.02213	0.15277	0.05123	-0.879	-0.1313	-0.7124				
		si2	0.169407	0.153894	0.119291	0.023724	0.203455	0.235747	1.100804	5.028357	0.863023	0.0287	0.30273	0.04673	-0.8414	0.72691	-0.7382				
MCF7	luminal	NTC	0.173446	0.005952	0.154054	0.003294	0.198082	0.005544	29.14024	46.76433	35.72607	si/NTC									
		si1	0.170882	0.495537	0.102766	0.093228	0.499802	0.126184	0.344841	1.102312	3.960908	0.01183	0.02357	0.11087	-0.9379	-0.8708	-0.3711				luminal
		si2	0.147197	0.013128	0.097334	0.009587	0.226871	0.180065	11.21224	10.15245	1.25994	0.38477	0.2171	0.03527	1.19646	0.23684	-0.8038	-0.00918	0.187806	0.020616	
ZR751	luminal	NTC	0.155782	0.074296	0.29758	0.139027	0.168903	0.080932	2.096759	2.140448	2.086971	si/NTC									
		si1	0.114833	0.222043	0.098078	0.167324	0.391458	0.432714	0.517166	0.586154	0.904657	0.24665	0.27385	0.43348	0.40598	0.56163	1.47525				
		si2	0.098469	0.133951	0.065041	0.108073	0.193719	0.233032	0.735112	0.601826	0.831299	0.35059	0.28117	0.39833	1.00088	0.60353	1.27407				
																		-0.19266	-0.1451	-0.06072	TNBC-luminal (Z-score)
																		0.23222259			P value

MCTS1 flow records															Z-score			Average Z-score			
			Positive cells (100%)						HR/NHEJ ratio			Fold change of HR/NHEJ ratio									
Cell lines		Group	T1 HR RFP	T1 NHEJ GFP	T2 HR RFP	T2 NHEJ GFP	T3 HR RFP	T3 NHEJ GFP	T1	T2	T3	T1	T2	T3	Avergae	SD		T1	T2	T3	
MDA-MB-231	TNBC	NTC	0.411242	0.027542	0.425457	0.034986	0.545716	0.03981	14.93155	12.16087	13.70802	si/NTC			2.2092	0.953821		0.28778	0.47504	0.45725	TNBC
		si1	0.063371	0.001227	0.132015	0.003069	0.590607	0.012507	51.63441	43.01732	47.2215	3.458075	3.537355	3.444808	1.309339	1.392458	1.29543				
		si2	0.147901	0.002944	0.051722	0.001316	0.380433	0.008422	50.23937	39.29191	45.16907	3.364646	3.231011	3.295083	1.211386	1.071282	1.138456				
BT549	TNBC	NTC	0.149051	0.003244	0.243093	0.00583	0.173446	0.004175	45.94604	41.69372	41.54135	si/NTC									
		si1	0.067449	0.000886	0.267041	0.004348	0.385802	0.004658	76.13043	61.41731	82.83398	1.656953	1.473059	1.994012	-0.57898	-0.77178	-0.22561				
		si2	0.364985	0.005929	0.280259	0.00381	0.144746	0.002116	61.56043	73.56215	68.40015	1.339842	1.764346	1.646556	-0.91145	-0.46639	-0.58988				
SUM1315	TNBC	NTC	0.363454	0.041674	0.324764	0.019532	0.285908	0.020035	8.721403	16.62714	14.2702	si/NTC									
		si1	0.087753	0.005081	0.584162	0.013508	0.250185	0.007733	17.26969	43.24516	32.35429	1.980151	2.600878	2.267262	-0.24014	0.410641	0.060874				
		si2	0.031339	0.001158	0.069555	0.001242	0.50218	0.010914	27.05813	55.98596	46.01157	3.102498	3.367143	3.224311	0.936547	1.214004	1.064257				
T47D	luminal	NTC	0.221665	0.005778	0.282156	0.016987	0.374382	0.020273	38.36124	16.61031	18.46725	si/NTC						T1	T2	T3	luminal
		si1	0.219348	0.003749	0.126293	0.003886	0.104627	0.003219	58.50634	32.50247	32.49927	1.525142	1.956765	1.759832	-0.71718	-0.26466	-0.47112	-0.3876	-0.4313	-0.4011	
		si2	0.044742	0.000613	0.116181	0.002869	0.111238	0.002683	73.00745	40.48934	41.45555	1.903157	2.437603	2.244814	-0.32086	0.239462	0.037339				
MCF7	luminal	NTC	0.173446	0.005952	0.154054	0.003294	0.198082	0.005544	29.14024	46.76433	35.72607	si/NTC									
		si1	0.066238	0.003759	0.189604	0.008501	0.047811	0.001636	17.62118	22.30346	29.23262	0.604703	0.476933	0.818243	-1.68218	-1.81613	-1.4583				
		si2	0.04477	0.003289	0.102008	0.003545	0.186855	0.005969	13.61298	28.77837	31.30683	0.467154	0.615392	0.876302	-1.82639	-1.67097	-1.39743				
ZR751	luminal	NTC	0.155782	0.074296	0.29758	0.139027	0.168903	0.080932	2.096759	2.140448	2.086971	si/NTC									
		si1	0.044026	0.006156	0.32911	0.060798	0.223392	0.040147	7.151743	5.413146	5.564333	3.410856	2.528978	2.666225	1.259834	0.335261	0.479152				
		si2	0.195501	0.02983	0.204426	0.034466	0.145842	0.026936	6.553825	5.931293	5.414331	3.125693	2.771053	2.594349	0.960865	0.589055	0.403796				
																		0.67543	0.90637	0.85835	TNBC-luminal (Z-score)
																		0.000183555			P value

NFKBIA flow records																Z-score			Average Z-score			
			Positive cells (100%)						HR/NHEJ ratio			Fold change of HR/NHEJ ratio										
Cell lines		Group	T1 HR RFP	T1 NHEJ GFP	T2 HR RFP	T2 NHEJ GFP	T3 HR RFP	T3 NHEJ GFP	T1	T2	T3	T1	T2	T3	Avergae	SD		T1	T2	T3	
MDA-MB-231	TNBC	NTC	0.411242	0.027542	0.425457	0.034986	0.545716	0.03981	14.9315	12.1609	13.708	si/NTC			0.178212	0.196879					
		si1	0.195935	0.722198	0.130535	0.802571	0.093032	0.158253	0.2713	0.16265	0.58787	0.01817	0.013375	0.042885	-0.8129	-0.83725	-0.68736				
		si2	0.185428	0.838291	0.22541	0.875746	0.10122	0.044069	0.2212	0.25739	2.29685	0.014814	0.021166	0.167555	-0.82994	-0.79768	-0.05413				
BT549	TNBC	NTC	0.149051	0.003244	0.243093	0.00583	0.173446	0.004175	45.946	41.6937	41.5414	si/NTC									
		si1	0.072734	0.004356	0.287871	0.04462	0.207273	0.11059	16.6992	6.45166	1.87425	0.363453	0.154739	0.045118	0.940883	-0.11922	-0.67602	-0.247	-0.3881	-0.3849	TNBC
		si2	0.173425	0.025636	0.186051	0.063125	0.435671	0.12913	6.765	2.94733	3.37389	0.147238	0.07069	0.081218	-0.15733	-0.54613	-0.49266				
SUM1315	TNBC	NTC	0.363454	0.041674	0.324764	0.019532	0.285908	0.020035	8.7214	16.6271	14.2702	si/NTC									
		si1	0.488802	0.627475	0.567499	0.130608	0.472035	0.188112	0.779	4.34504	2.50933	0.08932	0.261322	0.175844	-0.45151	0.422137	-0.01203				
		si2	0.352868	0.280087	0.622456	0.417956	0.368082	0.253028	1.25985	1.48929	1.45471	0.144455	0.08957	0.10194	-0.17146	-0.45024	-0.38741				
T47D	luminal	NTC	0.221665	0.005778	0.282156	0.016987	0.374382	0.020273	38.3612	16.6103	18.4673	si/NTC						T1	T2	T3	
		si1	0.575573	0.196034	0.339072	0.162705	0.181864	0.535313	2.93609	2.08396	0.33973	0.076538	0.125462	0.018397	-0.51643	-0.26793	-0.81175				
		si2	0.667933	0.193835	0.369178	0.13387	0.214755	0.241731	3.44589	2.75773	0.88841	0.089827	0.166025	0.048107	-0.44893	-0.0619	-0.66084				
MCF7	luminal	NTC	0.173446	0.005952	0.154054	0.003294	0.198082	0.005544	29.1402	46.7643	35.7261	si/NTC									
		si1	0.423748	0.616108	0.3967	0.194717	0.523591	0.242307	0.68778	2.03732	2.16086	0.023602	0.043566	0.060484	-0.7853	-0.6839	-0.59797	0.46117	0.42338	0.1355	luminal
		si2	0.365048	0.119848	0.175022	0.054384	0.658091	0.25795	3.04594	3.21827	2.55124	0.104527	0.068819	0.071411	-0.37427	-0.55564	-0.54247				
ZR751	luminal	NTC	0.155782	0.074296	0.29758	0.139027	0.168903	0.080932	2.09676	2.14045	2.08697	si/NTC									
		si1	0.367855	0.278067	0.303377	0.246644	0.657444	0.605103	1.3229	1.23002	1.0865	0.630925	0.574655	0.520611	2.299447	2.013637	1.739129				
		si2	0.24428	0.169185	0.200902	0.158851	0.517153	0.485571	1.44386	1.26472	1.06504	0.688617	0.590869	0.510329	2.592478	2.095988	1.686907				
																		-0.7082	-0.8114	-0.5204	TNBC-luminal (Z-score)
																		0.003822699			P value

SLC31A1 flow records																Z-score			Average Z-score			
			Positive cells (100%)						HR/NHEJ ratio			Fold change of HR/NHEJ ratio									
Cell lines		Group	T1 HR RFP	T1 NHEJ GFP	T2 HR RFP	T2 NHEJ GFP	T3 HR RFP	T3 NHEJ GFP	T1	T2	T3	T1	T2	T3	Avergae	SD		T1	T2	T3	
	TNBC	NTC	0.411242	0.027542	0.425457	0.034986	0.545716	0.03981	14.9315	12.1609	13.708	si/NTC			1.366555	0.172425					
MDA-MB-231		si1	0.343183	0.013591	0.16377	0.008369	0.281195	0.015049	25.2511	19.5694	18.6851	1.691122	1.609209	1.363079	1.882359	1.407293	-0.02016				
		si2	0.543913	0.023646	0.0626	0.003732	0.181368	0.00921	23.0024	16.7753	19.6927	1.540525	1.379446	1.436581	1.008952	0.074762	0.40612				
	TNBC	NTC	0.149051	0.003244	0.243093	0.00583	0.173446	0.004175	45.946	41.6937	41.5414	si/NTC									TNBC
BT549		si1	0.394018	0.007704	0.196271	0.003956	0.502573	0.009436	51.1438	49.6166	53.2639	1.113128	1.190025	1.282189	-1.46978	-1.02381	-0.48929	0.26458	0.00632	0.02313	
		si2	0.589254	0.011843	0.4231	0.008829	0.609287	0.012275	49.756	47.9191	49.6368	1.082922	1.149313	1.194876	-1.64496	-1.25992	-0.99567				
	TNBC	NTC	0.363454	0.041674	0.324764	0.019532	0.285908	0.020035	8.7214	16.6271	14.2702	si/NTC									
SUM1315		si1	0.547317	0.036166	0.726838	0.03599	0.59448	0.027741	15.1337	20.1955	21.4296	1.735232	1.214613	1.5017	2.138178	-0.88121	0.783784				
		si2	0.150667	0.013186	0.097787	0.003536	0.239195	0.011601	11.4261	27.6554	20.6182	1.310127	1.663266	1.44484	-0.32726	1.720803	0.45402				
	luminal	NTC	0.221665	0.005778	0.282156	0.016987	0.374382	0.020273	38.3612	16.6103	18.4673	si/NTC						T1	T2	T3	
T47D		si1	0.615869	0.011202	0.181552	0.007884	0.050366	0.001918	54.9766	23.0281	26.2666	1.43313	1.386374	1.422336	0.386105	0.11494	0.323507				
		si2	0.086068	0.001489	0.206318	0.007841	0.058256	0.002142	57.7941	26.3128	27.1942	1.506574	1.584127	1.472565	0.812054	1.261829	0.614811				
	luminal	NTC	0.173446	0.005952	0.154054	0.003294	0.198082	0.005544	29.1402	46.7643	35.7261	si/NTC									
MCF7		si1	0.277947	0.006244	0.110956	0.001933	0.580305	0.012984	44.5151	57.3883	44.6929	1.527617	1.227182	1.250989	0.934091	-0.80831	-0.67024	0.07712	-0.2398	-0.1314	luminal
		si2	0.162303	0.003723	0.340068	0.005728	0.673107	0.0139	43.5917	59.3672	48.4243	1.495929	1.269498	1.355432	0.750317	-0.5629	-0.06451				
	luminal	NTC	0.155782	0.074296	0.29758	0.139027	0.168903	0.080932	2.09676	2.14045	2.08697	si/NTC									
ZR751		si1	0.109639	0.041289	0.409217	0.161116	0.106777	0.038766	2.6554	2.5399	2.75437	1.266432	1.186621	1.319794	-0.58068	-1.04355	-0.2712				
		si2	0.398561	0.18113	0.253945	0.091441	0.069943	0.026977	2.20041	2.77713	2.59269	1.049433	1.297452	1.24232	-1.83918	-0.40077	-0.72051				
																		0.18746	0.24611	0.15449	TNBC-luminal (Z-score)
																		0.19171173			P value

TAF8 flow records															Z-score			Average Z-score			
			Positive cells (100%)						HR/NHEJ ratio			Fold change of HR/NHEJ ratio									
Cell lines		Group	T1 HR RFP	T1 NHEJ GFP	T2 HR RFP	T2 NHEJ GFP	T3 HR RFP	T3 NHEJ GFP	T1	T2	T3	T1	T2	T3	Avergae	SD		T1	T2	T3	
MDA-MB-231	TNBC	NTC	0.411242	0.027542	0.425457	0.034986	0.545716	0.03981	14.9315	12.1609	13.708	si/NTC			0.145224	0.149048					
		si1	0.421477	0.503561	0.200243	0.078617	0.139266	0.294796	0.83699	2.54707	0.47241	0.056055	0.209448	0.034463	-0.59825	0.430891	-0.74313				
		si2	0.398928	0.744453	0.213828	0.640581	0.151128	0.082814	0.53587	0.3338	1.8249	0.035888	0.027449	0.133126	-0.73356	-0.79018	-0.08117				
BT549	TNBC	NTC	0.149051	0.003244	0.243093	0.00583	0.173446	0.004175	45.946	41.6937	41.5414	si/NTC									TNBC
		si1	0.157086	0.014038	0.172273	0.036784	0.304755	0.20631	11.1903	4.68337	1.47718	0.243554	0.112328	0.035559	0.659717	-0.22071	-0.73577	-0.2174	0.030616	-0.43261	
		si2	0.373171	0.058194	0.163301	0.010525	0.635613	0.24073	6.41253	15.5162	2.64036	0.139567	0.372148	0.06356	-0.03796	1.522485	-0.54791				
SUM1315	TNBC	NTC	0.363454	0.041674	0.324764	0.019532	0.285908	0.020035	8.7214	16.6271	14.2702	si/NTC									
		si1	0.04997	0.107011	0.094392	0.059599	0.688289	0.350229	0.46696	1.58377	1.96525	0.053542	0.095252	0.137717	-0.61512	-0.33527	-0.05037				
		si2	0.758254	0.586181	0.481776	0.352925	0.537703	0.470746	1.29355	1.3651	1.14224	0.148319	0.0821	0.080043	0.020763	-0.42351	-0.43731				
T47D	luminal	NTC	0.221665	0.005778	0.282156	0.016987	0.374382	0.020273	38.3612	16.6103	18.4673	si/NTC						T1	T2	T3	
		si1	0.330377	0.215736	0.195891	0.16932	0.031575	0.127153	1.53139	1.15693	0.24832	0.03992	0.069651	0.013447	-0.70651	-0.50704	-0.88413				
		si2	0.499278	0.213372	0.322252	0.139135	0.172643	0.328908	2.33994	2.31611	0.5249	0.060997	0.139438	0.028423	-0.5651	-0.03882	-0.78365				
MCF7	luminal	NTC	0.173446	0.005952	0.154054	0.003294	0.198082	0.005544	29.1402	46.7643	35.7261	si/NTC									
		si1	0.317116	0.667316	0.34635	0.202829	0.258256	0.129689	0.47521	1.7076	1.99135	0.016308	0.036515	0.055739	-0.86493	-0.72936	-0.60038				luminal
		si2	0.273326	0.133836	0.15225	0.055929	0.226193	0.250909	2.04224	2.72219	0.90149	0.070083	0.058211	0.025234	-0.50414	-0.58379	-0.80505	0.241306	0.455773	-0.07769	
ZR751	luminal	NTC	0.155782	0.074296	0.29758	0.139027	0.168903	0.080932	2.09676	2.14045	2.08697	si/NTC									
		si1	0.27542	0.303922	0.264637	0.257187	0.325384	0.331822	0.90622	1.02897	0.9806	0.432199	0.480725	0.469867	1.925384	2.250957	2.178105				
		si2	0.183233	0.186874	0.17491	0.165285	0.205022	0.469677	0.98052	1.05823	0.43652	0.467635	0.494398	0.209163	2.163128	2.342691	0.428979				
																		-0.45871	-0.42516	-0.35492	TNBC-luminal (Z-score)
																		0.113941032			P value

ZDHHC9 flow records																	Z-score			Average Z-score			
			Positive cells (100%)						HR/NHEJ ratio			Fold change of HR/NHEJ ratio									
Cell lines		Group	T1 HR RFP	T1 NHEJ GFP	T2 HR RFP	T2 NHEJ GFP	T3 HR RFP	T3 NHEJ GFP	T1	T2	T3	T1	T2	T3	Avergae	SD		T1	T2	T3	
MDA-MB-231	TNBC	NTC	0.411242	0.027542	0.425457	0.034986	0.545716	0.03981	14.9315	12.1609	13.708	si/NTC			1.119607	0.098703					
		si1	0.551689	0.032179	0.638382	0.039143	0.332757	0.02199	17.1443	16.309	15.1321	1.148196	1.341105	1.103884	0.289645	2.244087	-0.1593				
		si2	0.498993	0.032756	0.592359	0.034006	0.214897	0.013625	15.2337	17.4192	15.7728	1.020234	1.432395	1.150628	-1.0068	3.168995	0.314278				
BT549	TNBC	NTC	0.149051	0.003244	0.243093	0.00583	0.173446	0.004175	45.946	41.6937	41.5414	si/NTC									TNBC
		si1	0.12614	0.002624	0.0916	0.002092	0.594124	0.013948	48.0711	43.7799	42.5964	1.04625	1.050036	1.025397	-0.74321	-0.70486	-0.95449	-0.30876	0.859425	0.023949	
		si2	0.50709	0.010105	0.502847	0.011264	0.731921	0.016583	50.1838	44.6415	44.1369	1.092233	1.0707	1.062481	-0.27734	-0.4955	-0.57878				
SUM1315	TNBC	NTC	0.363454	0.041674	0.324764	0.019532	0.285908	0.020035	8.7214	16.6271	14.2702	si/NTC									
		si1	0.604022	0.059159	0.699148	0.038268	0.702632	0.040173	10.2102	18.2697	17.4899	1.170703	1.09879	1.225627	0.517673	-0.21091	1.074133				
		si2	0.35421	0.038417	0.102696	0.005007	0.28317	0.01705	9.22007	20.511	16.6078	1.057178	1.233584	1.163811	-0.6325	1.154752	0.447847				
T47D	luminal	NTC	0.221665	0.005778	0.282156	0.016987	0.374382	0.020273	38.3612	16.6103	18.4673	si/NTC						T1	T2	T3	
		si1	0.727004	0.018007	0.173768	0.00933	0.060232	0.003177	40.3737	18.625	18.9591	1.052461	1.121291	1.026633	-0.68029	0.017061	-0.94197				
		si2	0.616299	0.015396	0.19763	0.010281	0.069547	0.003499	40.0303	19.2222	19.8768	1.043508	1.157245	1.076328	-0.771	0.381327	-0.43849				
MCF7	luminal	NTC	0.173446	0.005952	0.154054	0.003294	0.198082	0.005544	29.1402	46.7643	35.7261	si/NTC									
		si1	0.414393	0.013669	0.1044	0.002146	0.685898	0.019032	30.3173	48.6532	36.0393	1.040393	1.040392	1.008769	-0.80256	-0.80257	-1.12296				luminal
		si2	0.612442	0.018992	0.384895	0.007737	0.783657	0.020344	32.2472	49.7458	38.52	1.106622	1.063755	1.078205	-0.13156	-0.56587	-0.41947	-0.61662	0.332389	-0.29038	
ZR751	luminal	NTC	0.155782	0.074296	0.29758	0.139027	0.168903	0.080932	2.09676	2.14045	2.08697	si/NTC									
		si1	0.091238	0.037817	0.393122	0.139805	0.130257	0.052491	2.41259	2.81194	2.48151	1.150627	1.313714	1.189048	0.314278	1.966583	0.703538				
		si2	0.391715	0.194834	0.243518	0.0934	0.095151	0.039079	2.0105	2.60726	2.43485	0.958861	1.218093	1.166692	-1.62859	0.997798	0.47703				
																		0.307866	0.527037	0.314334	TNBC-luminal (Z-score)
																		0.438087845			P value
